# Somatic Copy-Number Alterations in Plasma Circulating Tumor DNA from Advanced EGFR-Mutated Lung Adenocarcinoma Patients

**DOI:** 10.3390/biom11050618

**Published:** 2021-04-21

**Authors:** Anna Buder, Ellen Heitzer, Julie Waldispühl-Geigl, Sabrina Weber, Tina Moser, Maximilian J. Hochmair, Klaus Hackner, Peter Errhalt, Ulrike Setinek, Martin Filipits

**Affiliations:** 1 Comprehensive Cancer Center, Institute of Cancer Research, Department of Medicine I, Medical University of Vienna, 1090 Vienna, Austria; anna_buder@gmx.at; 2 Diagnostic and Research Center for Molecular BioMedicine, Institute of Human Genetics, Medical University of Graz, 8036 Graz, Austria; ellen.heitzer@medunigraz.at (E.H.); julie.geigl@medunigraz.at (J.W.-G.); sabrina.weber@medunigraz.at (S.W.); tina.moser@medunigraz.at (T.M.); 3 Christian Doppler Laboratory for Liquid Biopsies for Early Detection of Cancer, 8036 Graz, Austria; 4 Karl Landsteiner Institute of Lung Research and Pulmonary Oncology, Department of Respiratory and Critical Care Medicine, Hospital North, 1210 Vienna, Austria; maximilian.hochmair@gesundheitsverbund.at; 5 Department of Pneumology, University Hospital Krems, Karl Landsteiner University of Health Sciences, 3500 Krems, Austria; klaus.hackner@krems.lknoe.at (K.H.); peter.errhalt@krems.lknoe.at (P.E.); 6 Department of Pathology and Bacteriology, Otto Wagner Hospital, 1140 Vienna, Austria; ulrike.setinek@wienkav.at

**Keywords:** NSCLC, osimertinib, somatic copy-number alterations, ctDNA, *EGFR* mutations

## Abstract

Background: To assess the clinical relevance of genome-wide somatic copy-number alterations (SCNAs) in plasma circulating tumor DNA (ctDNA) from advanced epidermal growth factor receptor (*EGFR*)-mutated lung adenocarcinoma patients. Methods: We included 43 patients with advanced *EGFR* T790M-positive lung adenocarcinoma who were treated with osimertinib after progression under previous EGFR-TKI therapy. We performed genomic profiling of ctDNA in plasma samples from each patient obtained pre-osimertinib and after patients developed resistance to osimertinib. SCNAs were detected by shallow whole-genome plasma sequencing and *EGFR* mutations were assessed by droplet digital PCR. Results: SCNAs in resistance-related genes (rrSCNAs) were detected in 10 out of 31 (32%) evaluable patients before start of osimertinib. The presence of rrSCNAs in plasma before the initiation of osimertinib therapy was associated with a lower response rate to osimertinib (50% versus 81%, *p* = 0.08) and was an independent predictor for shorter progression-free survival (adjusted HR 3.33, 95% CI 1.37–8.10, *p* = 0.008) and overall survival (adjusted HR 2.54, 95% CI 1.09–5.92, *p* = 0.03). Conclusions: Genomic profiling of plasma ctDNA is clinically relevant and affects the efficacy and clinical outcome of osimertinib. Our approach enables the comprehensive assessment of SCNAs in plasma samples of lung adenocarcinoma patients and may help to guide genotype-specific therapeutic strategies in the future.

## 1. Introduction

Osimertinib is the standard treatment of advanced epidermal growth factor receptor (*EGFR*)-mutated non-small cell lung cancer (NSCLC) patients and *EGFR* T790M-mediated resistance [1,2,3,4]. Despite high response rates, patients will develop resistance to osimertinib therapy and clinically progress. Resistance mechanisms in osimertinib-treated patients appear to be complex and are currently not fully understood. Both EGFR-dependent and EGFR-independent mechanisms of resistance may be important [5].

Comprehensive tumor tissue and plasma analyses of patients who progressed under osimertinib treatment revealed insights into various mechanisms of resistance, including novel *EGFR* resistance mutations [6,7,8,9,10,11,12], *EGFR* amplification [13,14], the activation of bypass pathways via *ERBB2* amplification [15,16], *MET* amplification [7,13,15,16,17,18], *RAS* mutations [7,13,16,19], *BRAF* mutations [7,17,20], *PIK3CA* mutations [7,13], *CDK4/CDK6/CDKN2A* alterations [21], and transformation into small-cell lung cancer [7,22,23,24]. Strategies to understand and overcome these resistance mechanisms, e.g., by combination therapies are currently being explored in clinical trials [5].

Due to the fact that a liquid biopsy is less burdensome than a tissue biopsy and that in many advanced NSCLC patients, multiple tissue sampling is clinically not feasible, we selected plasma for molecular profiling [25,26]. Blood samples are easily obtainable and can be taken repeatedly even in short time intervals. In addition, the genetic heterogeneity of the progressing tumor may lead to an incomplete picture of the tumor genome if only single tissue biopsies are obtained. Furthermore, blood-based analytic approaches may allow for real-time monitoring of the total tumor burden and the detection of upcoming mutations that arise during clinical treatment through serial blood sampling and analysis. Blood samples can be collected during routine care at the time of diagnosis, before first-line therapy, and at subsequent time points when the tumor is progressing on therapy.

In this study, genome-wide copy number profiling with a special focus on focal events was performed using shallow whole-genome sequencing in circulating tumor DNA (ctDNA) of plasma samples from each patient collected prior to osimertinib initiation and at the time of osimertinib resistance in order to detect molecular alterations relevant for therapy efficacy. Here, we report the results of this study.

## 2. Material and Methods

### 2.1. Patient Cohort and Sample Collection

Samples of 43 patients with advanced *EGFR*-mutated lung adenocarcinoma who progressed under first- or second- generation EGFR-TKI therapy were collected between August 2015 and January 2019. All patients developed the T790M resistance mutation and were treated with osimertinib. Patients had a confirmed activating *EGFR* mutation at the time of initial diagnosis in tissue biopsy. The first plasma sample was collected at the time of radiologic progression to a first- or second-generation EGFR TKI (“pre-osimertinib” sample). A second plasma sample was collected from all patients at the time of clinical progression under osimertinib. In addition, a set of 10 self-reporting healthy individuals (an age range of 20–30 years) was analyzed.

### 2.2. Blood Collection and Cell-Free DNA Extraction from Plasma

Blood processing was performed as previously described [27]. Briefly, EDTA-containing vacutainer tubes or cell-free DNA Blood Collection Tubes (Roche, Pleasanton, CA, USA) were used for blood collection. For plasma isolation, whole blood was centrifuged at 200× *g* for 10 min, followed by 1600× *g* for 10 min. Subsequently, the supernatant was collected and centrifuged at 1900× *g* for 10 min. ctDNA was extracted from 2 mL of plasma using the QIAamp circulating nucleic acid kit (Qiagen, Hilden, Germany), according to the manufacturer’s instructions.

### 2.3. ddPCR

*EGFR* exon 19 deletion, L858R, T790M and C797S mutations were assessed using custom-made ddPCR assays of Life Technologies (Carlsbad, CA, USA). *EGFR* L861Q mutations were detected by means of a ddPCR assay of Bio-Rad (Hercules, CA, USA). Primer sequences and PCR protocols were previously specified [27,28,29,30]. All ddPCR assays were performed in triplicate and analyzed with QuantaSoft analysis software (Bio-Rad). Results were reported as copies of mutant allele per ml of plasma. The threshold for positivity was >1 copy/mL for all assays.

### 2.4. Shallow Whole-Genome Plasma Sequencing

Shallow whole-genome plasma sequencing was performed as previously described [31]. Briefly, a total of 5–10 ng of input DNA from plasma DNA extractions was used based on ctDNA quantification using the Qubit dsDNA HS Assay kit (Life Technologies, Carlsbad, CA, USA). Shotgun libraries were prepared using the TruSeq Nano DNA HT Sample preparation kit (Illumina, San Diego, CA, USA). Due to the high fragmentation of plasma DNA, the fragmentation step was omitted and for the selective amplification of the library fragments, 20 PCR cycles were used.

Plasma DNA libraries were quantified and normalized with quantitative PCR, using primers complementary to Illumina-specific adaptor sequences (forward: AATGATAC GGCGACCACCGAGAT; reverse: CAAGCAGAAGACGGCATACGA). Libraries were pooled equimolarly and sequenced on an Illumina MiSeq or NextSeq instrument (Illumina) either in a paired end sequencing mode (2 × 75 bp) or single read mode (150 bp). Sequencing reads were analyzed using the plasma-Seq algorithm, which is based on read count analysis to establish genome-wide SCNAs [31]. Briefly, sequencing reads were mapped to the PAR-masked genome, counted in non-overlapping 50 kb windows and normalized by the total amount of reads. After GC normalization, read counts were further normalized to healthy controls to avoid position effects and we normalized the sequencing. The resulting normalized log2 ratios were segmented. Since driver genes and copy number alterations related to resistance are frequently located on focal amplifications, we specifically called for focal events as previously described in detail [32].

Focal amplifications were defined as follows:

1. Segment should be <20 Mb;
2. Log2-ratio must be >0.2;
3. Segment should contain a gene, but not >100 genes;
4. Log2-ratio must be 0.2 higher than weighted mean of the log2-ratios of neighboring 20Mb on both the sides if it contains a known tumor driver gene;
5. Log2-ratio must be 0.58 higher (Log2-ratio of 0.58 translates to about three copies) than weighted mean of the log2-ratios of neighboring 20Mb on both the sides if it does not contain a known tumor driver gene;
6. Segment should not contain segmental duplications in >50% of its size;
7. Segment should not overlap with known entries in DGVar.

For focal deletions, the following criteria were used:

1. Segment should be <20 Mb;
2. Log2-ratio must be lower than −0.2;
3. Segment should contain a gene known to be affected by deletions;
4. Segment should contain a gene but not >100 genes;
5. Log2-ratio must be 0.2 lower than weighted mean of the log2-ratios of neighboring 20 Mb on both the sides;
6. Segment should not contain segmental duplications in >50% of its size;
7. Segment should not overlap with known entries in DGVar.

Focal identification was performed using R.

To calculate the tumor fraction (TF) in plasma DNA samples, data were analyzed with the previously published ichorCNA algorithm using a 1Mb bin size [33]. As a cut-off to reliably detect focal SCNAs, we set the detection threshold to a TF of 3% [33]. Focal SCNAs, in which—based on a literature review—genes that may be associated with resistance to osimertinib were located, were defined as resistance-related SCNA (rrSCNAs). To assess the background noise, 10 self-reporting healthy individuals with a similar number of reads (average number of reads for cases 6,569,227, range 4,652,614–8,877,744; average number of reads for controls 6,917,896, range 6,833,913–6,981,005) were analyzed.

### 2.5. Statistical Analyses

Progression-free survival (PFS) was determined by investigator assessment and was defined as the duration between the first osimertinib dose and progression of disease or death for any cause, whichever occurred first. The overall survival (OS) was calculated from first osimertinib dose to death from any cause.

Tumor response was assessed by contrast enhanced computed tomography of the chest and abdomen by the local radiologist according to institutional practice. The scan intervals were usually between 6 to 8 weeks at the treating physician’s discretion. The response rate (RR) was defined as the percentage of patients showing complete response (CR) or partial response (PR) at restaging after osimertinib initiation.

Patient and tumor characteristics included age, sex, presence or absence of extra-thoracic metastases, tissue genotype at diagnosis, previous EGFR-TKI therapy, and tumor fraction in plasma DNA samples. Chi-square test and Fisher’s exact test were used to evaluate the association of rrSCNAs with clinical parameters including response to osimertinib. Logistic regression models were used to assess the independent effect of covariables on response. Age and the values of TF in plasma DNA samples were compared by Mann–Whitney-U tests. The Kaplan–Meier method was used to estimate survival probabilities. Differences between survival curves were analyzed by means of the log-rank test. Univariate and multivariate Cox-proportional hazards regression models were used to compare survival outcome according to rrSCNAs. For the multivariate analyses, we used stepwise backward elimination models that included age (as continuous variable), gender (male, female), presence or absence of extra-thoracic metastases (thoracic, extra-thoracic), tissue genotype at diagnosis (EGFR deletions in exon 19, L858R, L861Q), previous EGFR TKI therapy (afatinib, erlotinib, gefitinib, >1 EGFR TKI), TF (as continuous variable), and rrSCNA (present, absent).

All reported *p*-values are 2-sided and considered significant at the 0.05 level. Statistical analyses were performed using SPSS Statistics software, version 25 (SPSS, IBM Corporation, Armonk, NY, USA).

## 3. Results

### 3.1. Patient Characteristics

Plasma samples of 43 advanced *EGFR* T790M-positive lung adenocarcinoma patients were collected pre-osimertinib and at the time of progression under osimertinib. All patients progressed under treatment with first- or second-generation TKIs and were *EGFR* T790M-positive based on plasma genotyping by ddPCR prior to the initiation of second-line treatment with osimertinib.

The characteristics of 31 evaluable patients enrolled in this study are summarized in Table 1. All patients had adenocarcinoma histology, stage IV disease at diagnosis and were pretreated with EGFR-TKIs. The median time from initial diagnosis of lung adenocarcinoma until start of osimertinib therapy was 22 months (range 4 to 60 months).

In addition to the assessment of SCNAs by shallow whole-genome plasma sequencing, we identified activating *EGFR* mutations and *EGFR* resistance mutations by means of ddPCR. Prior to the initiation of osimertinib therapy, activating *EGFR* mutations were detected in the plasma of 34/43 (79%) patients, the T790M mutation in all 43 patients, and the C797S mutation in none of the patients (Table 2).

### 3.2. Assessment of SCNAs in Plasma Samples

**Figure 1:**
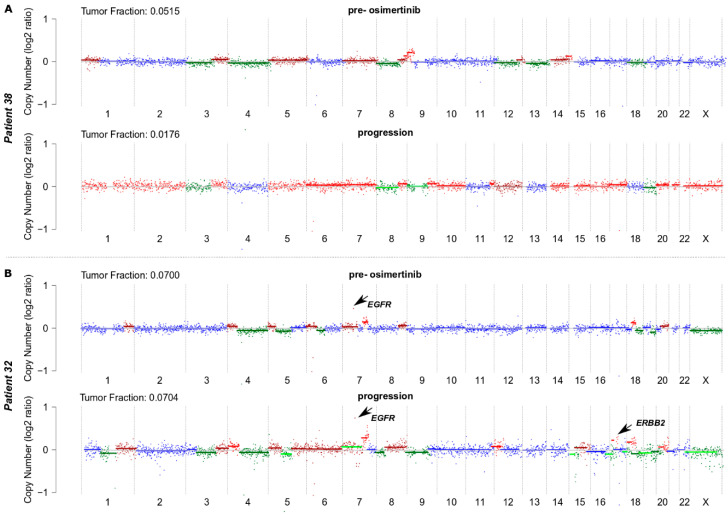
Examples of copy number profiles (pre-osimertinib and at the time of progression) from a patient who responded to osimertinib treatment (**A**) and a non-responding patient (**B**).

We performed shallow whole-genome sequencing of ctDNA to assess SCNAs in plasma samples from each patient collected pre-osimertinib when the T790M mutation was detectable in plasma and clinical progression on EGFR-TKIs was developed, and subsequently at the time of clinical progression on osimertinib (Table 2). Profiles of SCNAs in all 86 samples are shown in Figure S1. In addition, a table of all identified SCNAs is available in the supplement (see Table S1). Examples of copy number profiles from a patient who responded to osimertinib and a non-responding patient are shown in Figure 1.

The median TF calculated with ichorCNA for all evaluable samples (n = 80) was 4.9% (range 3.0–42.6%). There was no difference between the median TF at osimertinib initiation (median 5.1%, range 3.0–42.4%) and at the time of progression (median 4.7%, range 3.2–42.6%) (*p* = 0.79; see Figure S2A). Twenty-one of 39 (54%) pre-osimertinib samples and 18 of 41 (44%) samples at the time of progression under osimertinib had a TF greater than ≥5% (Table 2).

We observed a significant positive correlation between the copy number of the activating *EGFR* mutations and the TF in plasma samples assessed pre-osimertinib (Spearman Rho 0.46, *p* = 0.002) (see Figure S3A) and a trend towards higher TF with increasing T790M copy number assessed pre-osimertinib (Spearman Rho 0.30, *p* = 0.054) (see Figure S3B).

**Figure 2:**
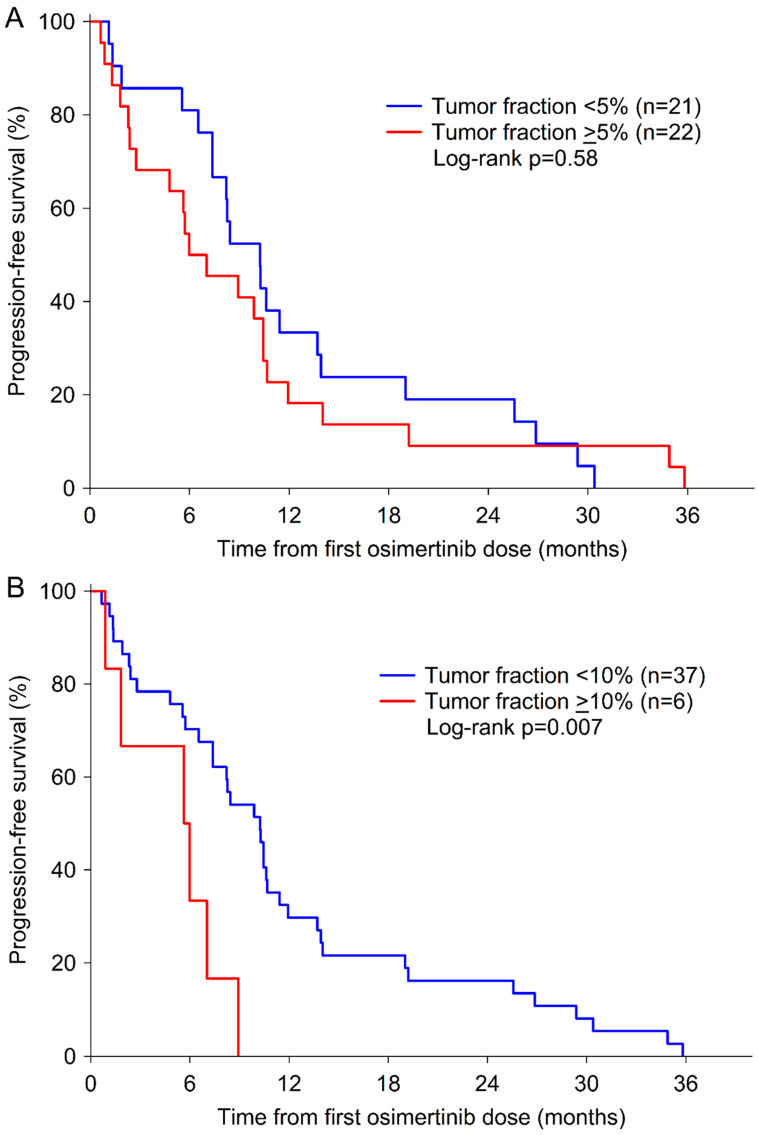

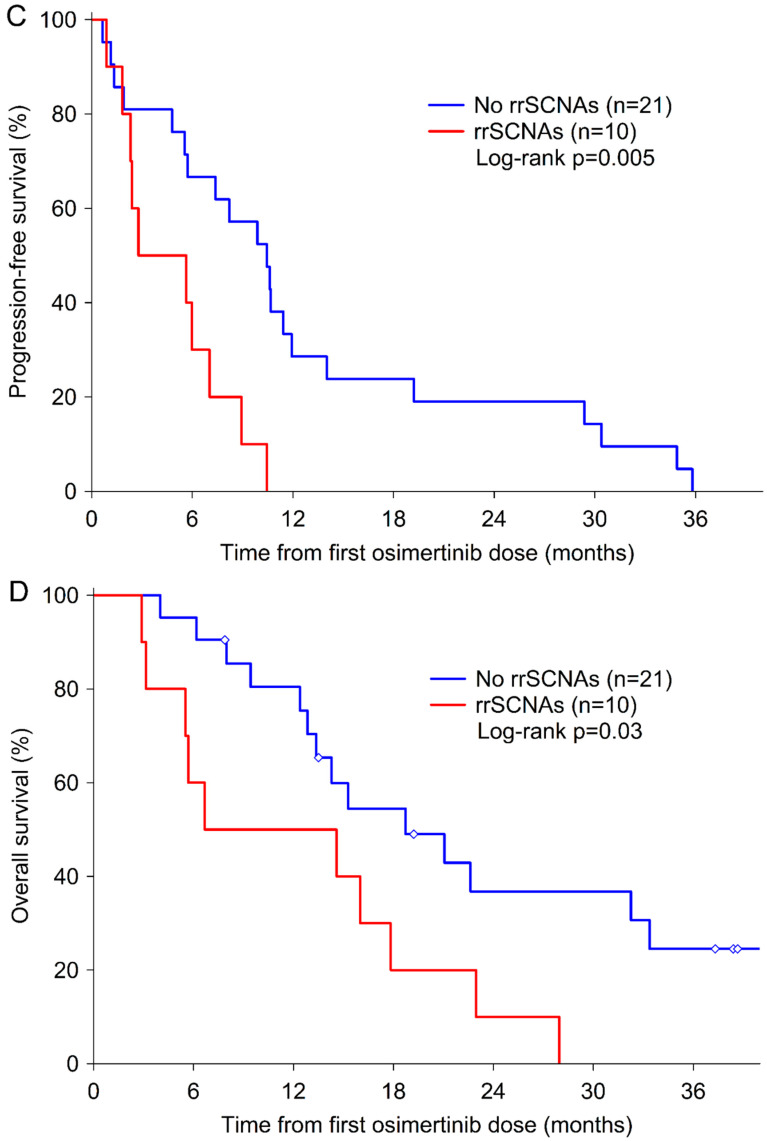
Kaplan–Meier curves of estimated PFS and OS. PFS according to a tumor fraction of 5% (**A**) and 10% (**B**). Patients with detectable rrSCNAs had a significantly shorter PFS (**C**) and OS (**D**) compared to patients without detectable rrSCNAs in plasma ctDNA before the start of osimertinib.

Next, we specifically called for focal SCNA, which often contain clinically relevant genes. These genes were selected based on a literature search. Since ichorCNA-based assessment of copy number calling is based on 1Mb bins and focal genomic amplifications are often narrow, we applied our plasma-Seq-based focal amplification calling algorithm, which is based on a 50 kbp bin approach [32]. Focal amplifications are mostly accompanied with a high copy number leading to a regionally increased TF for the specific region; therefore, these events can be detected with a higher resolution than gross SCNAs (single copy loss/gain) [32]. This revealed focal SCNAs in 44 of 81 (54%) samples, many of which included well characterized driver genes in lung cancer and were previously associated with resistance to osimertinib (rrSCNAs; resistance-related SCNAs). Of all 44 cases with focal SCNA, 25 (57%) had a TF ≥5% and 19 (43%) had a TF <5%. The median TF was higher in samples with focal SCNA than in those without (median 5.6% versus 4.5%, *p* = 0.02) (see Figure S2B). While a 5% cut-off did not have a significant impact on PFS (*p* = 0.58), patients with a TF ≥10% had a significantly shorter PFS compared to patients with a TF <10% (*p* = 0.007) (Figure 2A,B).

We identified rrSCNAs in *EGFR* (n = 6), *ERBB2* (n = 1), *CDK4* (n = 2), *CDK6* (n = 1), *MDM2* (n = 3), *CDKN2A* (n = 1), *AKT2* (n = 1), and *RB1* (n = 1) in pre-osimertinib plasma samples (see Table S2). A simultaneous change in two or more resistance-related genes was observed in four samples (Table 2). rrSCNAs were observed in *EGFR* (n = 3), *ERBB2* (n = 1), *CDK4* (n = 2), *MDM2* (n = 2), *RB1* (n = 1), and *AKT2* (n = 1) which were present in both pre-osimertinib specimens and in samples taken at the time of osimertinib resistance (Table 2). In some patients, rrSCNAs in *EGFR* (n = 2), *ERBB2* (n = 2), *CDK4* (n = 1), *MET* (n = 1), and *PIK3CA* (n = 1) were identified only at the time of progression under osimertinib (Table 2). As the presence of rrSCNAs should be considered in combination with the TF in the plasma sample, the relationship of the TF in samples before osimertinib initiation and at progression under osimertinib is shown in Figure S4.

After progression under osimertinib therapy, the activating *EGFR* mutation observed at the time of diagnosis was detectable in 30/43 patients (70%), T790M in 17/43 patients (40%) and 8/43 (19%) patients developed the C797S mutation (Table 2). In seven patients activating *EGFR* mutations, T790M and C797S were present simultaneously. C797S mutations and rrSCNAs were not mutually exclusive. In three C797S-positive plasma samples, rrSCNAs were also detected in *EGFR* (n = 2), *CDK4*/*CDK6* (n = 2), and *MDM2* (n = 1) (Table 2).

As some samples had borderline tumor fractions around the detection limit of ichorCNA, we analyzed 10 control samples with a similar number of reads to assess the background noise. The median TF of the controls was 1.1% (range 0–4.2) and five of them were called as 0. In contrast, TF of the NSCLC cases ranged from 1–42% with a median of 5% (*p* < 0.001). Profiles of the control samples are shown in Figure S5.

### 3.3. Clinical Relevance of rrSCNAs

The presence of rrSCNAs cannot be completely excluded in 12 cases with a TF <5% and when no SCNAs have been detected (Table 3). Therefore, these patients were excluded from all outcome analyses. We observed no association between pre-osimertinib rrSCNAs and age, gender, absence or presence of extra-thoracic metastases, tissue genotype at diagnosis, and previous EGFR-TKI therapy (Table 1). However, the TF in plasma samples was significantly higher in samples with rrSCNAs (n = 10) compared to those without detectable rrSCNAs (n = 21) (17.0% versus 5.1%, *p* < 0.0001) (Table 1).

The osimertinib response rate was 71% (22 of 31 patients) and the disease control rate (DCR) was 74% (23 of 31 patients). The median TF in plasma DNA samples was not different in patients who responded to osimertinib compared to those who did not respond (5.4% versus 7.0%, *p* = 0.36). Patients without detectable rrSCNAs in pre-osimertinib samples had a better response to osimertinib than patients with detectable rrSCNAs (81% versus 50%, *p* = 0.08) (Table 4).

At a median follow-up time of 38.4 months (95% CI 36.2–40.6), all patients had progressed and 24 of 31 (77%) had died. The median PFS and OS were 7.4 and 15.3 months, respectively. Age, gender, presence or absence of extra-thoracic metastases, previous EGFR-TKI therapy, and TF were not associated with PFS or OS (Table 5). However, patients with exon 18 or L861Q mutations had a shorter PFS and OS (Table 5). The presence of rrSCNAs in pre-osimertinib samples predicted shorter PFS (median 2.8 months versus 10.4 months; HR 3.33, 95% CI 1.37–8.10, *p* = 0.008) (Figure 2C) and OS (median 6.7 months versus 18.7 months; HR 2.54, 95% CI 1.09–5.92, *p* = 0.03) (Figure 2D). Multivariate analyses using stepwise backward elimination models demonstrated that the presence of rrSCNAs was the only significant predictor of shorter PFS and OS after adjusting for clinical parameters (Table 5).

## 4. Discussion

Shallow whole-genome sequencing was recently shown to be useful to characterize the landscape and evolution of SCNAs in plasma ctDNA of prostate cancer and colorectal cancer patients [31,34,35]. In our study, we used shallow whole-genome plasma sequencing to evaluate SCNAs of resistance-related genes in ctDNA of *EGFR*-mutated lung adenocarcinoma patients who had developed the T790M mutation, progressed after treatment with first- or second-generation EGFR TKIs and who were subsequently treated with osimertinib. We detected various rrSCNAs that were described in previous reports to mediate osimertinib resistance. In particular, it has previously been shown that amplifications of wildtype or mutant *EGFR* [36,37,38], *ERBB2* amplifications [39], *MET* amplifications [40], and rrSCNAs of *CDKN2A* or *CDK4/6* [21,40] are associated with rapid progression to osimertinib and acquired drug resistance. In our study, we observed no *MET* amplification in plasma samples prior to osimertinib and only one *MET* amplification at the time of osimertinib resistance, which is in contrast to published frequencies of *MET* amplification. [41]. Notably, the activation of *MET* signaling can also be a result of polysomy of chr7 or gain of chr7q, which could be identified in a variety of other patients, but was not considered as a focal rrSCNA. We observed various patterns of rrSCNAs in plasma samples from each patient. In pattern 1, no rrSCNAs were detected before and after osimertinib. In pattern 2, the same rrSCNAs were present before start of osimertinib treatment and at the time of osimertinib resistance. In pattern 3, rrSCNAs were observed only before start of osimertinib treatment but not at the time of osimertinib resistance. In pattern 4, rrSCNAs were observed only at the time of osimertinib resistance. Moreover, rrSCNAs appear in combination with the osimertinib resistance *EGFR* C797S mutation but were also independent of C797S. Thus, osimertinib resistance mechanisms are more diverse and complex compared to resistance to first- and/or second-generation TKIs which is mainly caused by the T790M mutation. A limitation, however, is the fact that compared to ddPCR, SCNAs can only be detected in samples with elevated tumor fractions (3–5% and higher). A hard threshold cannot be set because the detection of focal SCNAs depends not only on the TF but also on the amplitude of the SCNA. Although molecular profiling from tumor tissue may lead to higher resolution due to a higher TF, re-biopsies are generally difficult to obtain due to their invasive nature and the poor performance status of many advanced NSCLC patients. Another limitation may be that we used a highly selected patient cohort, and our conclusion may not apply to a general NSCLC cohort.

Regarding activating and resistance *EGFR* mutations, we confirmed that the development of the C797S mutation is one of the most common EGFR-dependent resistance mechanisms against osimertinib and that C797S typically occurs simultaneously with the activating *EGFR* mutations and T790M in plasma ctDNA [7,10]. At the time of osimertinib resistance, the T790M mutation was still present in 42% and was undetectable in 58% of the patients, which is in line with other reports [7,40].

The findings of our present study suggest that rrSCNAs in plasma ctDNA detected before second-line therapy with osimertinib are clinically relevant in patients with advanced *EGFR*-mutated lung adenocarcinoma. The presence of rrSCNAs before the start of osimertinib is associated with shorter PFS and OS of these patients. Therefore, the detection of rrSCNAs before starting osimertinib treatment could be helpful for guiding treatment in the future. According to our results, patients in whom no rrSCNAs are detectable should continue with osimertinib alone. However, patients with detectable rrSCNAs in resistance-related genes in plasma ctDNA may need to change treatment due to their poor outcome. They may benefit from adding chemotherapy or other treatments to osimertinib.

This treatment strategy is strengthened by the results of two phase III trials in the first-line setting, in which the combination of gefitinib with chemotherapy resulted in longer PFS in both trials and longer OS in one of these trials compared to gefitinib alone [42,43]. Therefore, the combination of osimertinib with chemotherapy requires further study in clinical trials in patients with detectable rrSCNAs. Other combination therapies with osimertinib are currently under investigation, e.g., the combination of osimertinib with the VEGF-inhibitor bevacizumab (NCT02803203), or with the EGFR inhibitors necitumumab (NCT02496663) or dacomitinib (NCT03810807) have already entered clinical trials.

## 5. Conclusions

Our study contributes to a comprehensive view of the evolution of the tumor genome during the treatment of *EGFR*-mutated lung adenocarcinoma patients. Furthermore, our results indicate that shallow whole-genome plasma sequencing in *EGFR*-mutated lung adenocarcinoma patients provides clinically relevant information. Patients with detectable rrSCNAs in plasma ctDNA before starting osimertinib have shorter survival and may require other treatments such as the combination of osimertinib with chemotherapy, chemo-immunotherapy or other drugs. These treatment options should be explored within clinical trials in the future and may further improve the outcome of patients with advanced *EGFR*-mutated lung adenocarcinoma.

## Figures and Tables

**Table 1:** Characteristics of evaluable patients.

Clinical Characteristics	No. of Patients n = 31	No rrSCNAs n = 21	rrSCNAs n = 10	*p*-Value
Age				0.88
Median (range)—years	66 (48–83)	66 (48–83)	65 (54–77)	
<65 years	13 (42%)	9 (43%)	4 (40%)	
≥65 years	18 (58%)	12 (57%)	6 (60%)	
Sex				0.21
Female	20 (65%)	12 (57%)	8 (80%)	
Male	11 (35%)	9 (43%)	2 (20%)	
Metastases at diagnosis				0.06
M1a	6 (19%)	6 (29%)	0 (0%)	
M1b	25 (81%)	15 (71%)	10 (100%)	
*EGFR* tissue genotype				0.77
Exon 19 deletion	21 (68%)	15 (71%)	6 (60%)	
L858R	8 (26%)	5 (24%)	3 (30%)	
L861Q	2 (7%)	1 (5%)	1 (10%)	
Previous EGFR-TKI therapy				0.82
Afatinib	13 (42%)	10 (48%)	3 (30%)	
Erlotinib	3 (10%)	2 (10%)	1 (10%)	
Gefitinib	10 (32%)	6 (28%)	4 (40%)	
>1 EGFR-TKI	5 (16%)	3 (14%)	2 (20%)	
Tumor fraction				<0.0001
Median (range)	5.7 (1.6–42.4)	5.1 (1.6–8.7)	17.0 (6.9–42.4)	

Percentages may not total 100% because of rounding. rrSCNAs = somatic copy-number alterations in resistance-related genes before osimertinib treatment.

**Table 2:** *EGFR* mutations and resistance-related rrSCNAs in plasma samples.

Patient	Pre-Osimertinib			Osimertinib Resistance		
	EGFR Mutation	rrSCNAs	Tumor Fraction	EGFR Mutation	rrSCNAs	Tumor Fraction
Case 1	L858R, T790M	No rrSCNAs	4.5%	L858R, T790M	MET	7.4%
Case 2	del19, T790M	No rrSCNAs	3.0%	del19, T790M	No rrSCNAs	4.1%
Case 3	T790M	No rrSCNAs	4.5%	-	No rrSCNAs	4.5%
Case 4	del19, T790M	MDM2	7.2%	del19, T790M	No rrSCNAs	5.0%
Case 5	T790M	No rrSCNAs	5.0%	del19, C797S	No rrSCNAs	3.9%
Case 6	del19, T790M	No rrSCNAs	4.1%	-	No rrSCNAs	3.9%
Case 7	T790M	No rrSCNAs	3.6%	del19	No rrSCNAs	5.0%
Case 8	L858R, T790M	CDK4, MDM2	7.6%	L858R, T790M	CDK4, MDM2, ERBB2, PIK3CA	10.1%
Case 9	T790M	No rrSCNAs	<3.0%	-	No rrSCNAs	3.4%
Case 10	del19, T790M	EGFR, CDK4, MDM2	19.4%	del19, T790M, C797S	CDK4, MDM2	3.4%
Case 11	T790M	EGFR	22.5%	del19	EGFR	6.7%
Case 12	del19, T790M	No rrSCNAs	5.6%	del19, T790M, C797S	EGFR, CDK4	5.9%
Case 13	del19, T790M	No rrSCNAs	5.3%	del19	No rrSCNAs	10.2%
Case 14	del19, T790M	No rrSCNAs	4.0%	del19, T790M, C797S	No rrSCNAs	3.3%
Case 15	T790M	No rrSCNAs	3.1%	-	No rrSCNAs	3.3%
Case 16	L858R, T790M	ERBB2, CDKN2A	42.4%	L858R, T790M	ERBB2	23.7%
Case 17	L858R, T790M	No rrSCNAs	4.8%	L858R	No rrSCNAs	4.1%
Case 18	del19, T790M	No rrSCNAs	4.9%	-	No rrSCNAs	4.8%
Case 19	T790M	No rrSCNAs	8.7%	-	No rrSCNAs	9.9%
Case 20	del19, T790M	No rrSCNAs	4.9%	del19	No rrSCNAs	4.7%
Case 21	del19, T790M	No rrSCNAs	5.1%	del19	No rrSCNAs	4.9%
Case 22	del19, T790M	No rrSCNAs	3.7%	-	No rrSCNAs	3.2%
Case 23	del19, T790M	EGFR	21.0%	del19, T790M, C797S	EGFR	15.3%
Case 24	T790M	No rrSCNAs	3.3%	-	No rrSCNAs	4.5%
Case 25	del19, T790M	No rrSCNAs	<3.0%	del19, T790M	No rrSCNAs	15.5%
Case 26	del19, T790M	AKT2, RB1	23.8%	T790M	AKT2, RB1	42.6%
Case 27	L858R, T790M	No rrSCNAs	3.2%	L858R, T790M, C797S	No rrSCNAs	3.7%
Case 28	L858R, T790M	No rrSCNAs	7.5%	L858R, T790M, C797S	No rrSCNAs	4.4%
Case 29	L858R, T790M	EGFR, CDK6	14.6%	L858R, T790M	No rrSCNAs	5.3%
Case 30	del19, T790M	EGFR	6.9%	del19	No rrSCNAs	30.8%
Case 31	L858R, T790M	No rrSCNAs	3.0%	L858R	No rrSCNAs	4.1%
Case 32	L861Q, T790M	EGFR	7.0%	L861Q	EGFR, ERBB2	7.0%
Case 33	del19, T790M	No rrSCNAs	6.2%	del19, T790M, C797S	No rrSCNAs	4.3%
Case 34	L858R, T790M	No rrSCNAs	3.9%	-	No rrSCNAs	4.0%
Case 35	T790M	No rrSCNAs	5.7%	-	No rrSCNAs	4.2%
Case 36	del19, T790M	No rrSCNAs	5.9%	-	No rrSCNAs	4.0%
Case 37	del19, T790M	No rrSCNAs	3.9%	del19	No rrSCNAs	7.8%
Case 38	del19, T790M	No rrSCNAs	5.1%	del19	No rrSCNAs	<3.0%
Case 39	L858R, T790M	No rrSCNAs	3.8%	L858R	No rrSCNAs	<3.0%
Case 40	L861Q, T790M	No rrSCNAs	7.9%	L861Q	EGFR	4.5%
Case 41	del19, T790M	No rrSCNAs	4.1%	del19, T790M	No rrSCNAs	4.5%
Case 42	del19, T790M	No rrSCNAs	<3.0%	-	CDKN2A	8.3%
Case 43	del19, T790M	No rrSCNAs	5.1%	del19, T790M	No rrSCNAs	5.0%

**Table 3:** Copy number of *EGFR* mutations, tumor fraction and SCNAs before the start of osimertinib therapy.

Patient	*EGFR*-Activating Mutation (copies/mL)	*EGFR* T790M (copies/mL)	Tumor Fraction (ichorCNA)	SCNAs **
Case 1	6.7	6.9	4.5%	Yes
Case 2 *	73.1	50.4	3.0%	No
Case 3 *	0	5.2	4.5%	No
Case 4	179.2	86.0	7.2%	Yes
Case 5	0	2.9	5.0%	No
Case 6 *	173.9	10.1	4.1%	No
Case 7 *	0	6.4	3.6%	No
Case 8	166.3	38.1	7.6%	Yes
Case 9 *	0	1.6	<3.0%	No
Case 10	33,559.8	38,092.5	19.4%	Yes
Case 11	0	2.1	22.5%	Yes
Case 12	20.9	14.5	5.6%	No
Case 13	5.1	2.5	5.3%	Yes
Case 14	710.1	124.6	4.0%	Yes
Case 15	0	2.3	3.1%	Yes
Case 16	21,119.3	7.9	42.4%	Yes
Case 17 *	7.6	52.0	4.8%	No
Case 18	2.7	2.3	4.9%	Yes
Case 19	0	7.9	8.7%	No
Case 20 *	122.6	1.9	4.9%	No
Case 21	8.4	5.2	5.1%	No
Case 22 *	763.6	10.2	3.7%	No
Case 23	5010.5	3354.0	21.0%	Yes
Case 24 *	0	1.9	3.3%	No
Case 25	156.8	52.4	<3.0%	Yes
Case 26	1571.9	254.5	23.8%	Yes
Case 27 *	341.6	111.9	3.2%	No
Case 28	966.8	261.2	7.5%	No
Case 29	3891.9	444.3	14.6%	Yes
Case 30	4217.9	649.8	6.9%	Yes
Case 31	125.3	17.0	3.0%	Yes
Case 32	12660.3	1.8	7.0%	Yes
Case 33	201.6	39.2	6.2%	Yes
Case 34 *	5.5	1.8	3.9%	No
Case 35	0	75.8	5.7%	No
Case 36	164.1	52.0	5.9%	No
Case 37	29.3	9.6	3.9%	Yes
Case 38	373.1	91.1	5.1%	No
Case 39	38.0	9.0	3.8%	Yes
Case 40	8373.2	6.0	7.9%	No
Case 41	231.1	93.8	4.1%	Yes
Case 42 *	1.1	1.9	<3.0%	No
Case 43	1540.7	379.8	5.1%	No

* These patients were excluded from all outcome analyses because TF was <5% and no SCNAs were detected and, therefore, the presence of rrSCNAs cannot be completely excluded. ** Details of all identified SCNAs are shown in Table S2**.**

**Table 4:** Osimertinib response according to rrSCNAs in plasma ctDNA of pre-osimertinib treatment samples.

	No rrSCNAs	rrSCNAs	*p*-Value
Complete/Partial Response	17 (81%)	5 (50%)	0.08
Stable/Progressive Disease	4 (19%)	5 (50%)	

**Table 5:** Univariate and multivariate Cox proportional hazards models.

	Progression-Free Survival		Overall Survival	
	Univariate	Multivariate **	Univariate	Multivariate **
	HR * (95% CI); *p* Value	HR (95% CI); *p* Value	HR (95% CI); *p* Value	HR (95% CI); *p* Value
Age	0.99 (0.95–1.04); 0.74	-	0.99 (0.94–1.03); 0.58	-
Gender	1.58 (0.74–3.40); 0.24	-	1.51 (0.64–3.53); 0.35	-
Metastases	1.09 (0.44–2.71); 0.86	-	1.58 (0.54–4.65); 0.41	-
*EGFR* tissue genotype	2.19 (1.29–3.72); 0.004	-	2.49 (1.44–4.31); 0.001	-
Previous EGFR-TKI therapy	1.06 (0.77–1.45); 0.74	-	1.16 (0.81–1.65); 0.43	-
Tumor fraction	1.03 (0.99–1.07); 0.13	-	1.02 (0.98–1.06);0.38	-
rrSCNAs	3.33 (1.37–8.10); 0.008	3.33 (1.37–8.10); 0.008	2.54 (1.09–5.92); 0.03	2.54 (1.09–5.92); 0.03

* HR = hazard ratio; 95% CI = 95% confidence interval; rrSCNAs = somatic copy-number alterations in resistance-related genes. ** Stepwise backward elimination model.

## Data Availability

All data generated or analyzed during this study are included in this published article (and its supplementary information files). Shallow whole genome sequencing data have been deposited at the European Genome-phenome Archive (EGA; http://www.ebi.ac.uk/ega/), which is hosted by the EBI, under the accession number EGAS00001004539.

## Supplementary Materials

The following are available online at https://www.mdpi.com/article/10.3390/biom11050618/s1: Figures S1–S5: Supplementary Figures, Tables S1 and S2: Supplementary Tables. Figure S1. Shallow whole-genome plasma sequencing profiles before initiation of osimertinib and at the time of progression to osimertinib. Figure S2. Tumor fraction in plasma DNA samples collected before initiation of osimertinib and at the time of progression to osimertinib (A) and in samples with or without SCNAs (B). Figure S3. Correlation between the log_10_-transformed copy number of the activating *EGFR* mutations (A), T790M (B) and the TF in plasma samples. Figure S4. Relationship of the tumor fraction in plasma samples (n = 13) with detectable rrSCNAs before start of osimertinib therapy and at the time of progression to osimertinib. Figure S5. Shallow whole-genome plasma sequencing profiles of control samples. Table S1. SCNAs detected by shallow whole-genome plasma sequencing. Table S2. SCNAs in resistance-related genes detected by shallow whole-genome sequencing.
