# Supplementary material for: Somatic Copy-Number Alterations in Plasma Circulating Tumor DNA from Advanced EGFR-Mutated Lung Adenocarcinoma Patients
Source: Biomolecules. 2021 Apr 21;11(5):618. doi: 10.3390/biom11050618 (PMC8143372; doi:10.3390/biom11050618)
Supplement: Supplementary file 1 [file biomolecules-11-00618-s001.zip › Biomolecules 2021_Figure S1.pdf]

Figure S1. Shallow whole-genome plasma sequencing profiles before initiation of osimertinib and at the time of progression to osimertinib. TF Tumor fraction

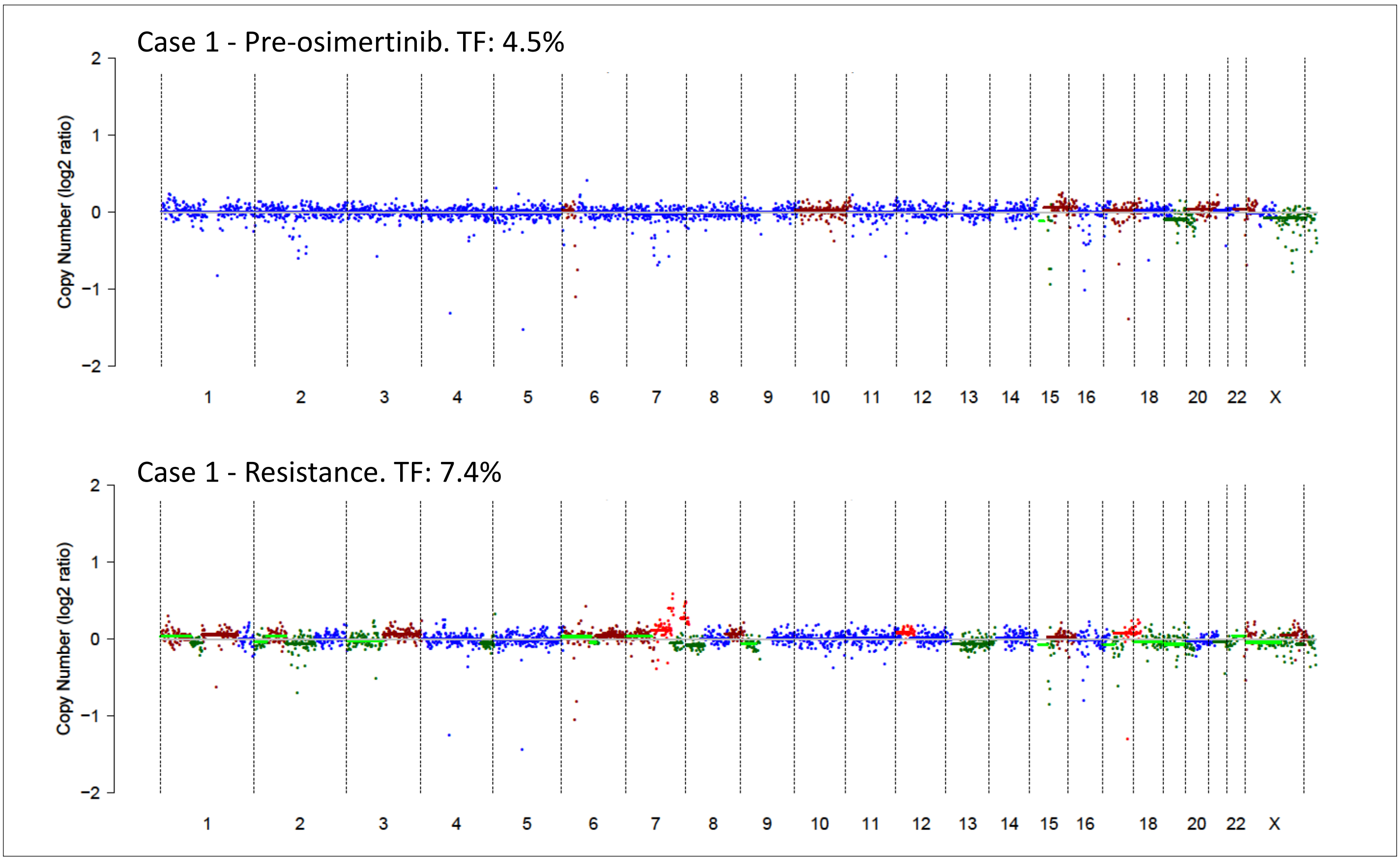

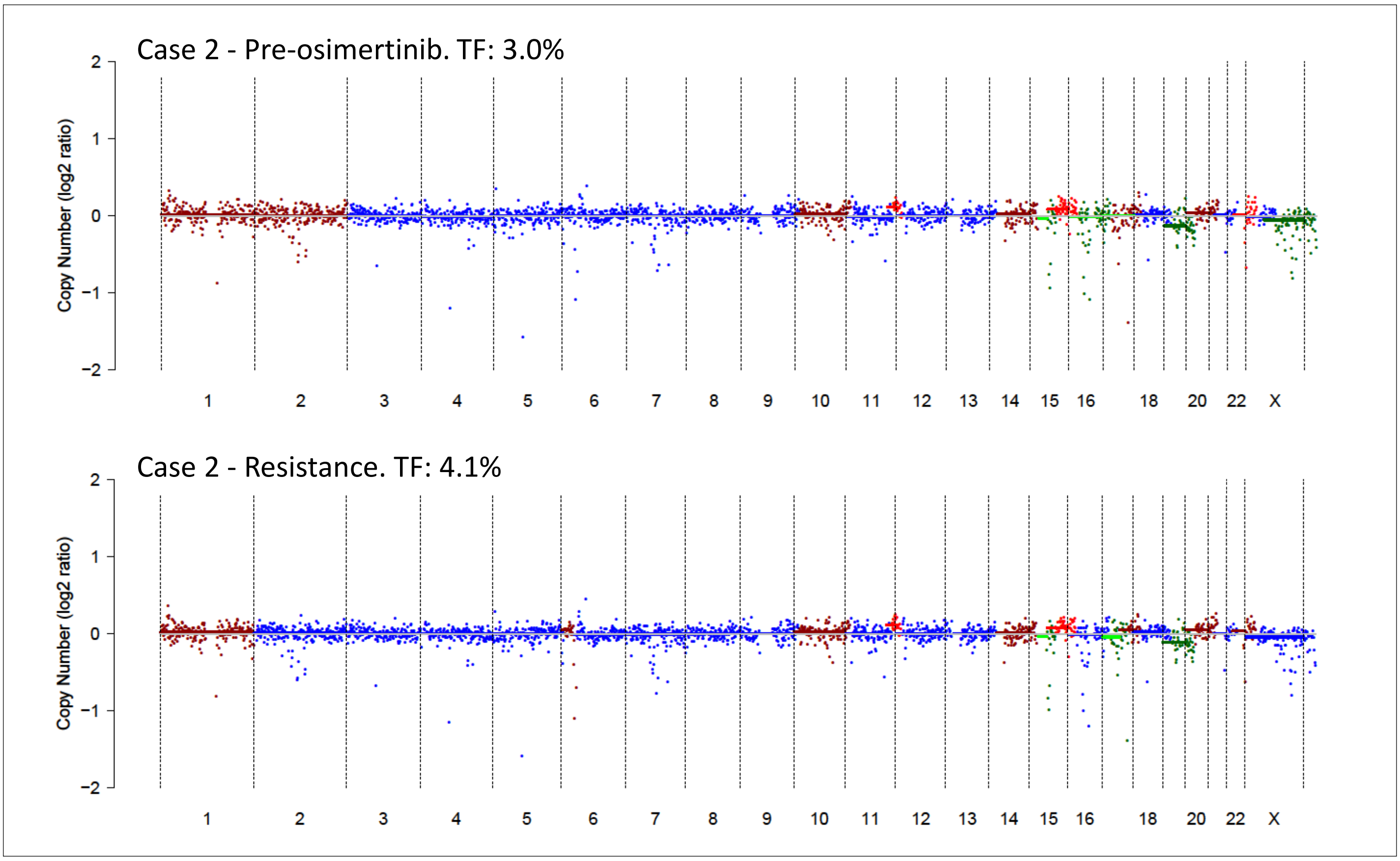

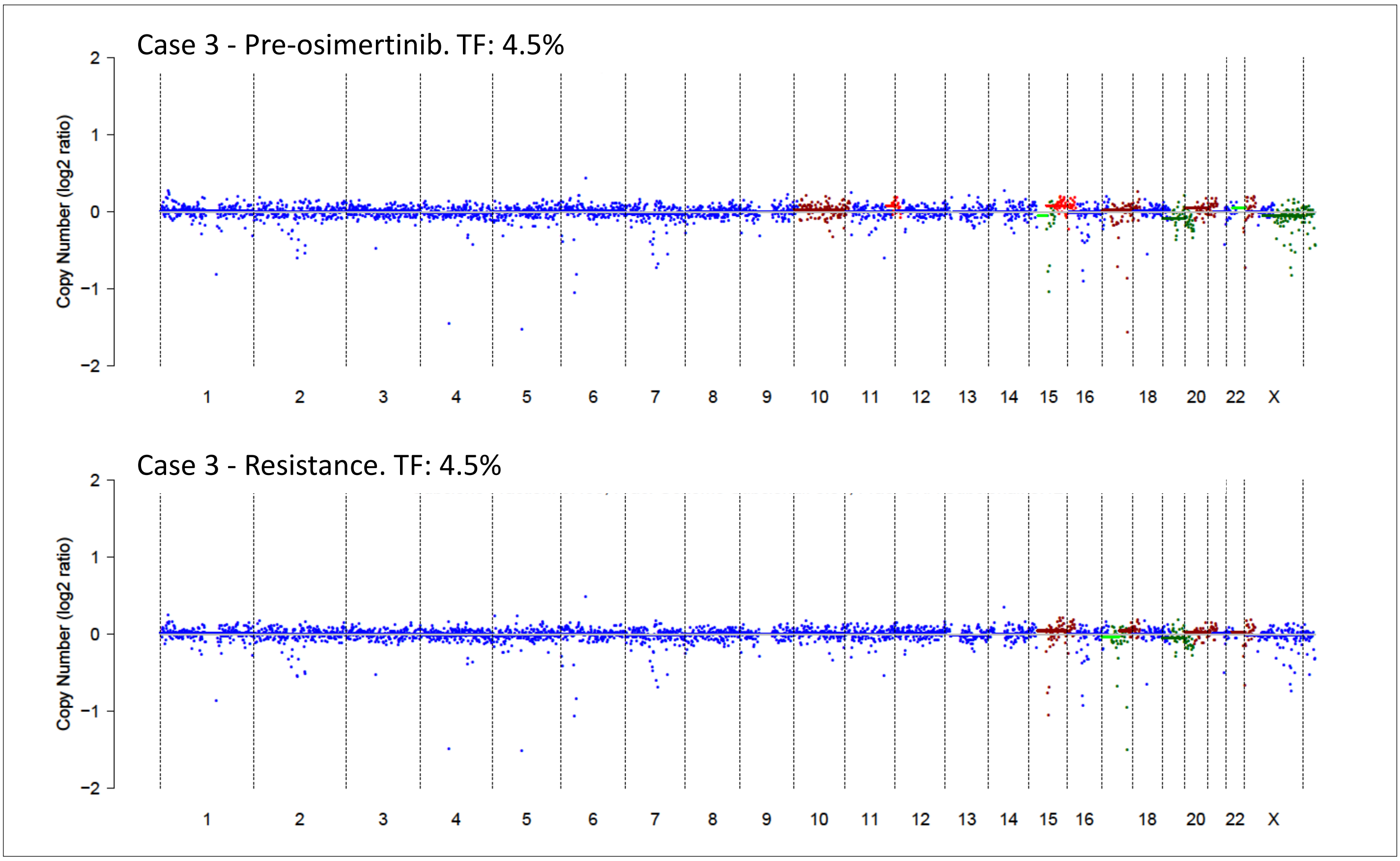
Case 3 - Pre-osimertinib. TF: 4.5%
Case 3 - Resistance. TF: 4.5%
Copy Number (log2 ratio)
2
1
0
−1
−2
1
2
3
4
5
6
7
8
9
10
11
12
13
14
15
16
18
20
22
X

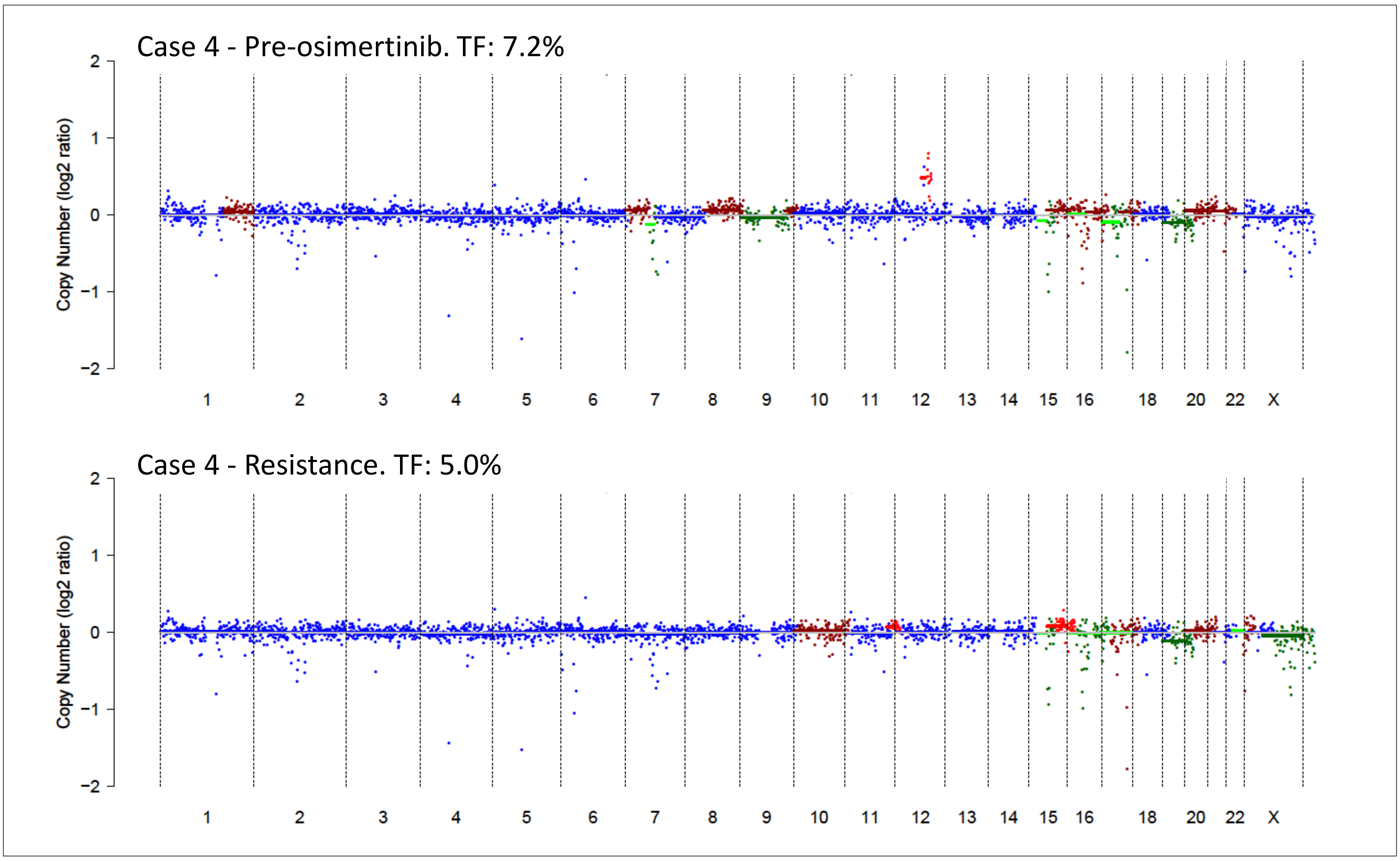
Case 4 - Pre-osimertinib. TF: 7.2%
Case 4 - Resistance. TF: 5.0%
Copy Number (log2 ratio)
2
1
0
−1
−2
1
2
3
4
5
6
7
8
9
10
11
12
13
14
15
16
18
20
22
X

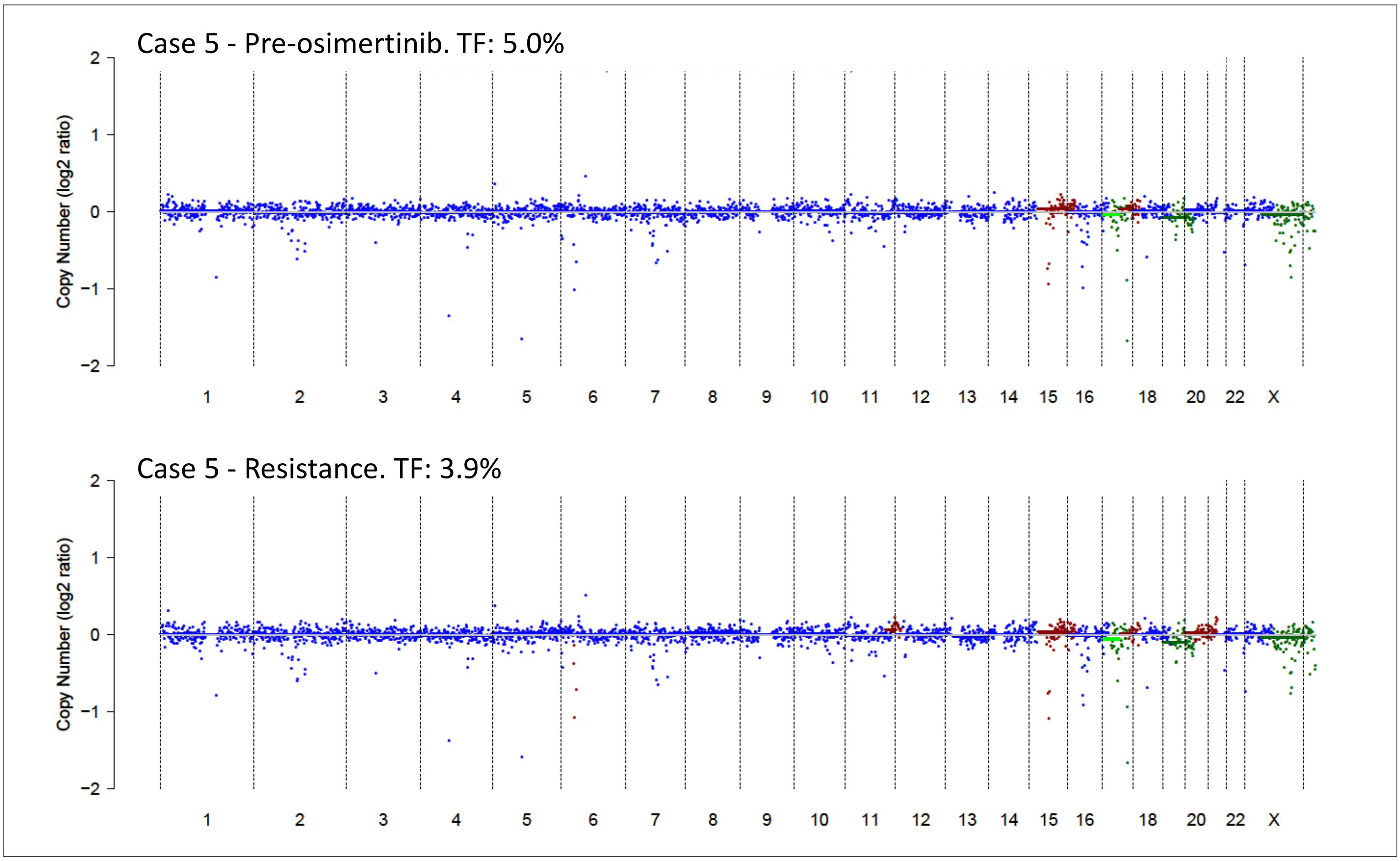
Case 5 - Pre-osimertinib. TF: 5.0%
Copy Number (log2 ratio)
2
1
0
−1
−2
1 2 3 4 5 6 7 8 9 10 11 12 13 14 15 16 18 20 22 X
Case 5 - Resistance. TF: 3.9%
Copy Number (log2 ratio)
2
1
0
−1
−2
1 2 3 4 5 6 7 8 9 10 11 12 13 14 15 16 18 20 22 X

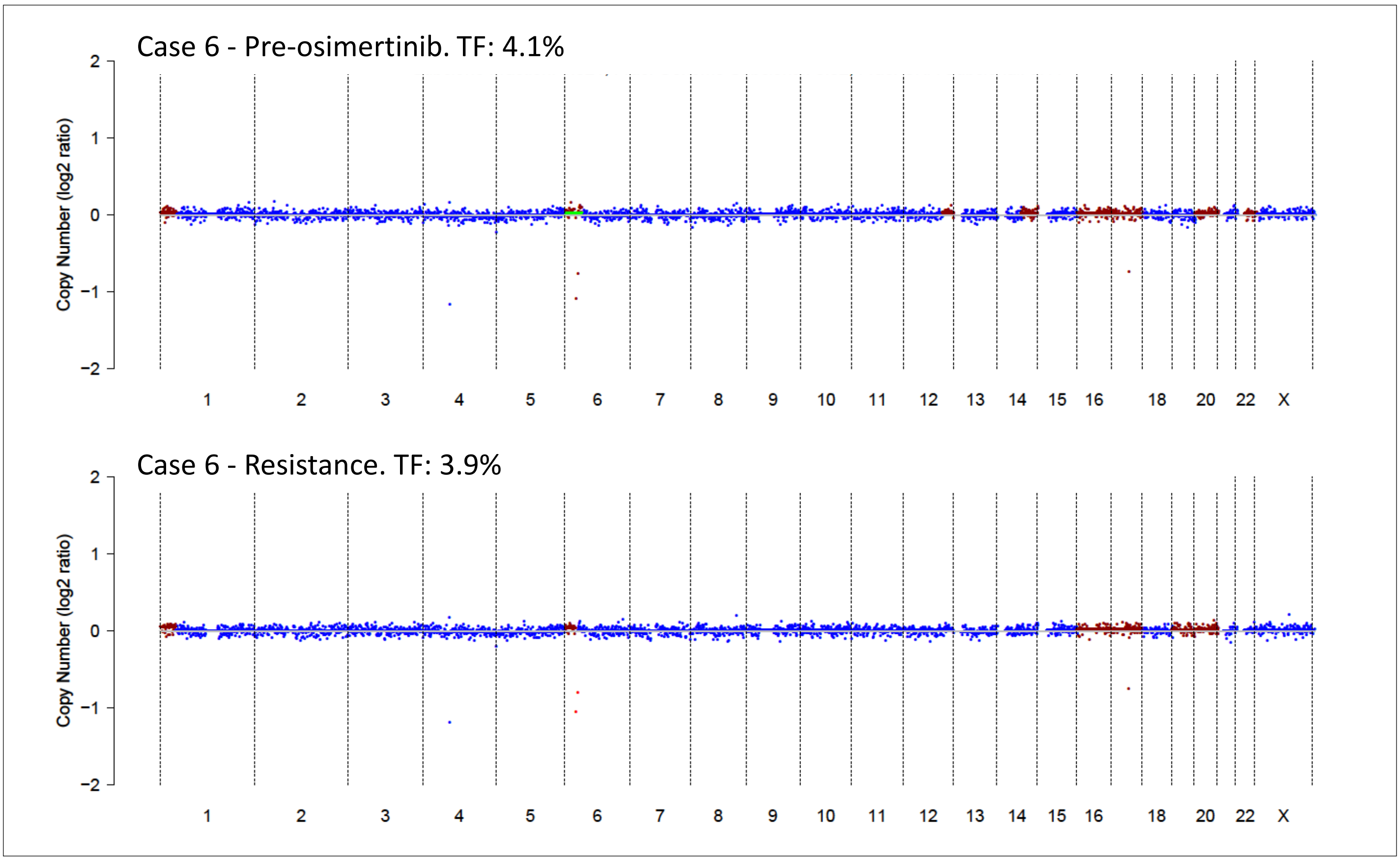
Case 6 - Pre-osimertinib. TF: 4.1%
Copy Number (log2 ratio)
2
1
0
−1
−2
1 2 3 4 5 6 7 8 9 10 11 12 13 14 15 16 18 20 22 X
Case 6 - Resistance. TF: 3.9%
Copy Number (log2 ratio)
2
1
0
−1
−2
1 2 3 4 5 6 7 8 9 10 11 12 13 14 15 16 18 20 22 X

Case 7 - Pre-osimertinib. TF: 3.6%

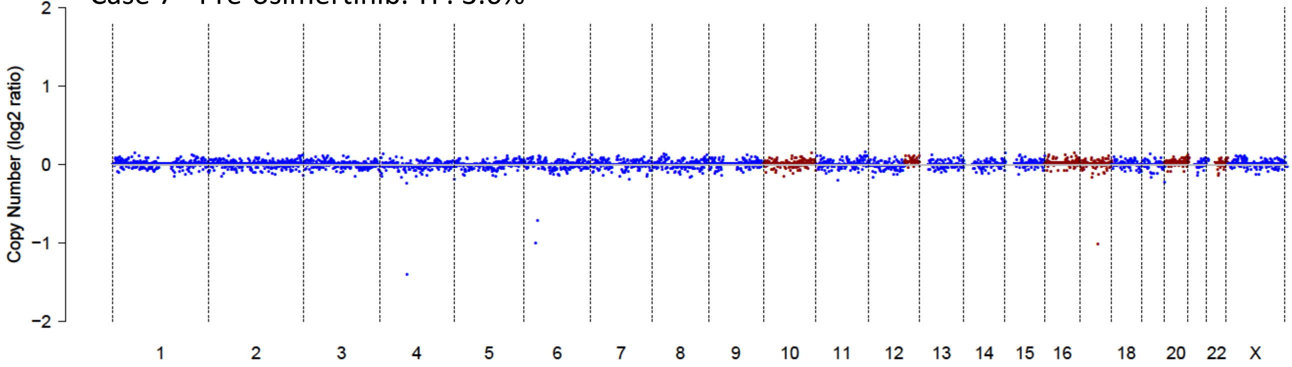

Case 7 - Resistance. TF: 5.0%

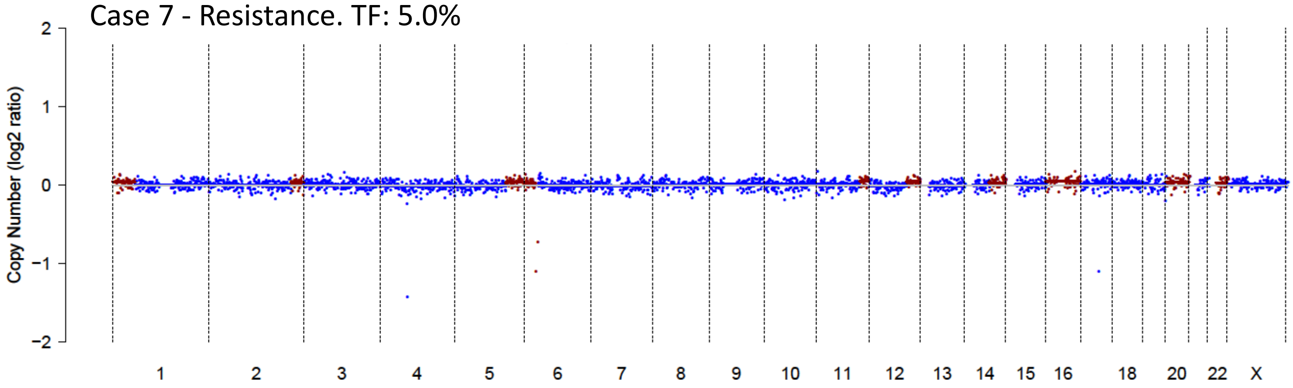

Case 8 - Pre-osimertinib. TF: 7.6%

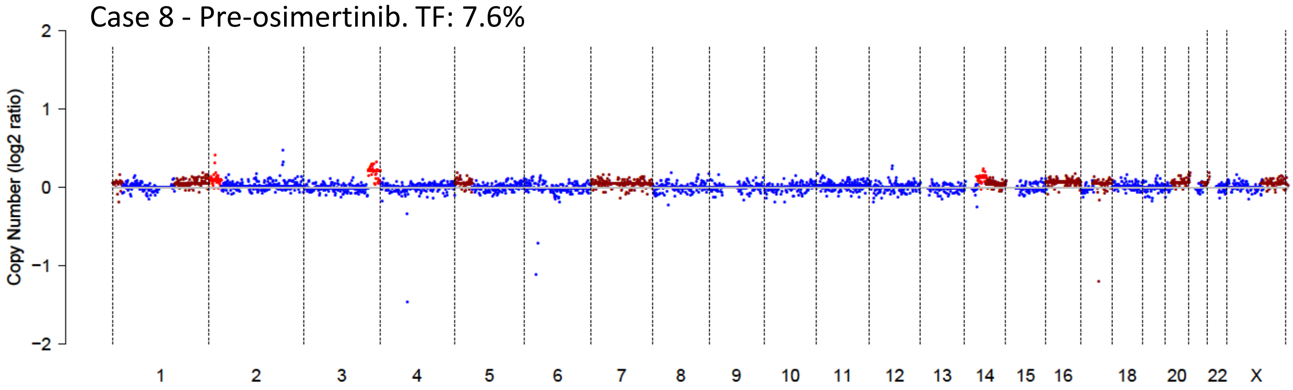

Case 8 - Resistance. TF: 10.1%

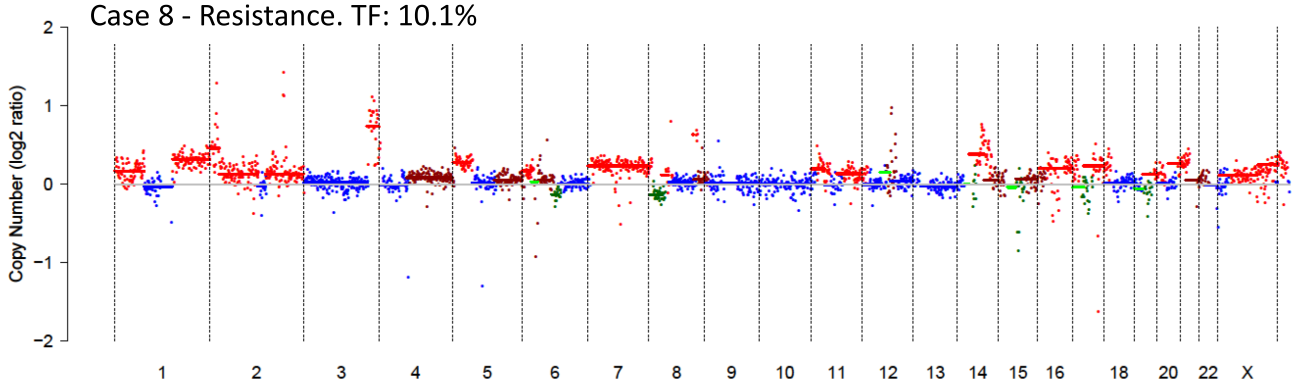

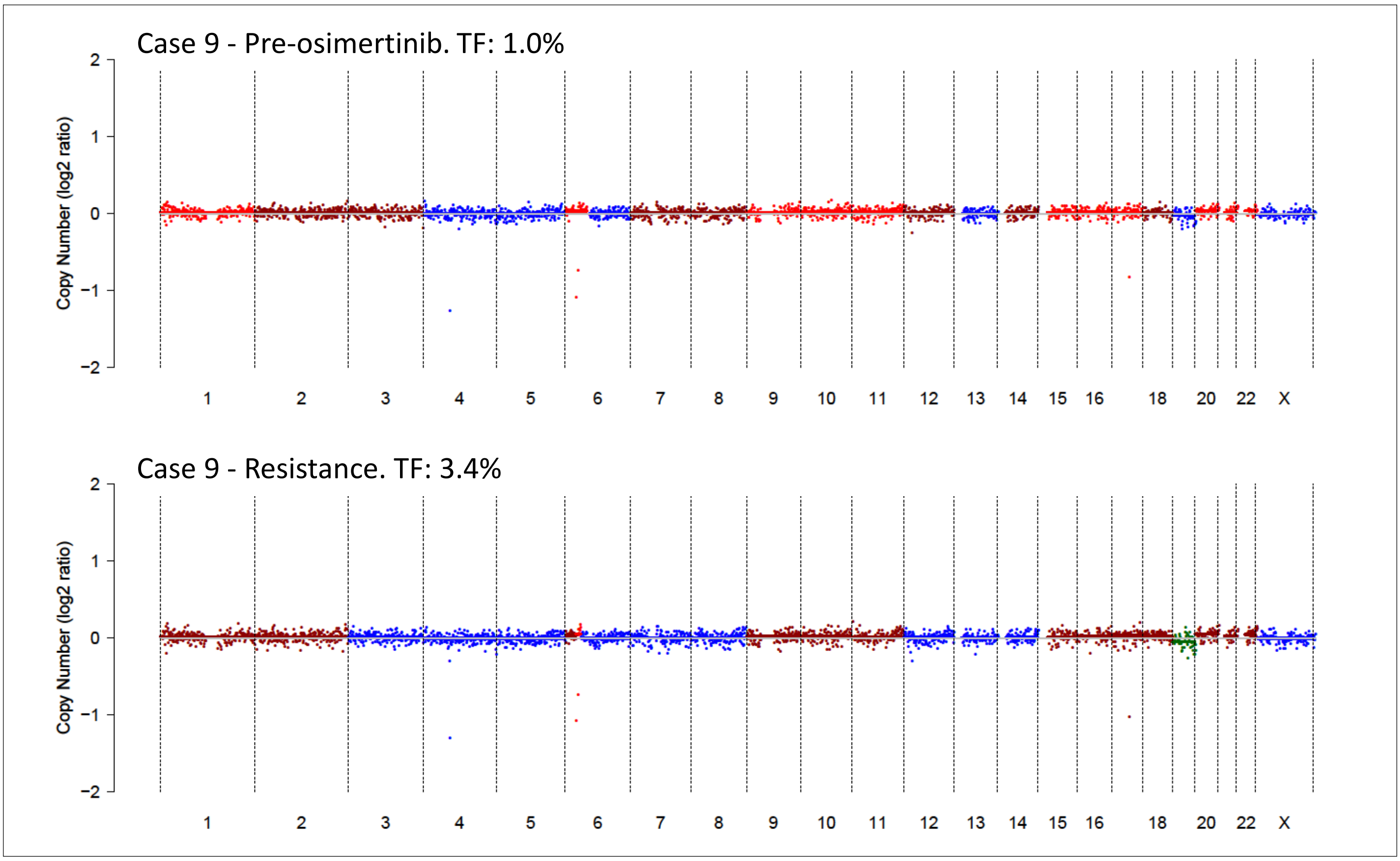
Case 9 - Pre-osimertinib. TF: 1.0%
Case 9 - Resistance. TF: 3.4%
Copy Number (log2 ratio)
2
1
0
−1
−2
1 2 3 4 5 6 7 8 9 10 11 12 13 14 15 16 18 20 22 X

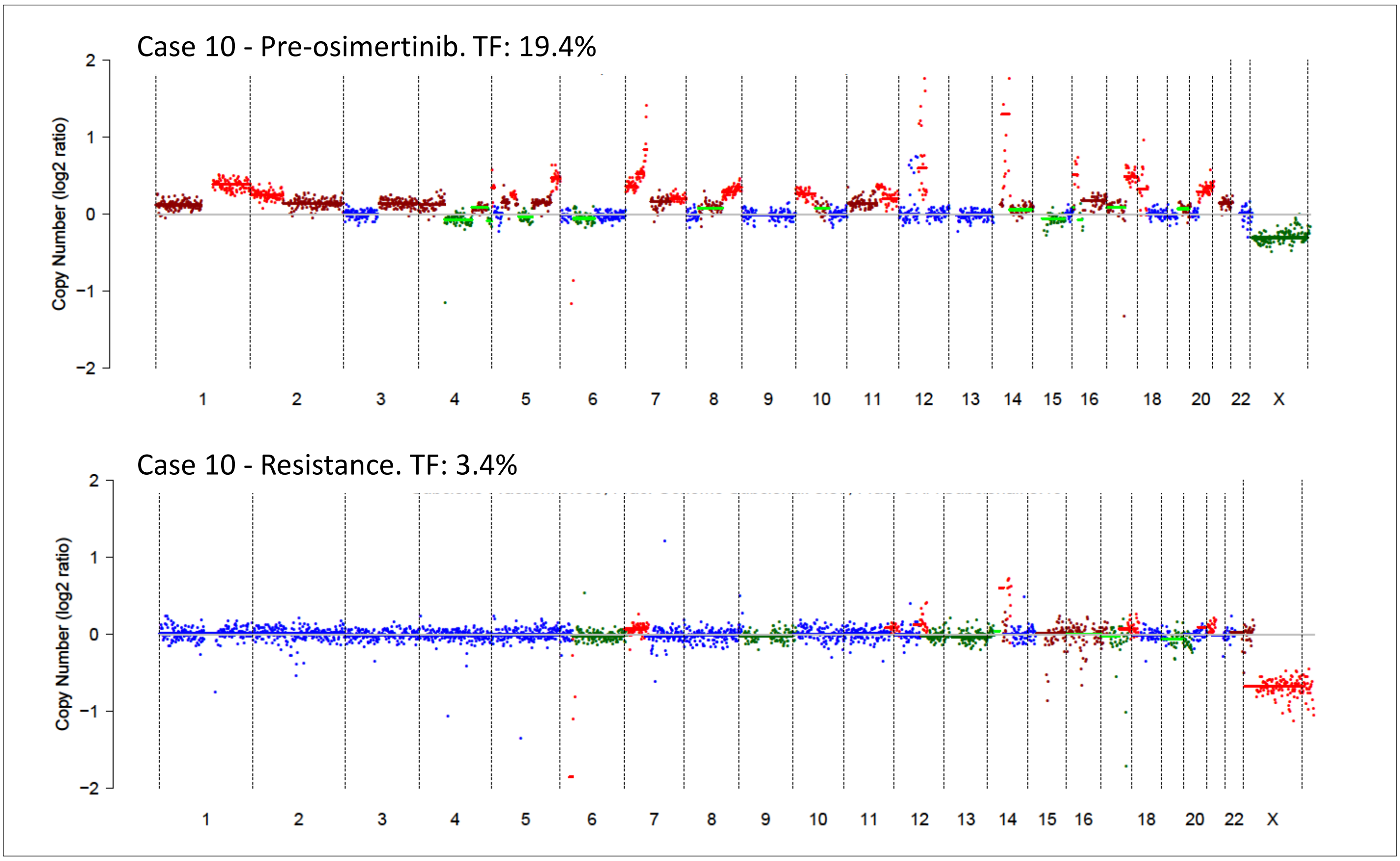
Case 10 - Pre-osimertinib. TF: 19.4%
Case 10 - Resistance. TF: 3.4%
Copy Number (log2 ratio)
2
1
0
−1
−2
1 2 3 4 5 6 7 8 9 10 11 12 13 14 15 16 18 20 22 X

Case 11 - Pre-osimertinib. TF: 22.5%

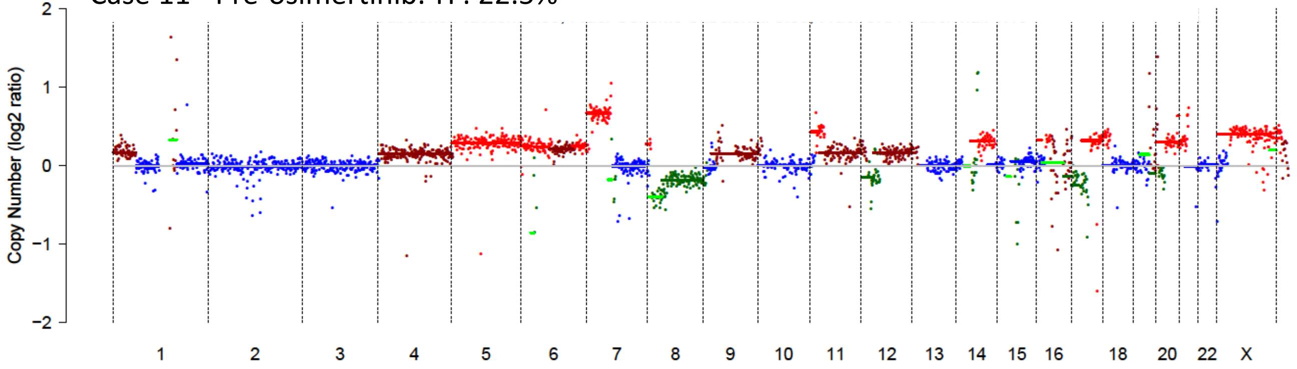

Case 11 - Resistance. TF: 6.7%

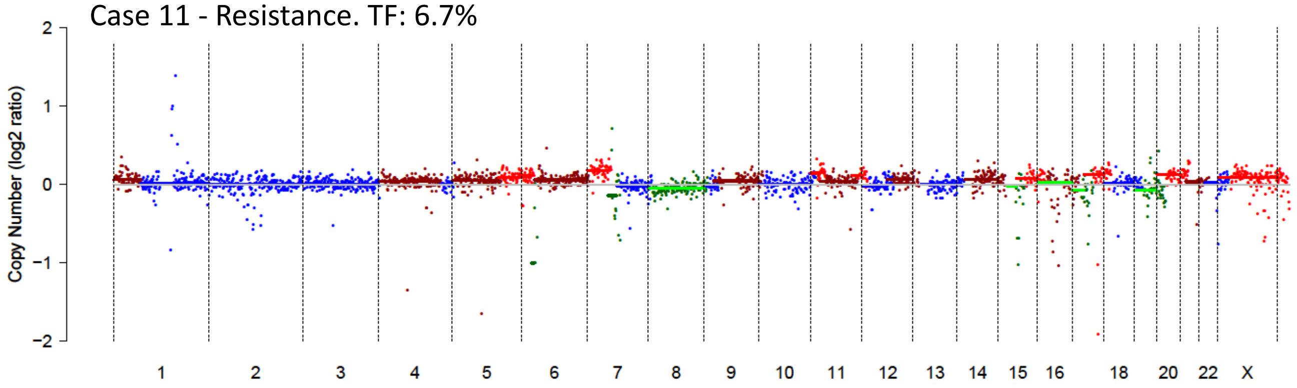

Case 12 - Pre-osimertinib. TF: 5.6%

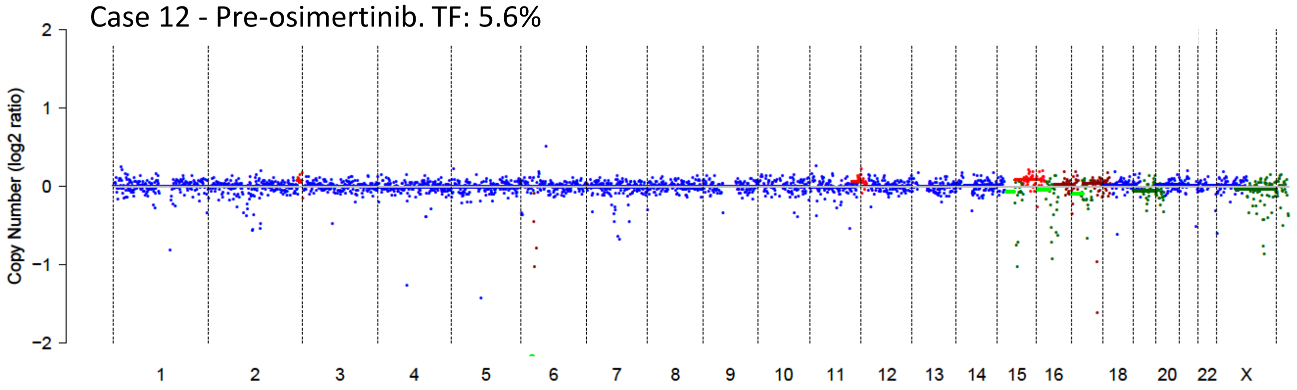

Case 12 - Resistance. TF: 5.9%

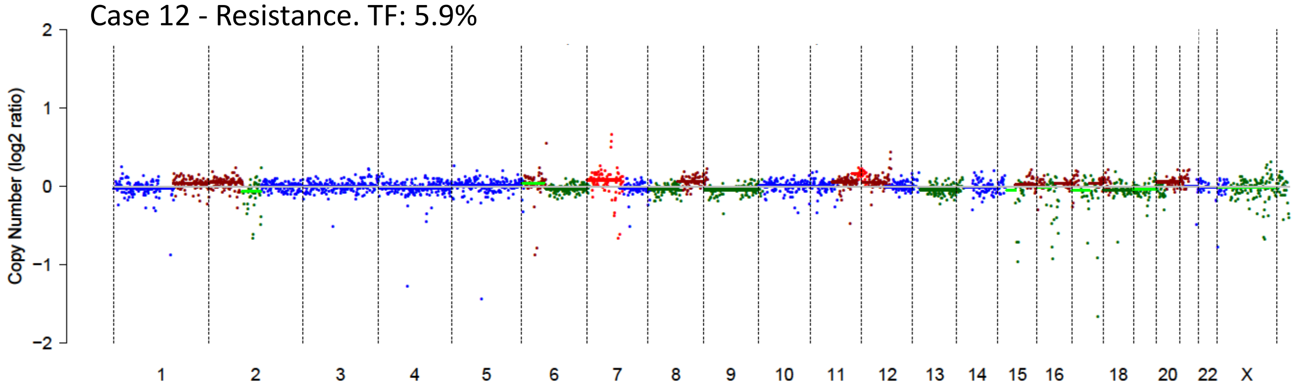

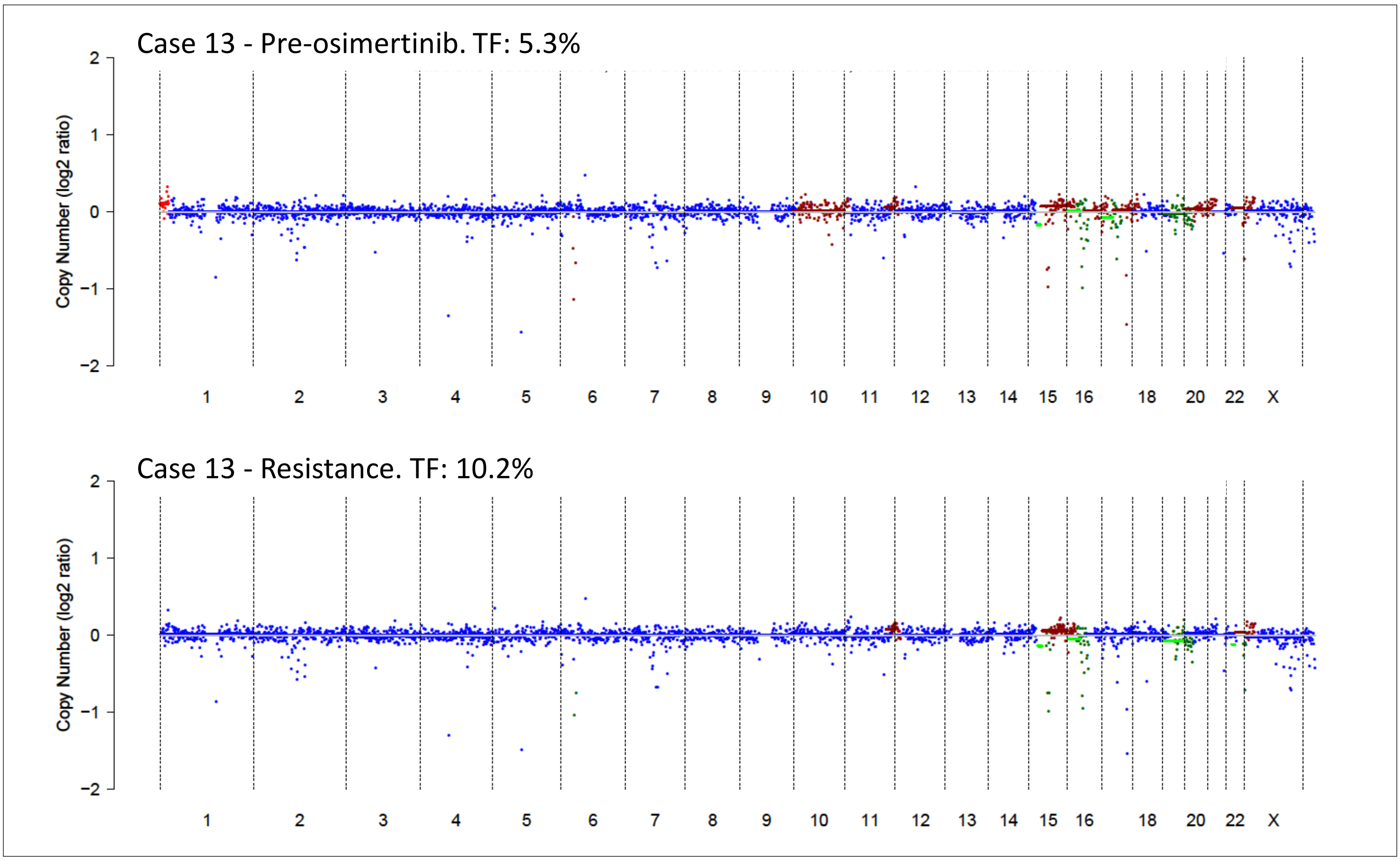

Case 13 - Pre-osimertinib. TF: 5.3%
Case 13 - Resistance. TF: 10.2%
Copy Number (log2 ratio)
2
1
0
−1
−2
1 2 3 4 5 6 7 8 9 10 11 12 13 14 15 16 18 20 22 X

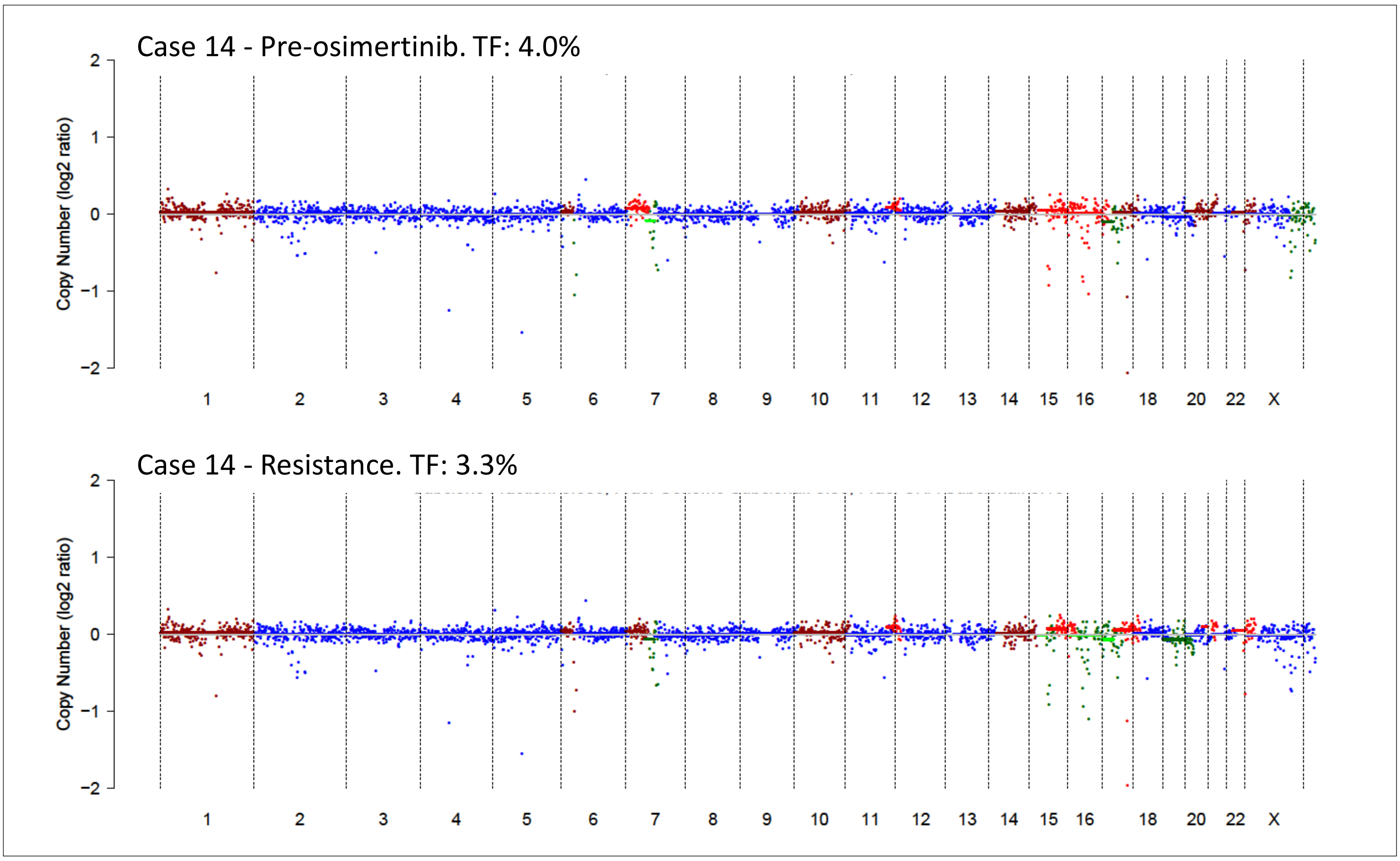

Case 14 - Pre-osimertinib. TF: 4.0%
Case 14 - Resistance. TF: 3.3%
Copy Number (log2 ratio)
2
1
0
−1
−2
1 2 3 4 5 6 7 8 9 10 11 12 13 14 15 16 18 20 22 X

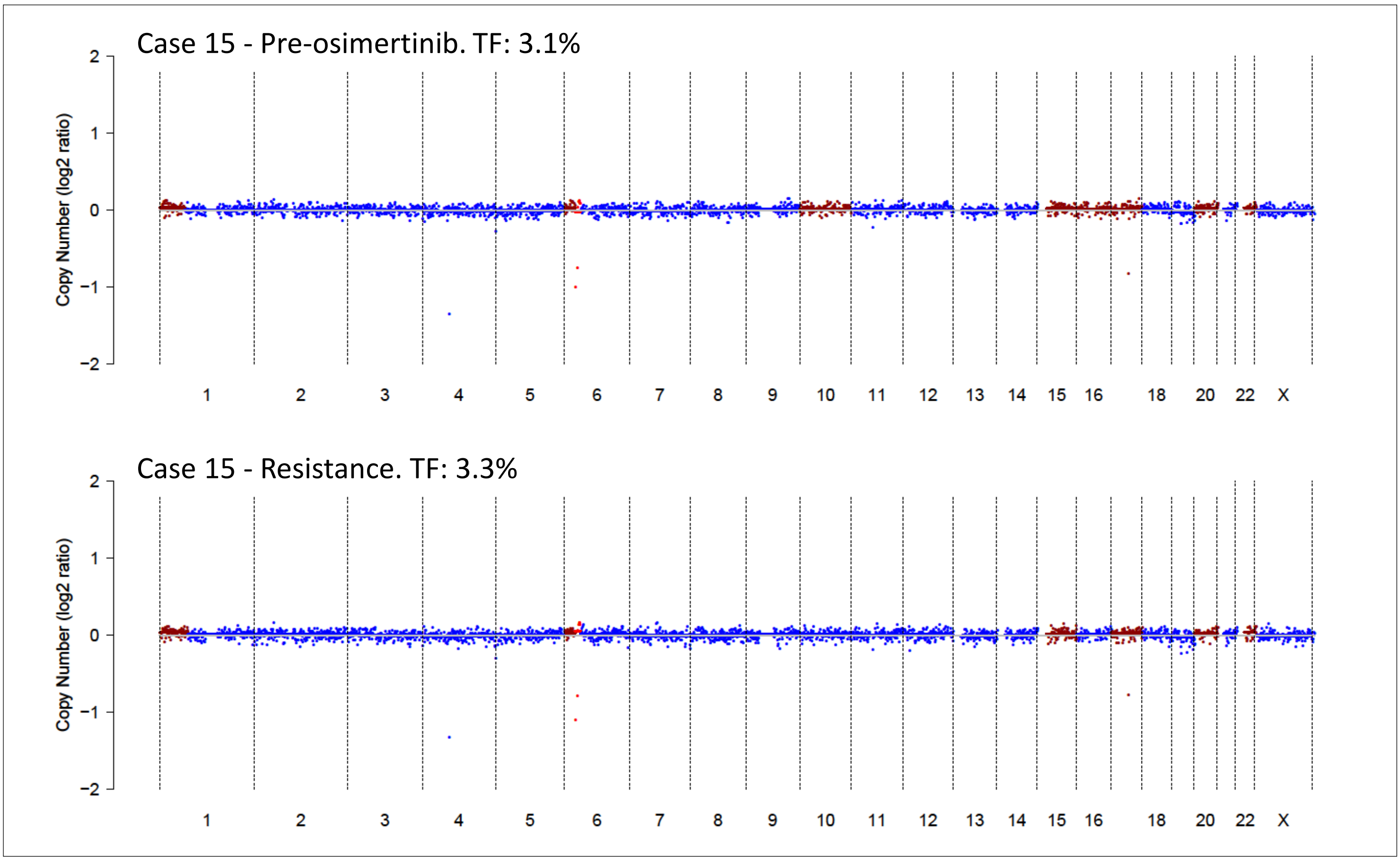
Case 15 - Pre-osimertinib. TF: 3.1%
Case 15 - Resistance. TF: 3.3%
Copy Number (log2 ratio)
2
1
0
−1
−2
1 2 3 4 5 6 7 8 9 10 11 12 13 14 15 16 18 20 22 X

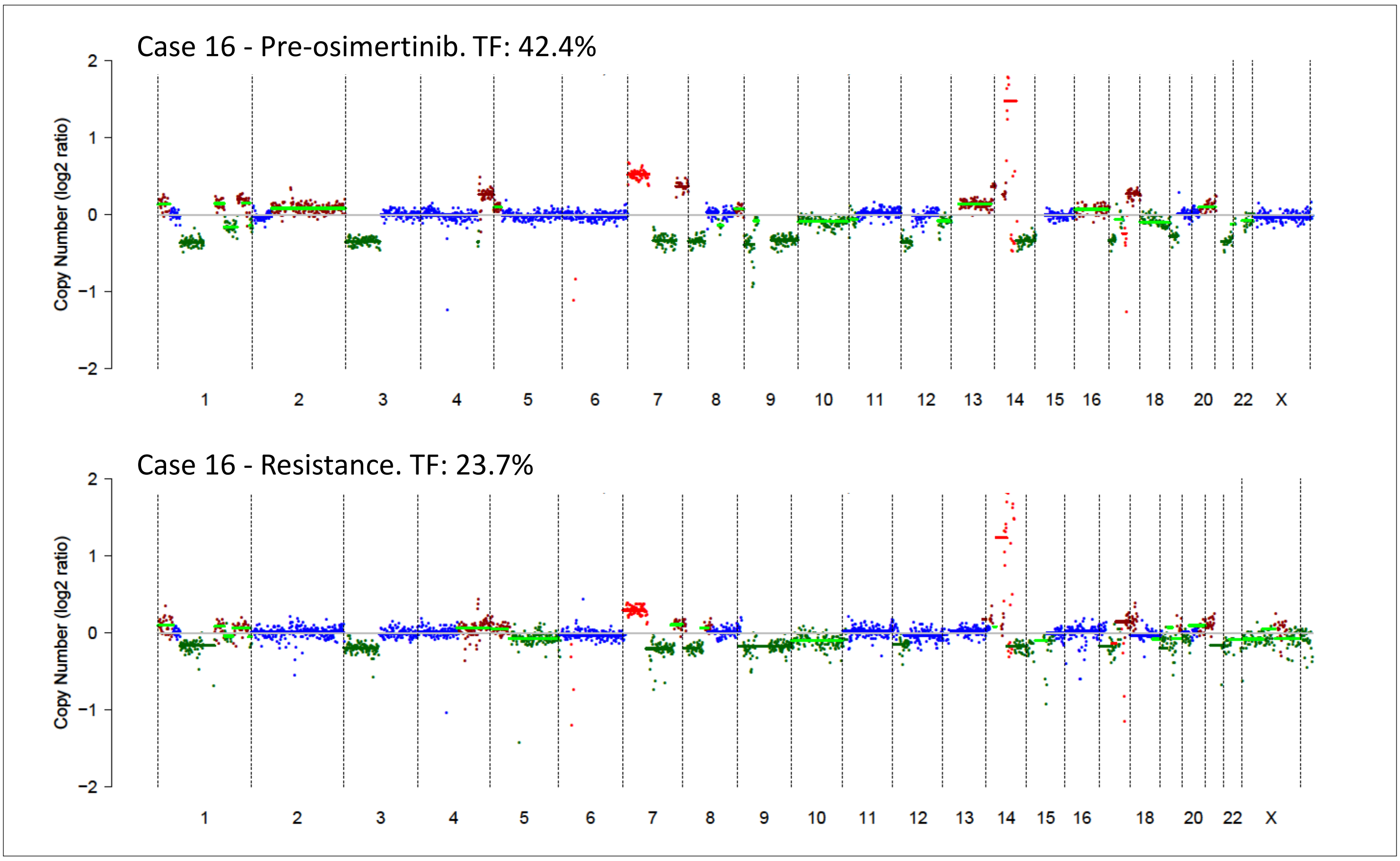
Case 16 - Pre-osimertinib. TF: 42.4%
Case 16 - Resistance. TF: 23.7%
Copy Number (log2 ratio)
2
1
0
−1
−2
1 2 3 4 5 6 7 8 9 10 11 12 13 14 15 16 18 20 22 X

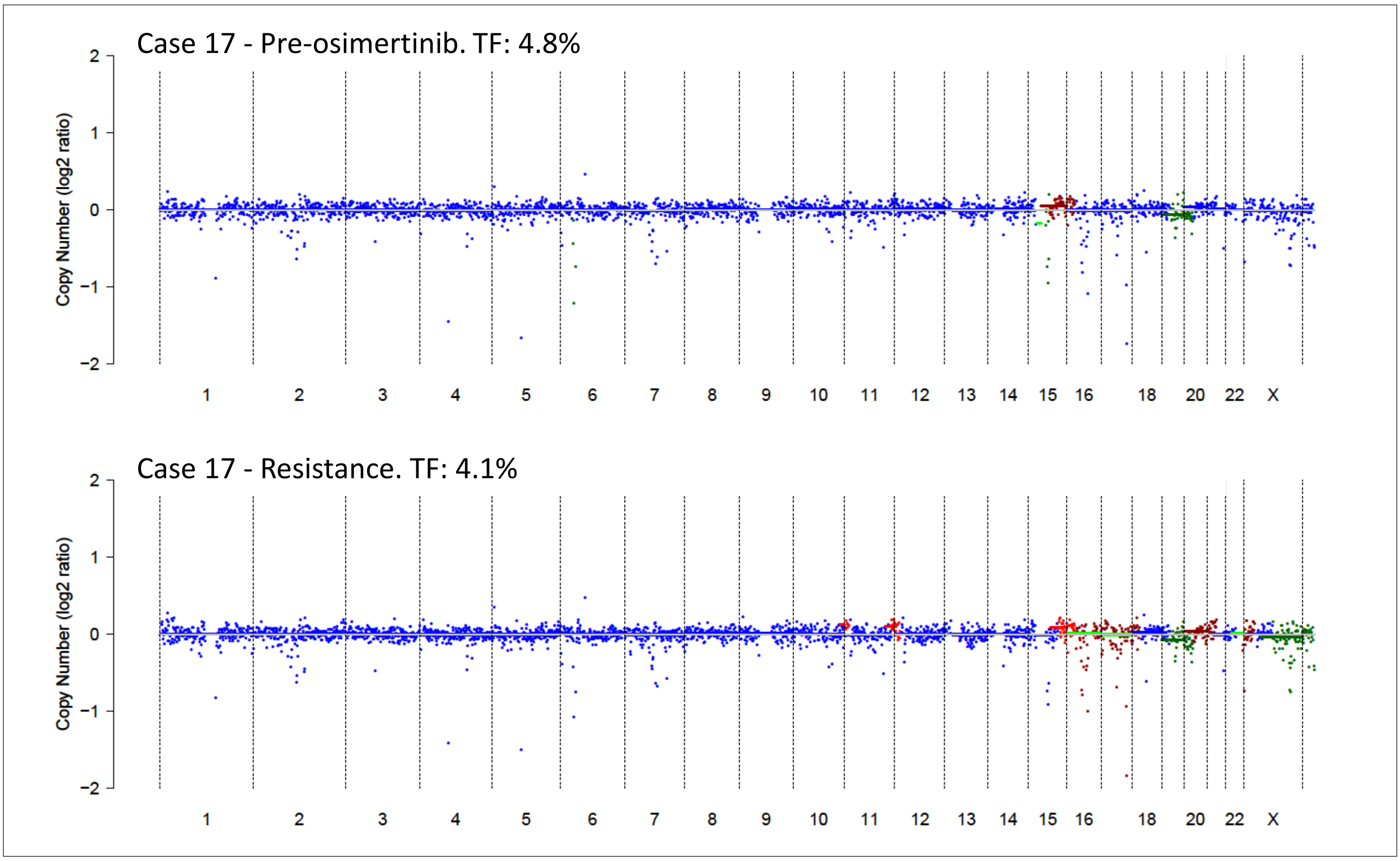
Case 17 - Pre-osimertinib. TF: 4.8%
Copy Number (log2 ratio)
2
1
0
−1
−2
1 2 3 4 5 6 7 8 9 10 11 12 13 14 15 16 18 20 22 X
Case 17 - Resistance. TF: 4.1%
Copy Number (log2 ratio)
2
1
0
−1
−2
1 2 3 4 5 6 7 8 9 10 11 12 13 14 15 16 18 20 22 X

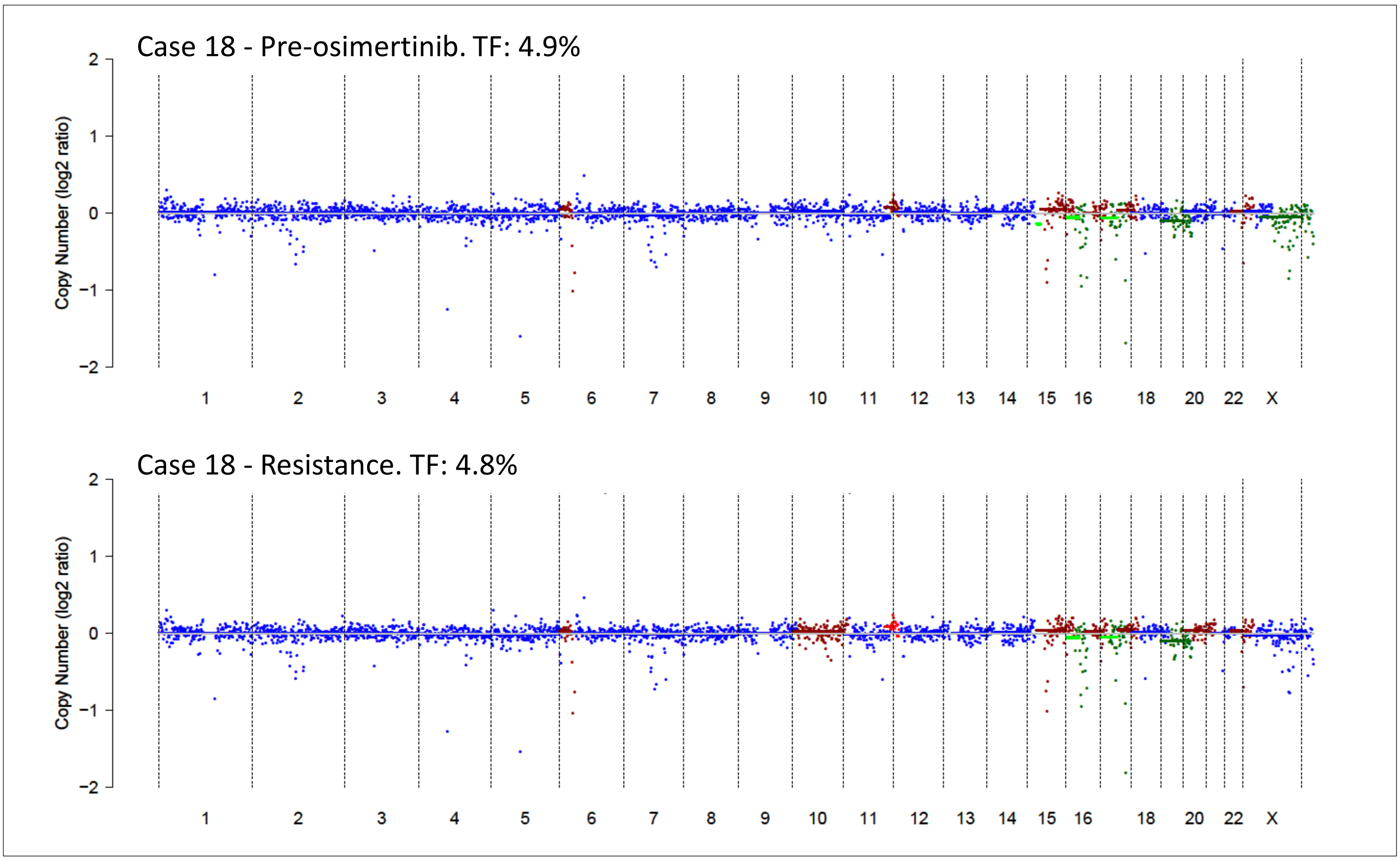
Case 18 - Pre-osimertinib. TF: 4.9%
Copy Number (log2 ratio)
2
1
0
−1
−2
1 2 3 4 5 6 7 8 9 10 11 12 13 14 15 16 18 20 22 X
Case 18 - Resistance. TF: 4.8%
Copy Number (log2 ratio)
2
1
0
−1
−2
1 2 3 4 5 6 7 8 9 10 11 12 13 14 15 16 18 20 22 X

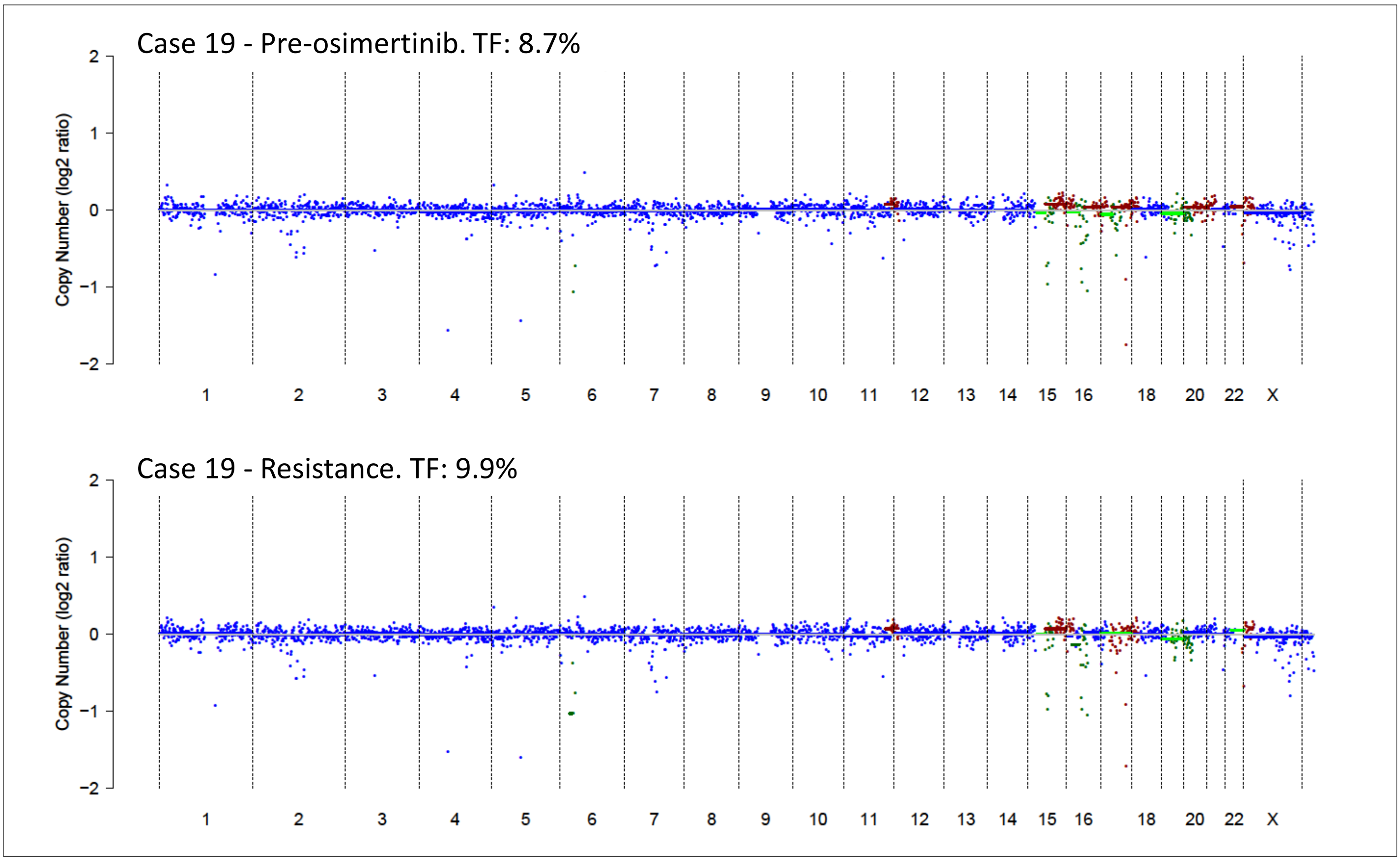
Case 19 - Pre-osimertinib. TF: 8.7%
Case 19 - Resistance. TF: 9.9%
Copy Number (log2 ratio)
2
1
0
−1
−2
1 2 3 4 5 6 7 8 9 10 11 12 13 14 15 16 18 20 22 X

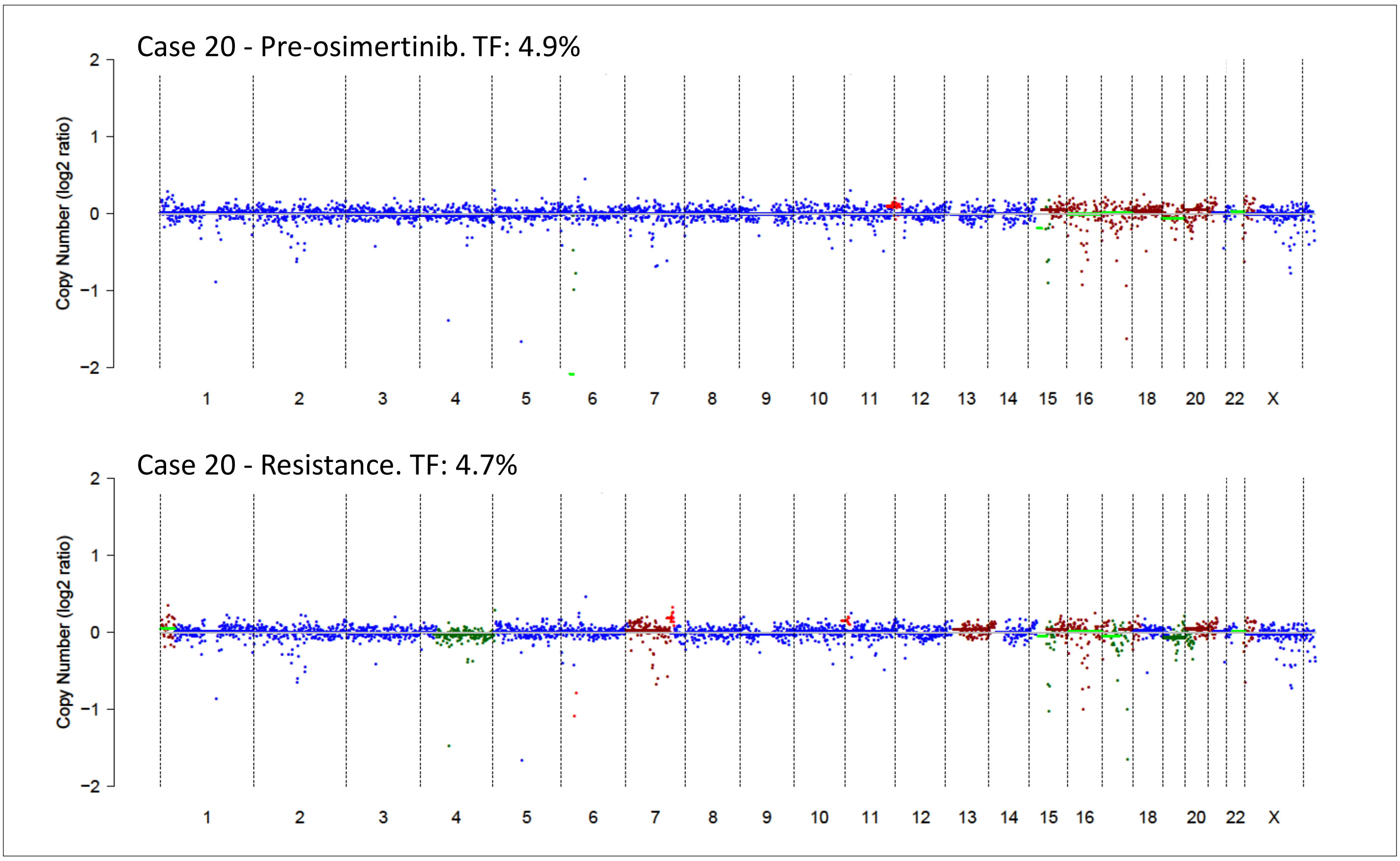
Case 20 - Pre-osimertinib. TF: 4.9%
Case 20 - Resistance. TF: 4.7%
Copy Number (log2 ratio)
2
1
0
−1
−2
1 2 3 4 5 6 7 8 9 10 11 12 13 14 15 16 18 20 22 X

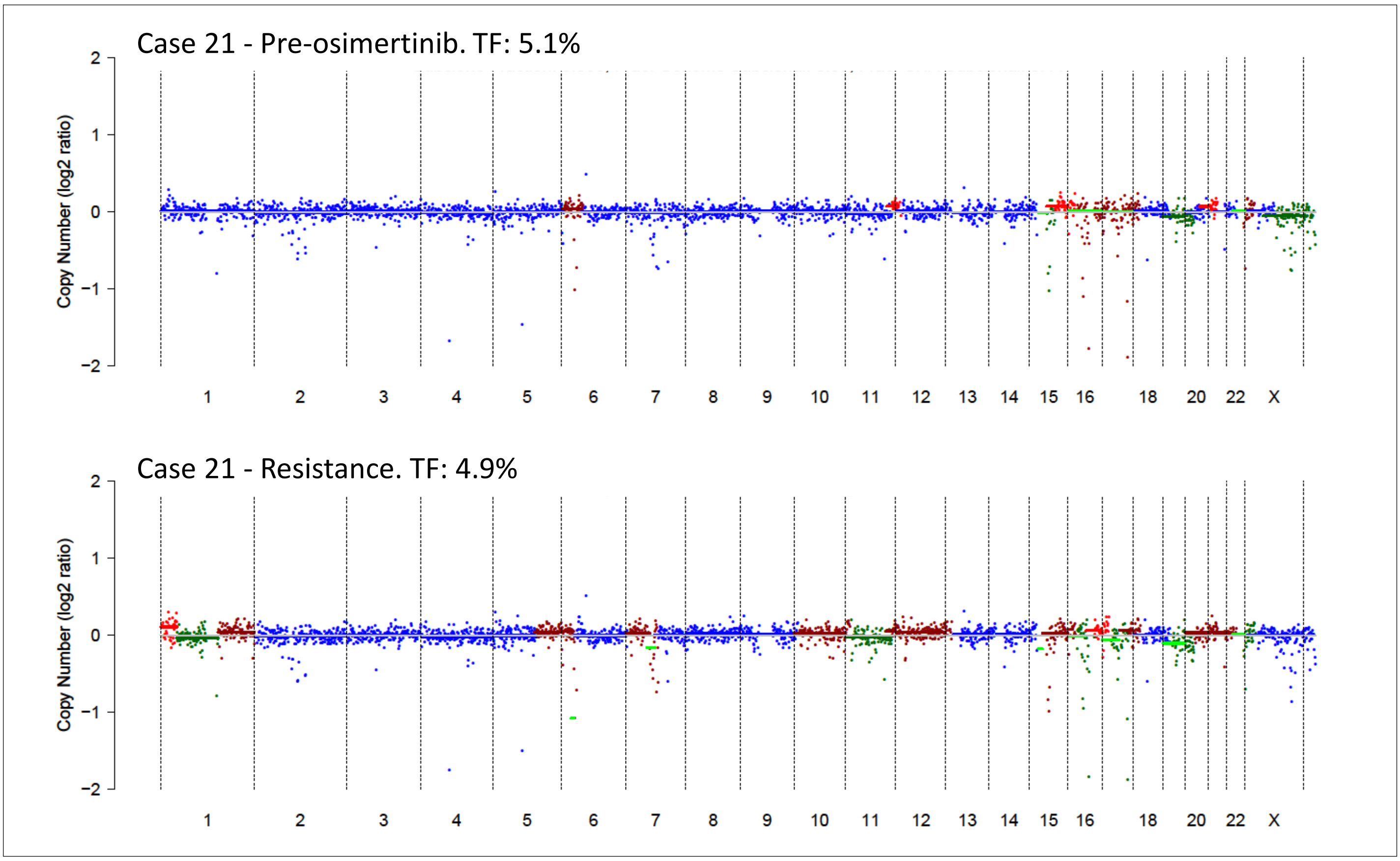
Case 21 - Pre-osimertinib. TF: 5.1%
Case 21 - Resistance. TF: 4.9%
Copy Number (log2 ratio)
2
1
0
−1
−2
1
2
3
4
5
6
7
8
9
10
11
12
13
14
15
16
18
20
22
X

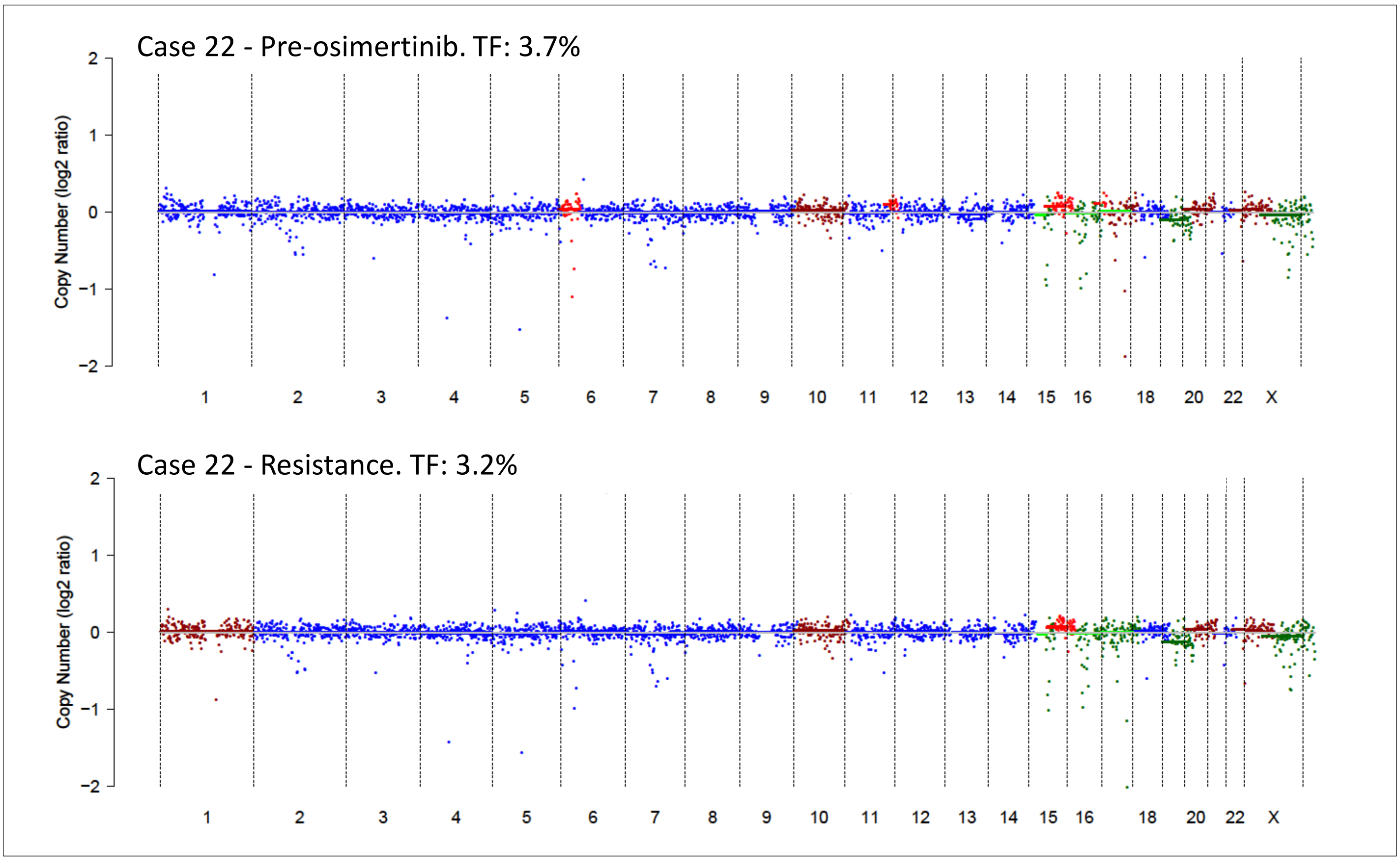
Case 22 - Pre-osimertinib. TF: 3.7%
Case 22 - Resistance. TF: 3.2%
Copy Number (log2 ratio)
2
1
0
−1
−2
1
2
3
4
5
6
7
8
9
10
11
12
13
14
15
16
18
20
22
X

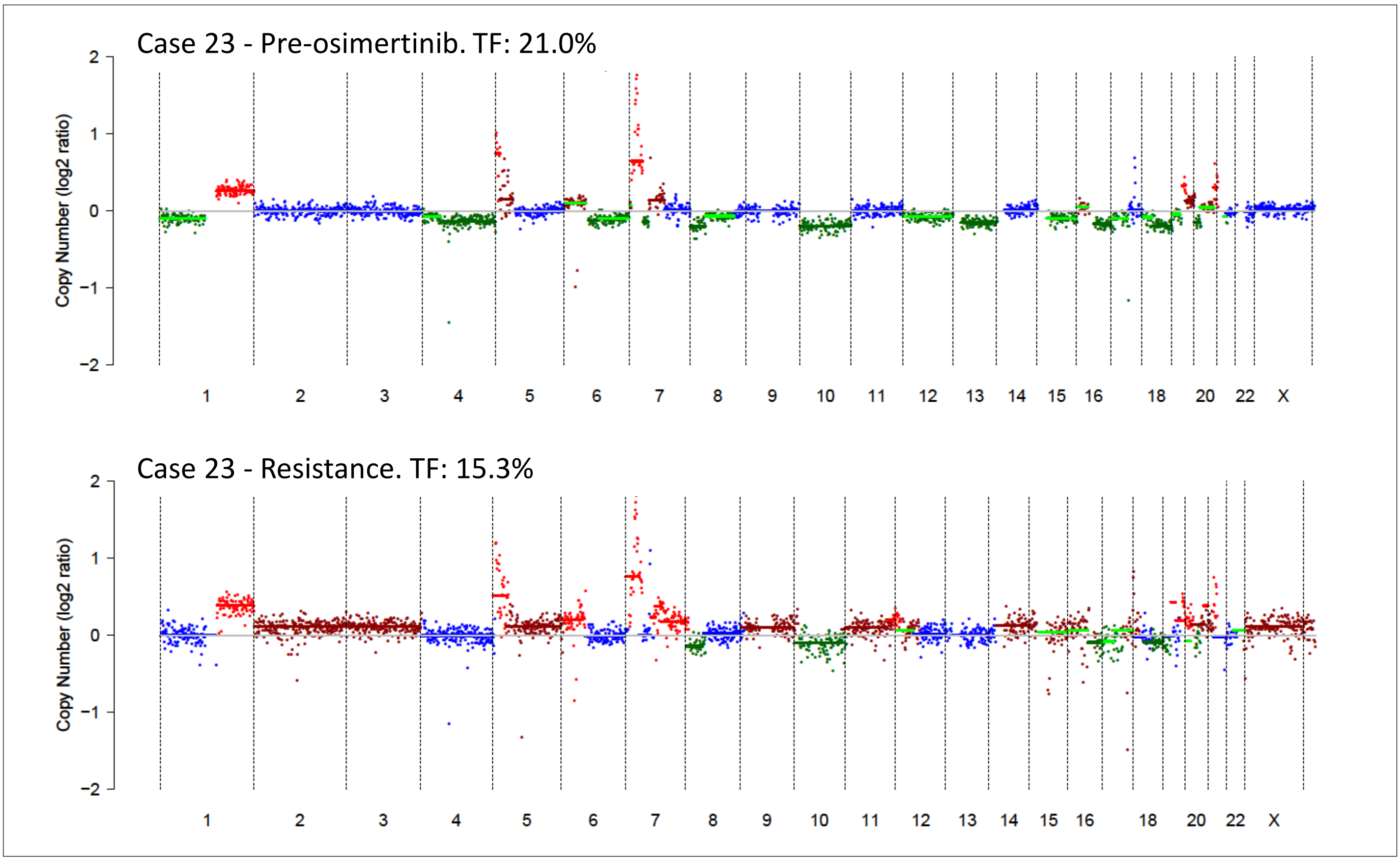

Case 23 - Pre-osimertinib. TF: 21.0%
Copy Number (log2 ratio)
2
1
0
−1
−2
1
2
3
4
5
6
7
8
9
10
11
12
13
14
15
16
18
20
22
X
Case 23 - Resistance. TF: 15.3%
Copy Number (log2 ratio)
2
1
0
−1
−2
1
2
3
4
5
6
7
8
9
10
11
12
13
14
15
16
18
20
22
X

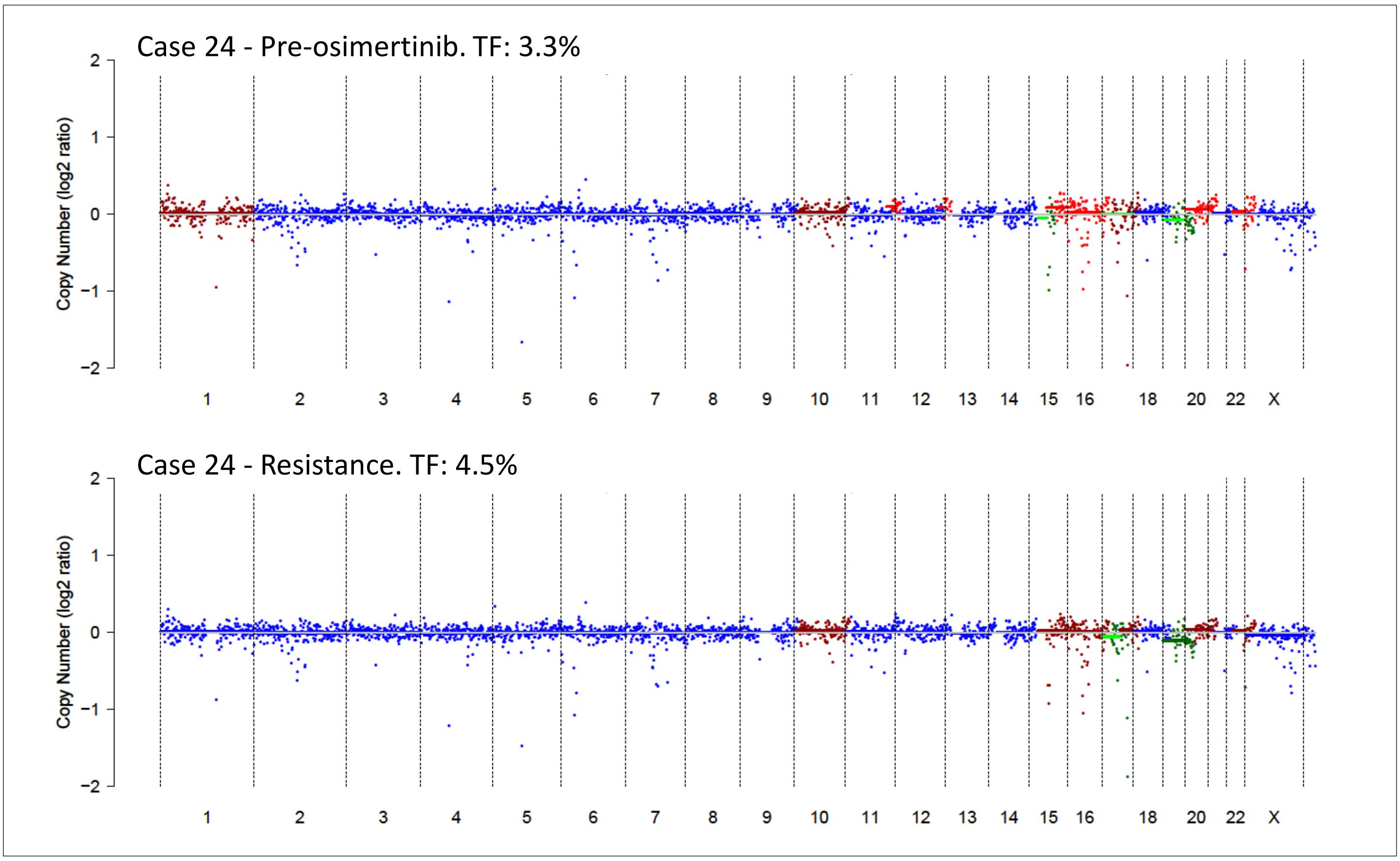

Case 24 - Pre-osimertinib. TF: 3.3%
Copy Number (log2 ratio)
2
1
0
−1
−2
1
2
3
4
5
6
7
8
9
10
11
12
13
14
15
16
18
20
22
X
Case 24 - Resistance. TF: 4.5%
Copy Number (log2 ratio)
2
1
0
−1
−2
1
2
3
4
5
6
7
8
9
10
11
12
13
14
15
16
18
20
22
X

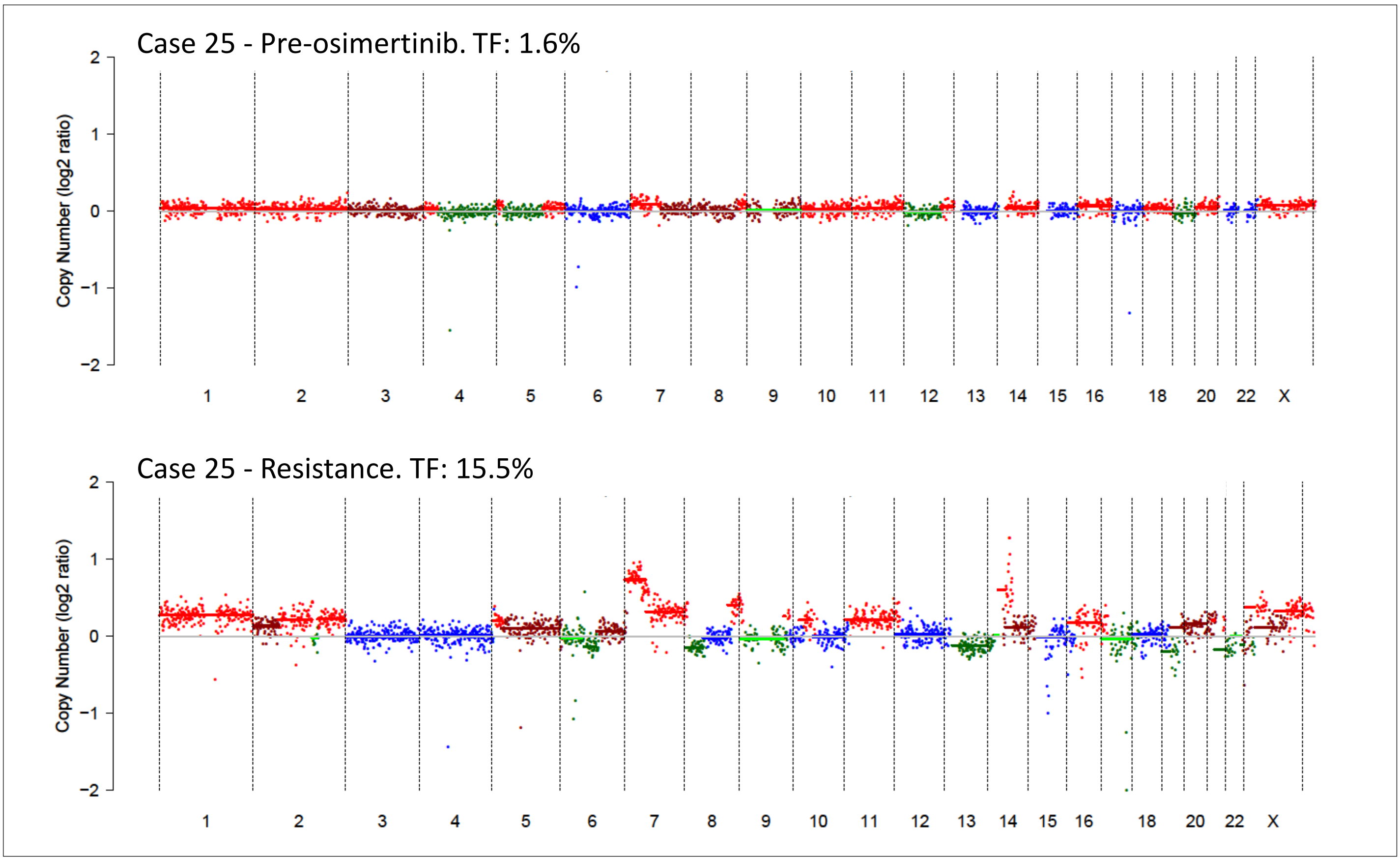
Case 25 - Pre-osimertinib. TF: 1.6%
Case 25 - Resistance. TF: 15.5%
Copy Number (log2 ratio)
1 2 3 4 5 6 7 8 9 10 11 12 13 14 15 16 18 20 22 X

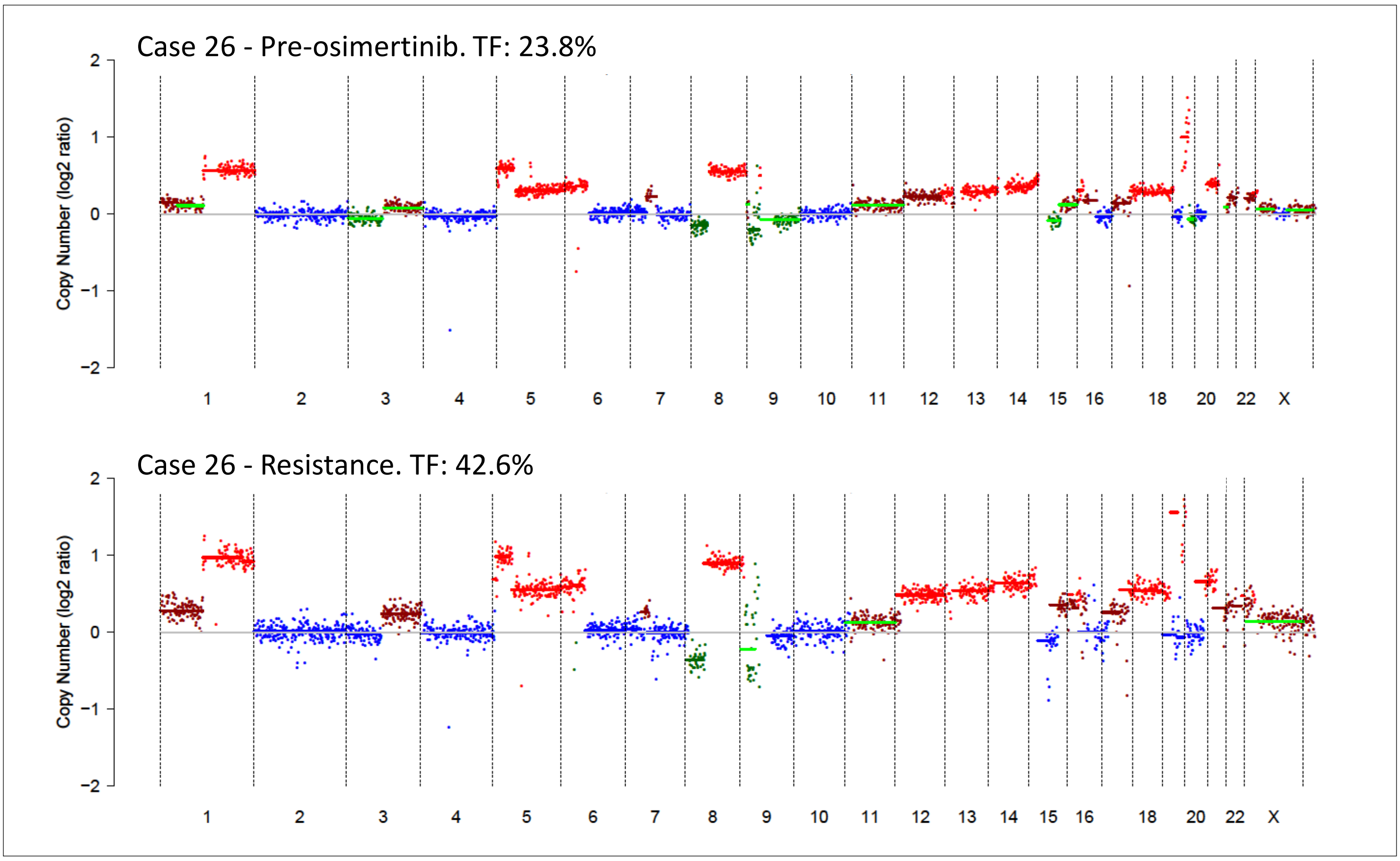
Case 26 - Pre-osimertinib. TF: 23.8%
Case 26 - Resistance. TF: 42.6%
Copy Number (log2 ratio)
1 2 3 4 5 6 7 8 9 10 11 12 13 14 15 16 18 20 22 X

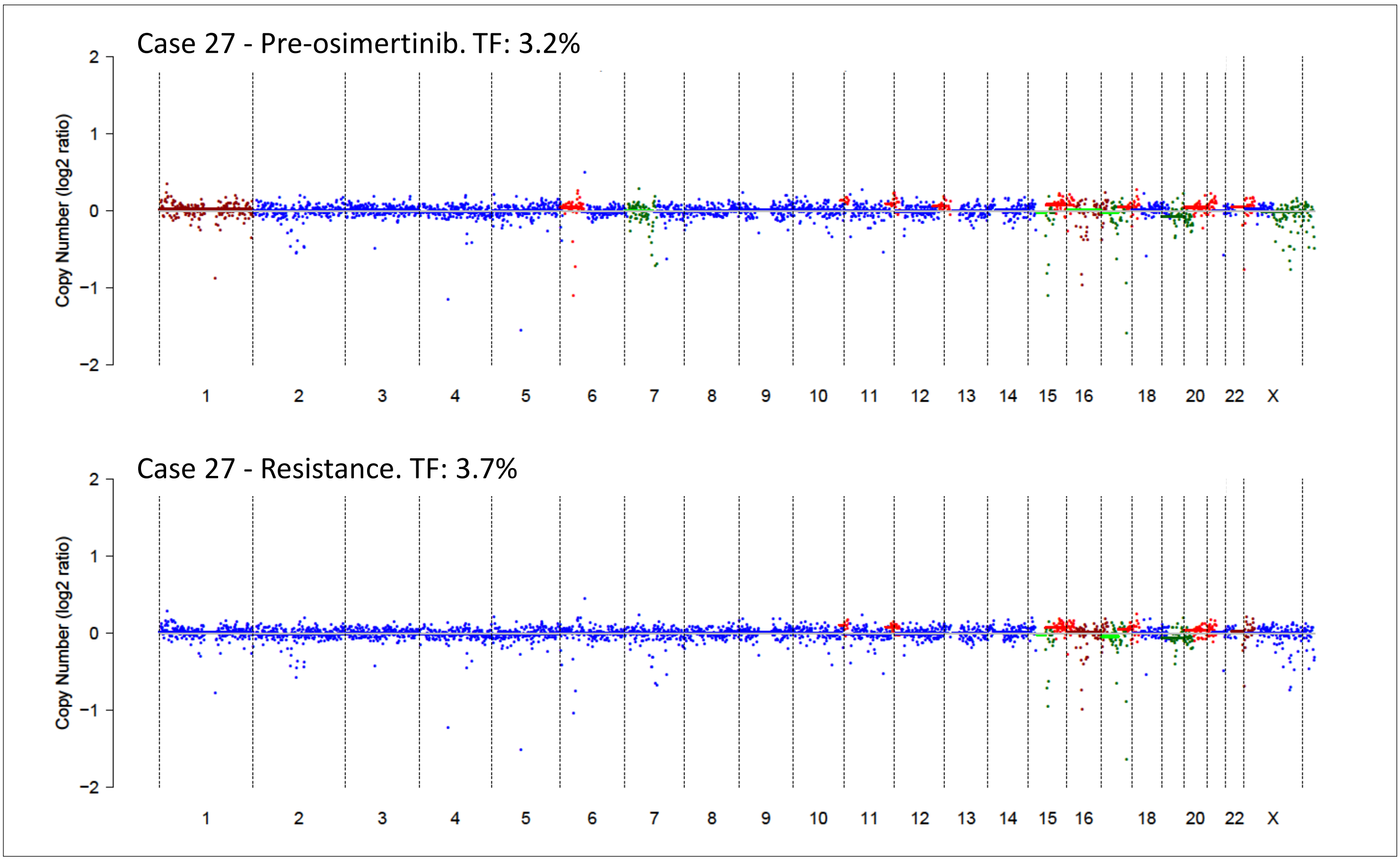

Case 27 - Pre-osimertinib. TF: 3.2%
Case 27 - Resistance. TF: 3.7%
Copy Number (log2 ratio)
2
1
0
−1
−2
1 2 3 4 5 6 7 8 9 10 11 12 13 14 15 16 18 20 22 X

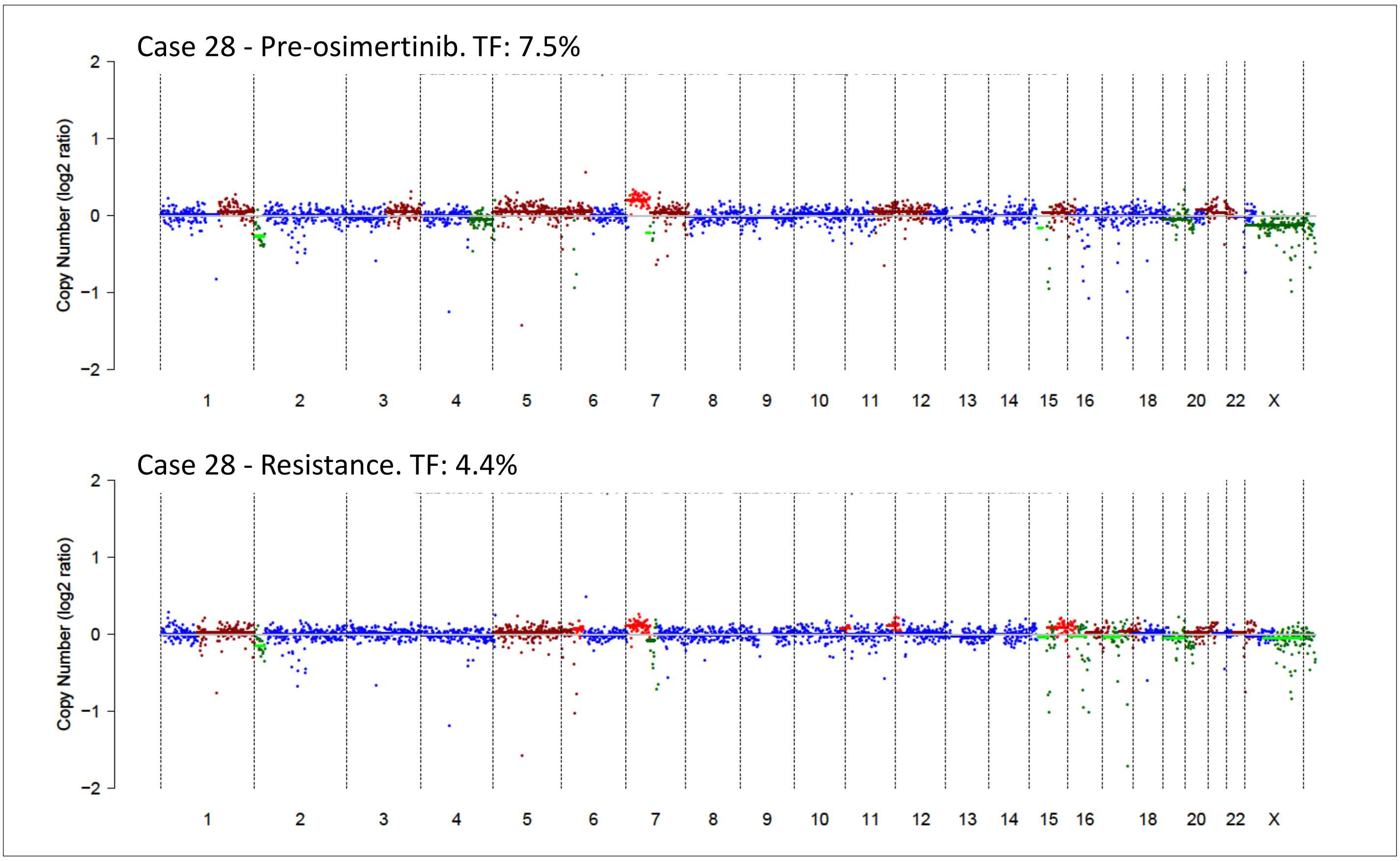

Case 28 - Pre-osimertinib. TF: 7.5%
Case 28 - Resistance. TF: 4.4%
Copy Number (log2 ratio)
2
1
0
−1
−2
1 2 3 4 5 6 7 8 9 10 11 12 13 14 15 16 18 20 22 X

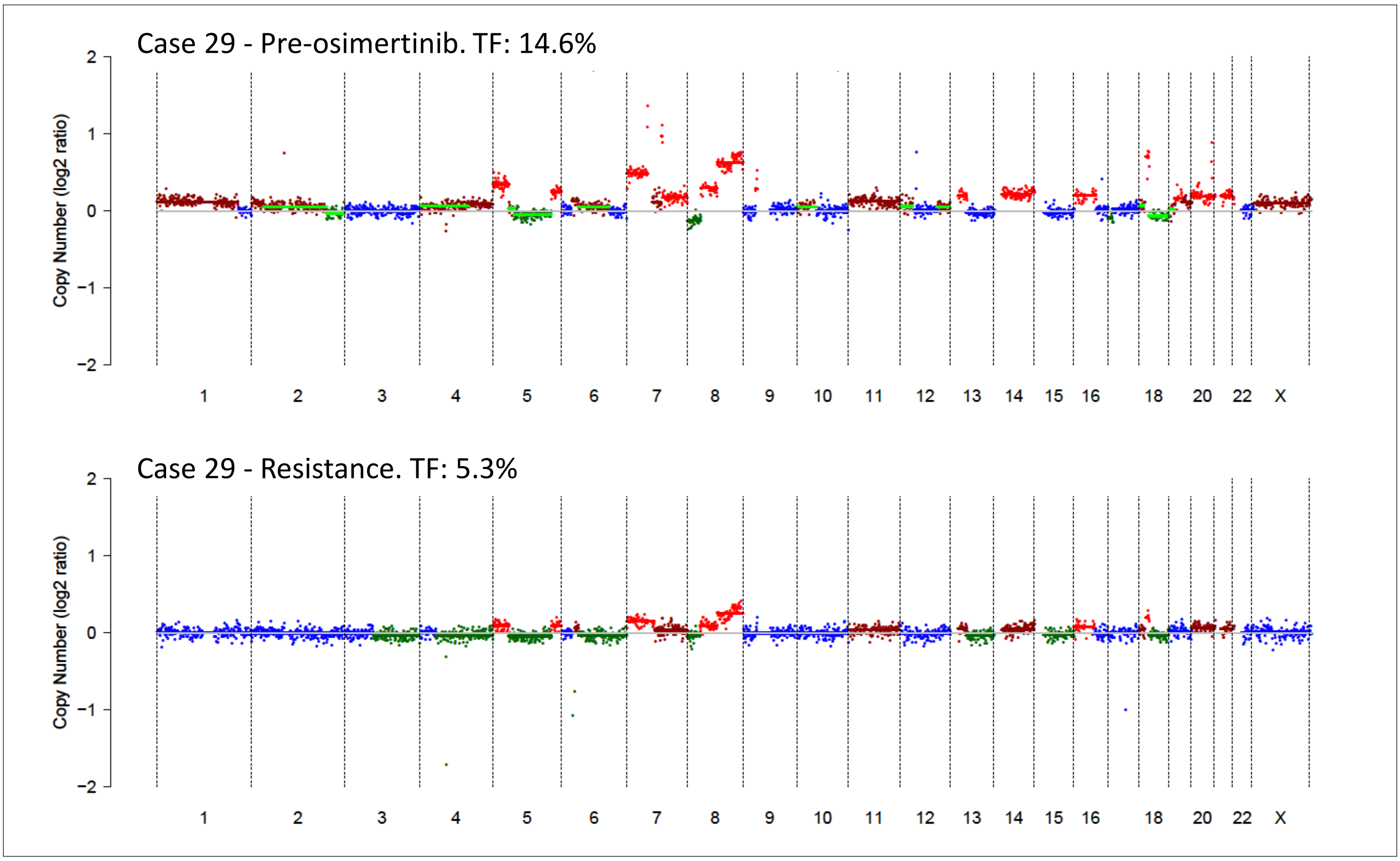
Case 29 - Pre-osimertinib. TF: 14.6%
Case 29 - Resistance. TF: 5.3%
Copy Number (log2 ratio)
2
1
0
−1
−2
1 2 3 4 5 6 7 8 9 10 11 12 13 14 15 16 18 20 22 X

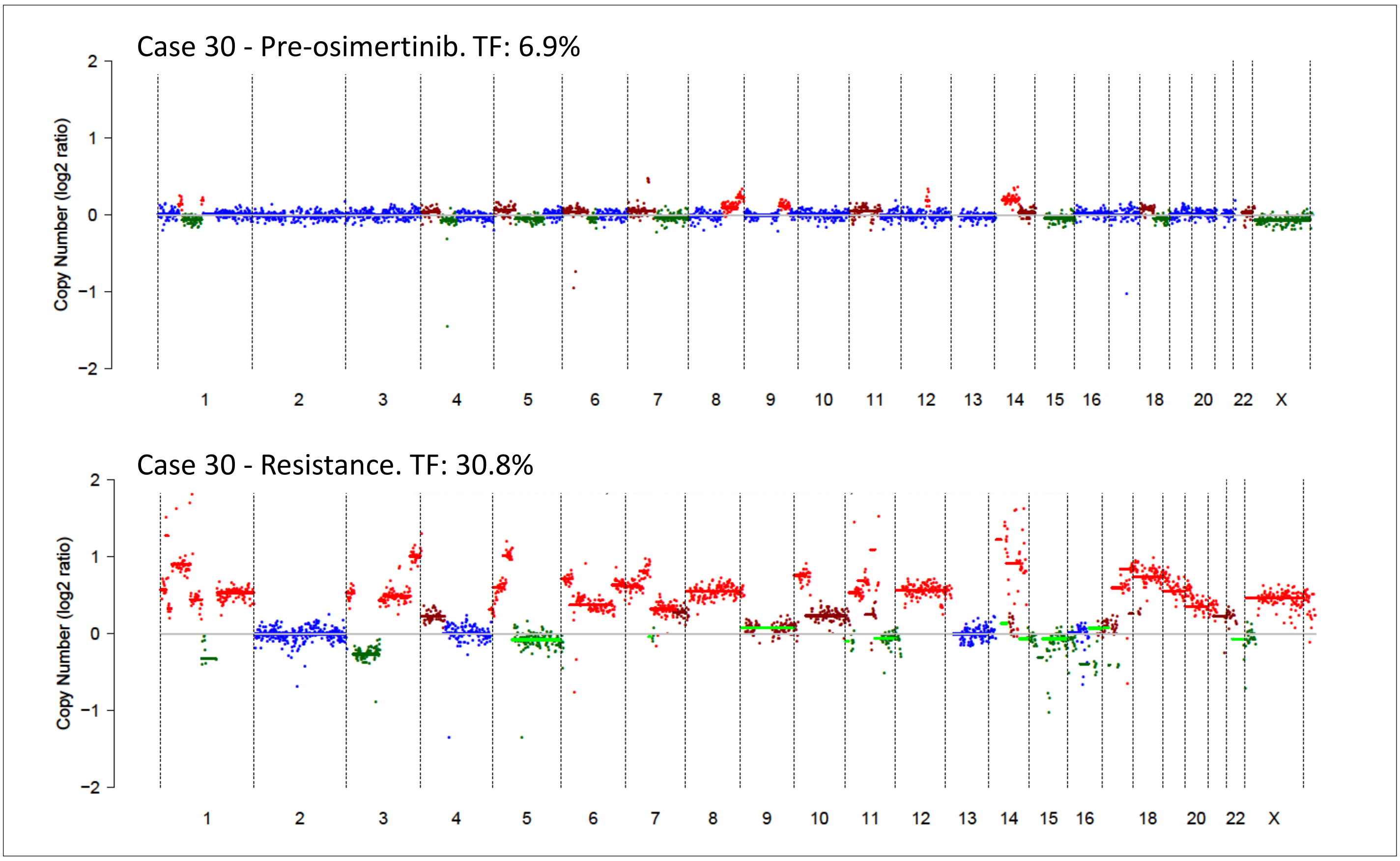
Case 30 - Pre-osimertinib. TF: 6.9%
Case 30 - Resistance. TF: 30.8%
Copy Number (log2 ratio)
2
1
0
−1
−2
1 2 3 4 5 6 7 8 9 10 11 12 13 14 15 16 18 20 22 X

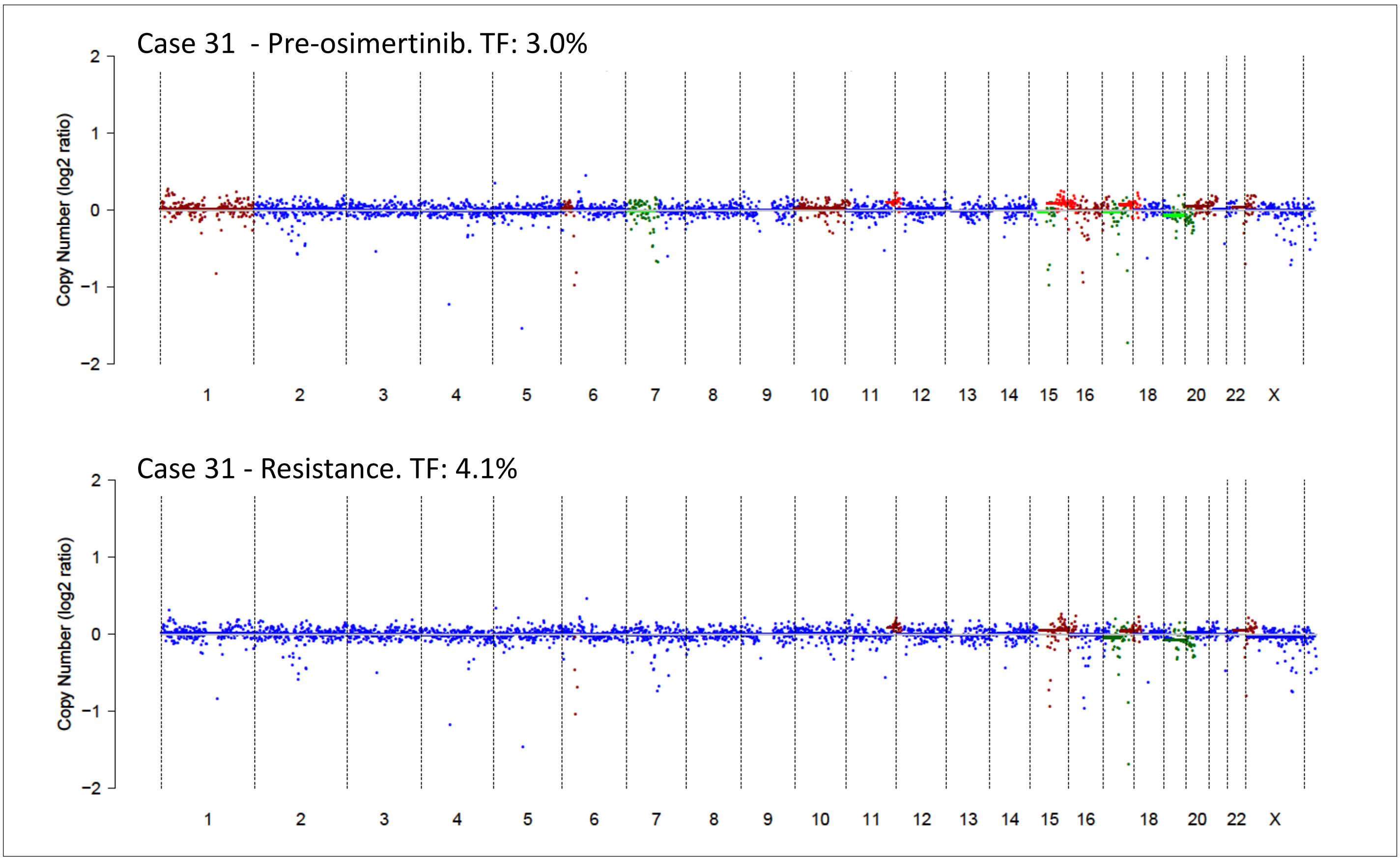
Case 31 - Pre-osimertinib. TF: 3.0%
Copy Number (log2 ratio)
2
1
0
−1
−2
1 2 3 4 5 6 7 8 9 10 11 12 13 14 15 16 18 20 22 X
Case 31 - Resistance. TF: 4.1%
Copy Number (log2 ratio)
2
1
0
−1
−2
1 2 3 4 5 6 7 8 9 10 11 12 13 14 15 16 18 20 22 X

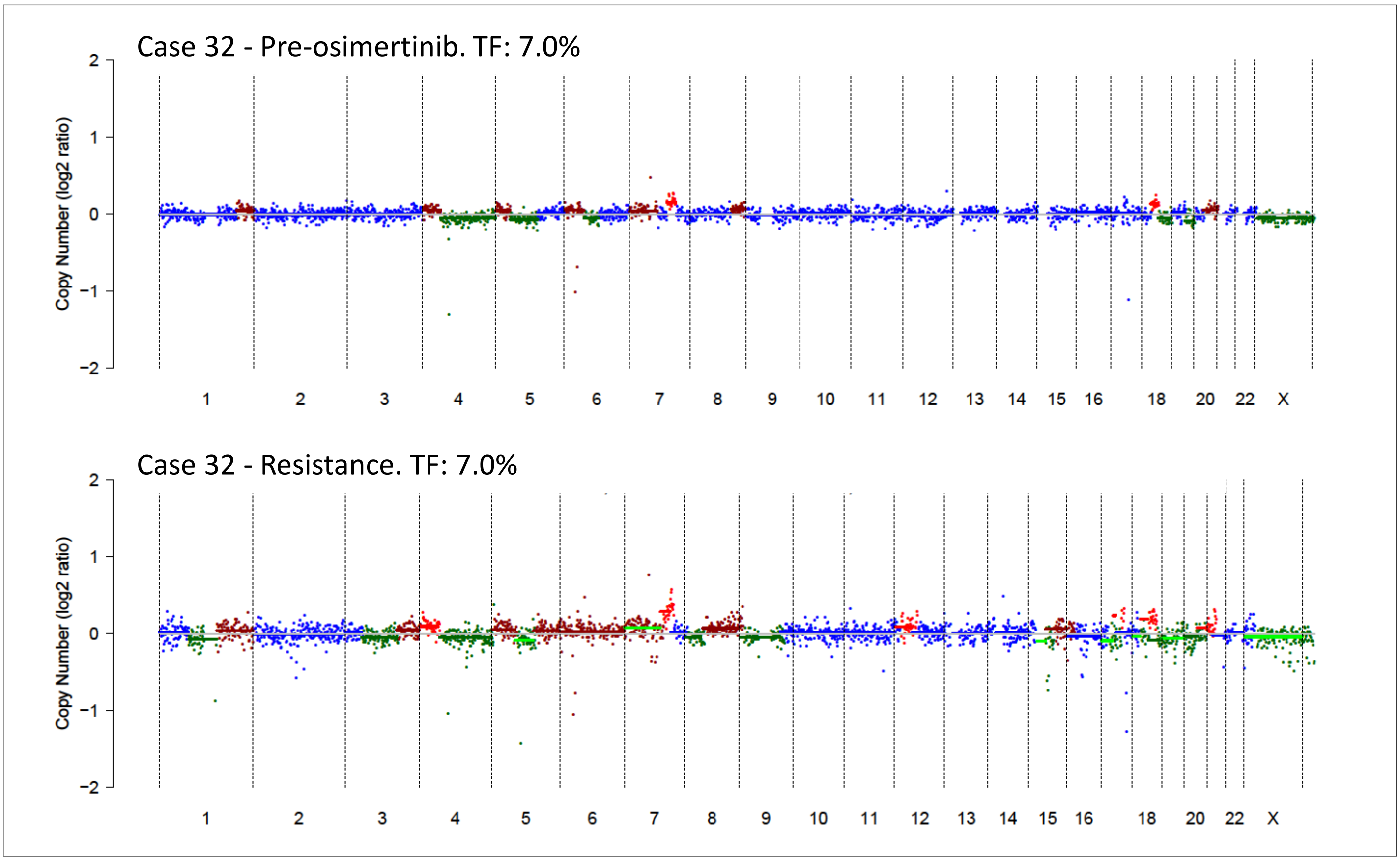
Case 32 - Pre-osimertinib. TF: 7.0%
Copy Number (log2 ratio)
2
1
0
−1
−2
1 2 3 4 5 6 7 8 9 10 11 12 13 14 15 16 18 20 22 X
Case 32 - Resistance. TF: 7.0%
Copy Number (log2 ratio)
2
1
0
−1
−2
1 2 3 4 5 6 7 8 9 10 11 12 13 14 15 16 18 20 22 X

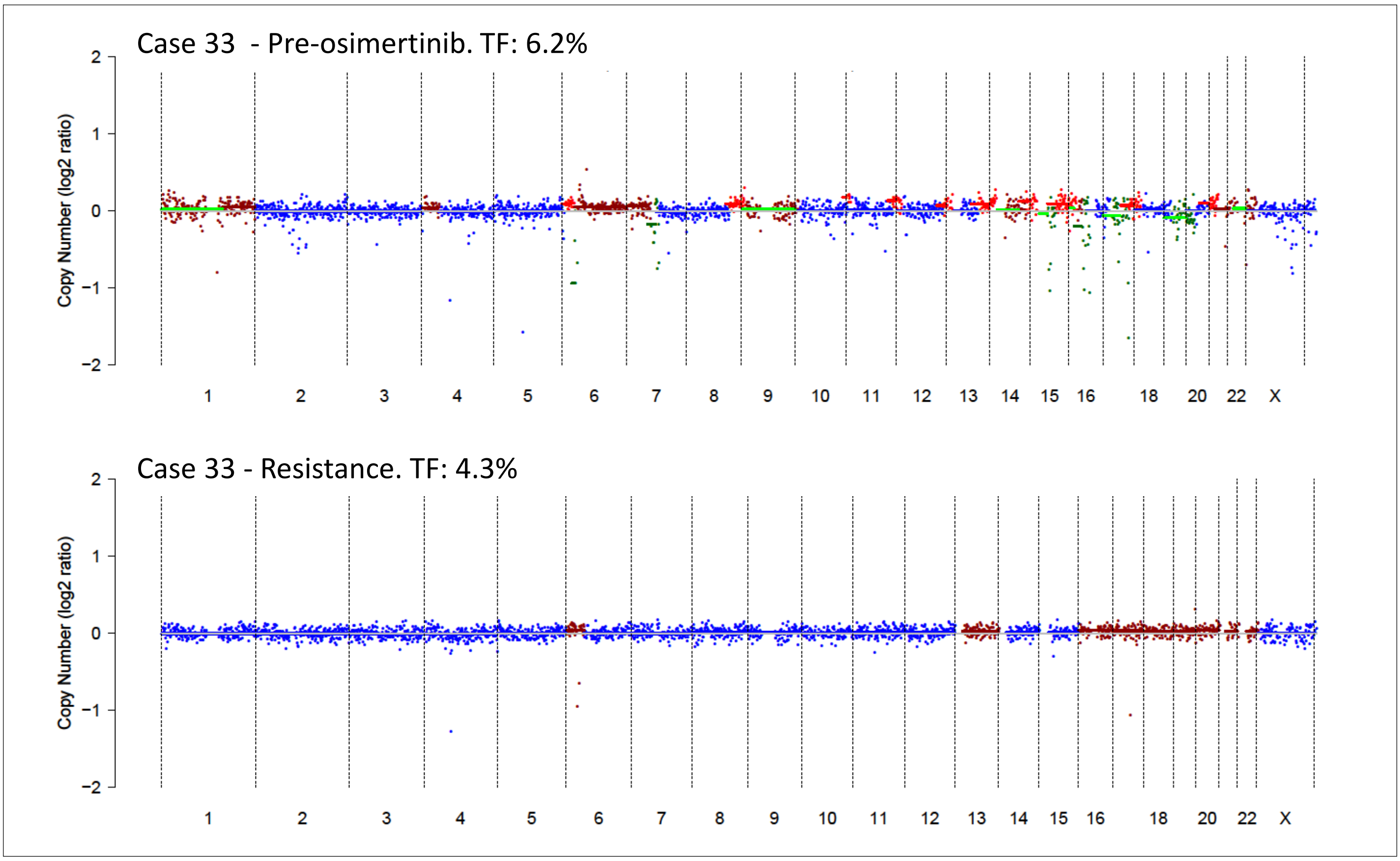
Case 33 - Pre-osimertinib. TF: 6.2%
Case 33 - Resistance. TF: 4.3%
Copy Number (log2 ratio)
2
1
0
−1
−2
1
2
3
4
5
6
7
8
9
10
11
12
13
14
15
16
18
20
22
X

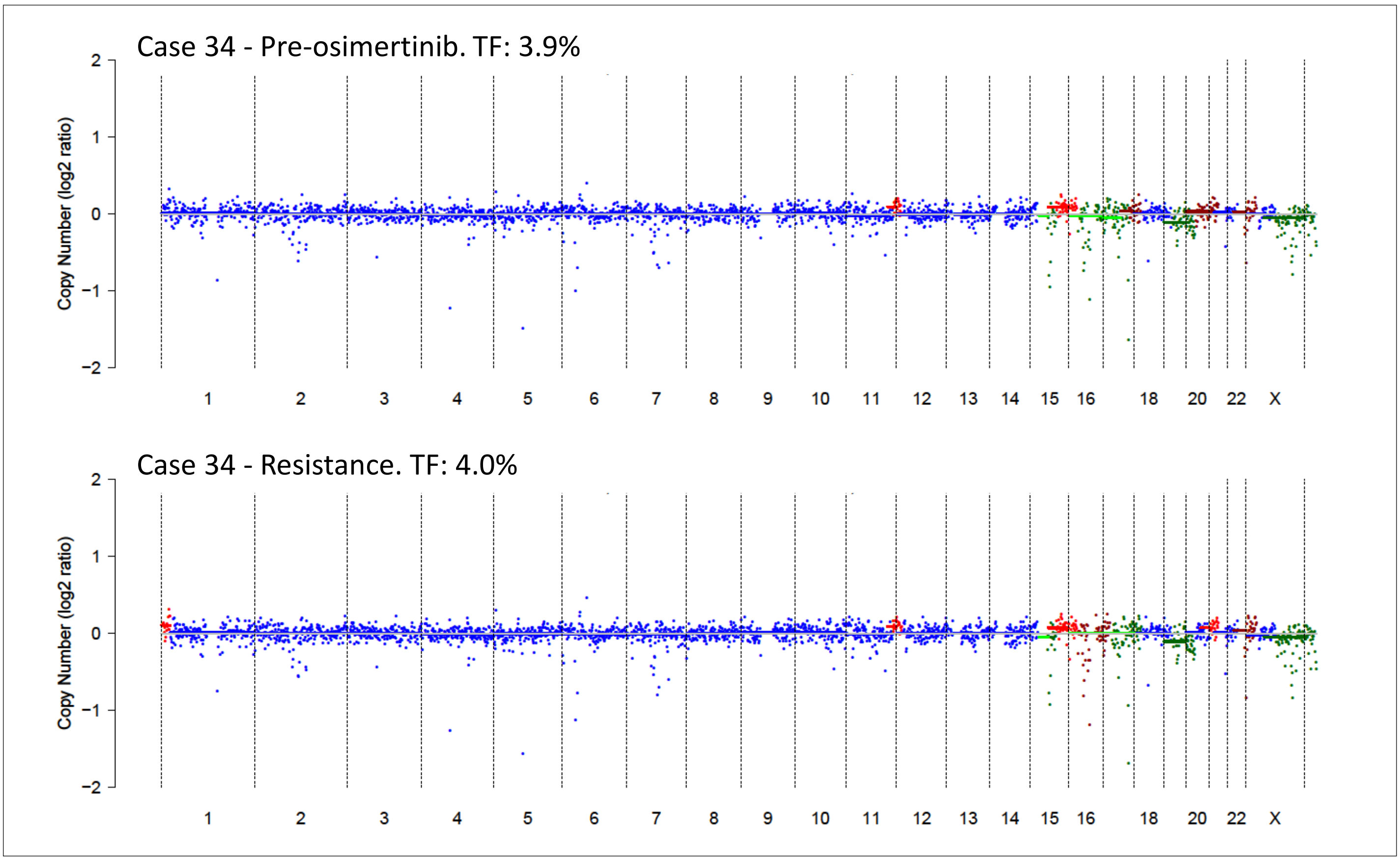
Case 34 - Pre-osimertinib. TF: 3.9%
Case 34 - Resistance. TF: 4.0%
Copy Number (log2 ratio)
2
1
0
−1
−2
1
2
3
4
5
6
7
8
9
10
11
12
13
14
15
16
18
20
22
X

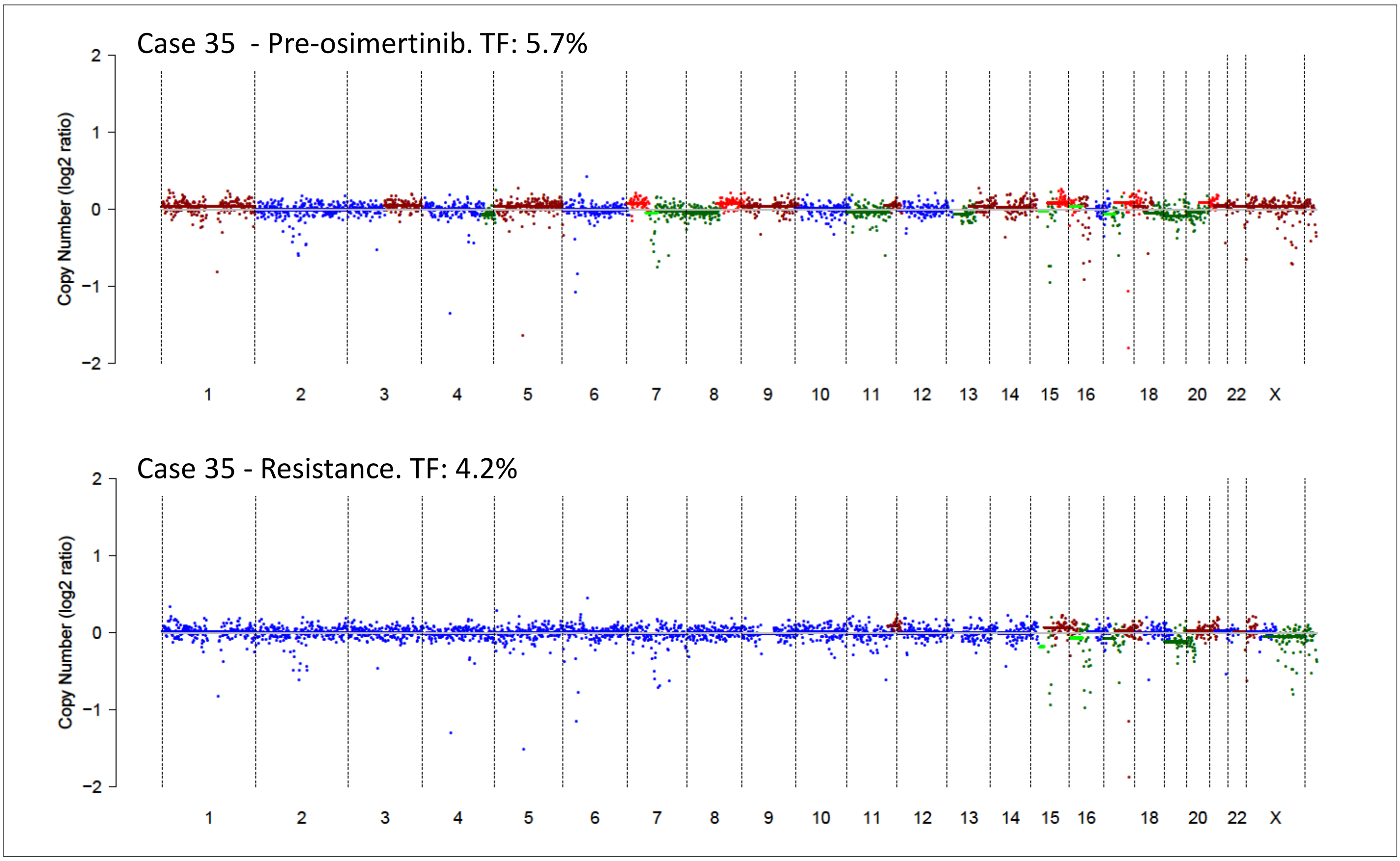
Case 35 - Pre-osimertinib. TF: 5.7%
Case 35 - Resistance. TF: 4.2%
Copy Number (log2 ratio)
2
1
0
−1
−2
1 2 3 4 5 6 7 8 9 10 11 12 13 14 15 16 18 20 22 X

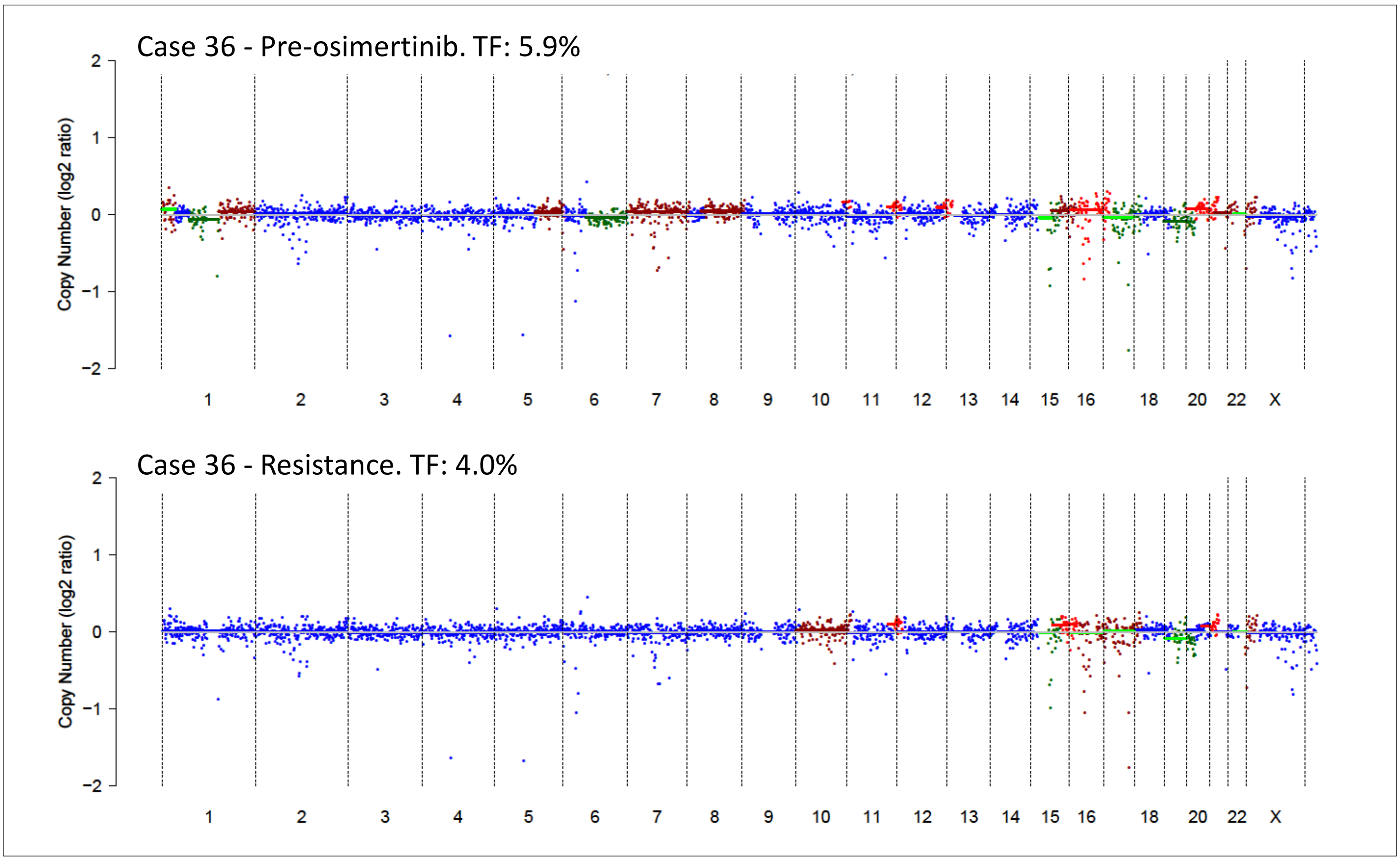
Case 36 - Pre-osimertinib. TF: 5.9%
Case 36 - Resistance. TF: 4.0%
Copy Number (log2 ratio)
2
1
0
−1
−2
1 2 3 4 5 6 7 8 9 10 11 12 13 14 15 16 18 20 22 X

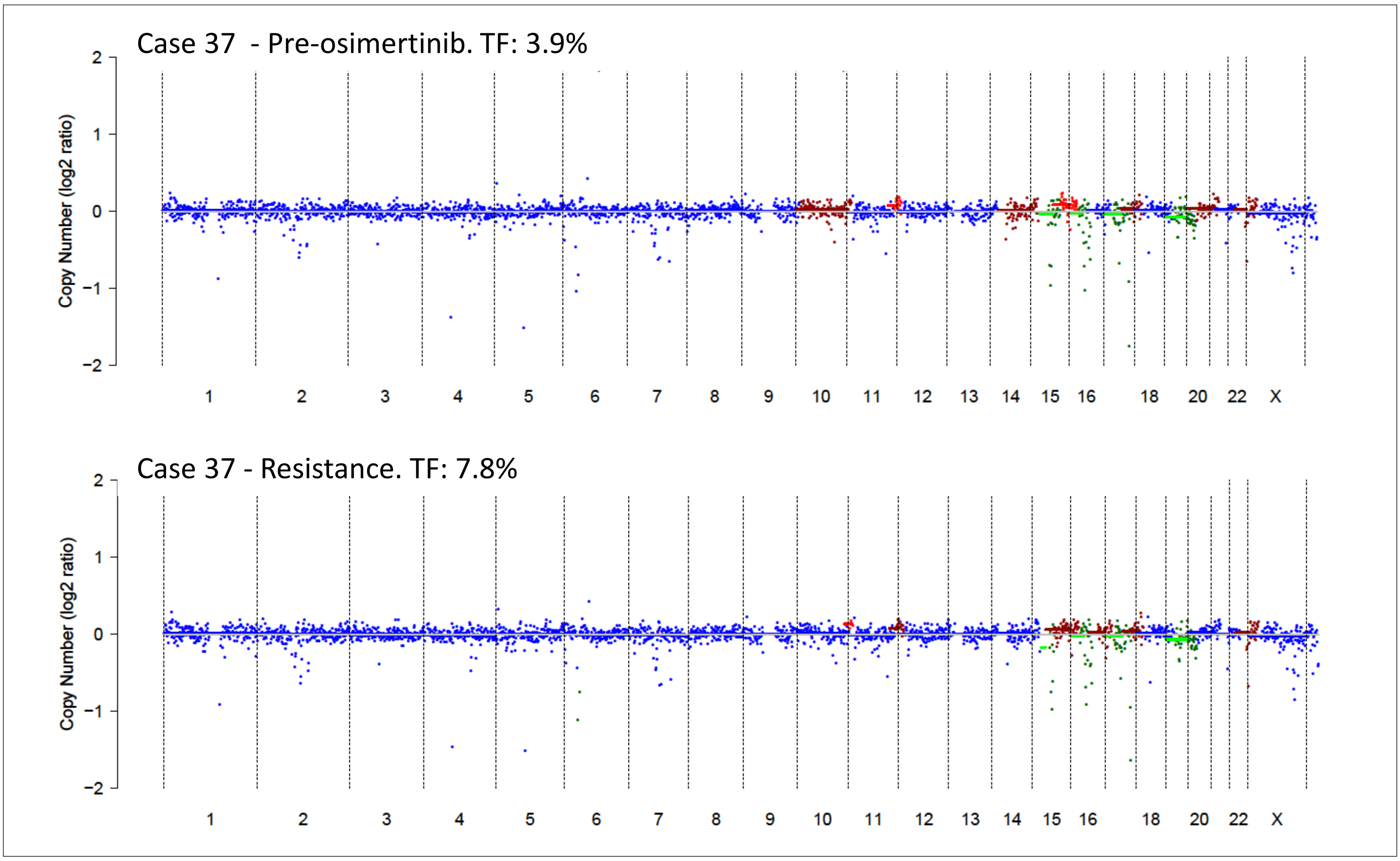

Case 37 - Pre-osimertinib. TF: 3.9%
Copy Number (log2 ratio)
2
1
0
−1
−2
1 2 3 4 5 6 7 8 9 10 11 12 13 14 15 16 18 20 22 X
Case 37 - Resistance. TF: 7.8%
Copy Number (log2 ratio)
2
1
0
−1
−2
1 2 3 4 5 6 7 8 9 10 11 12 13 14 15 16 18 20 22 X

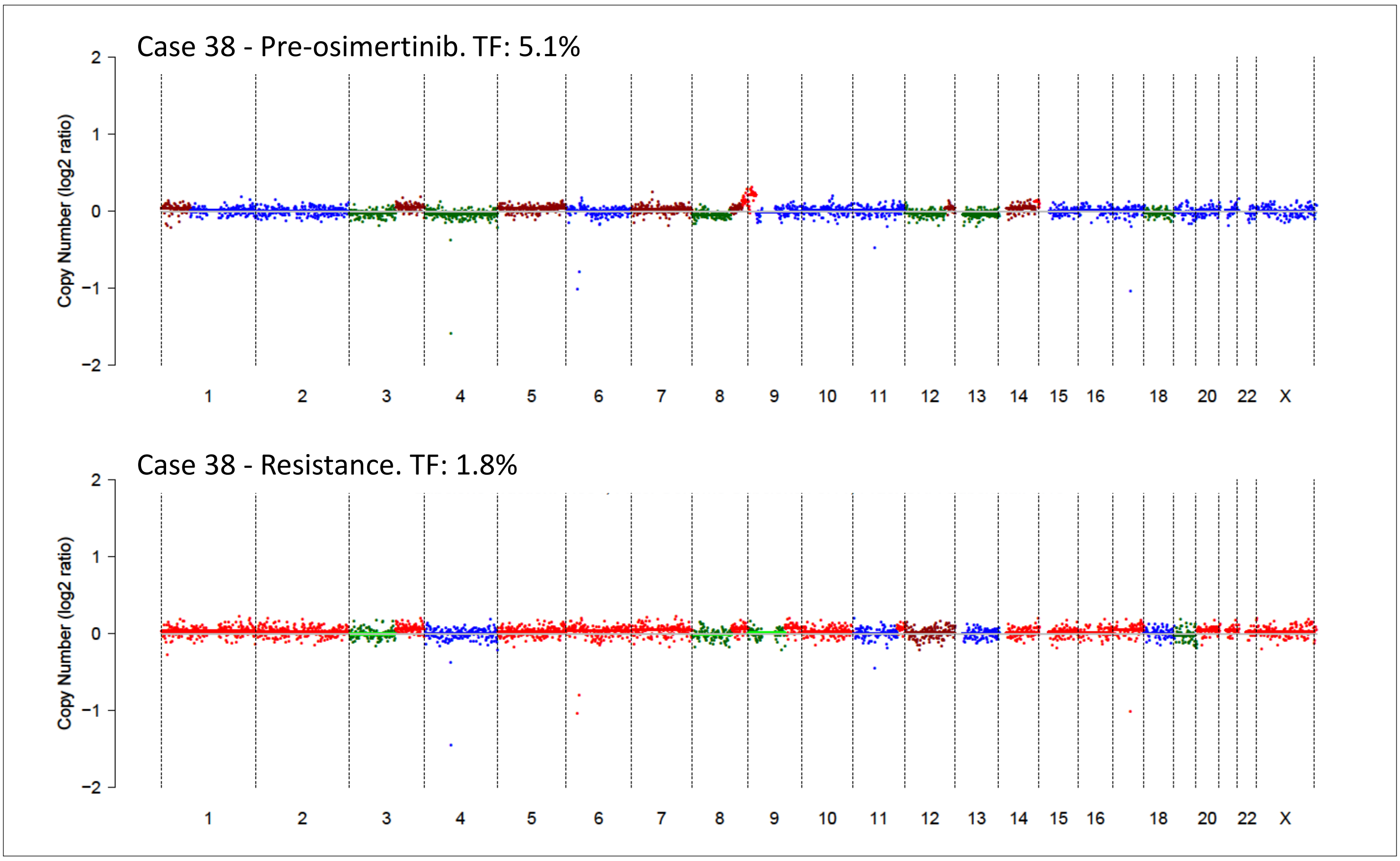

Case 38 - Pre-osimertinib. TF: 5.1%
Copy Number (log2 ratio)
2
1
0
−1
−2
1 2 3 4 5 6 7 8 9 10 11 12 13 14 15 16 18 20 22 X
Case 38 - Resistance. TF: 1.8%
Copy Number (log2 ratio)
2
1
0
−1
−2
1 2 3 4 5 6 7 8 9 10 11 12 13 14 15 16 18 20 22 X

Case 39 - Pre-osimertinib. TF: 3.8%

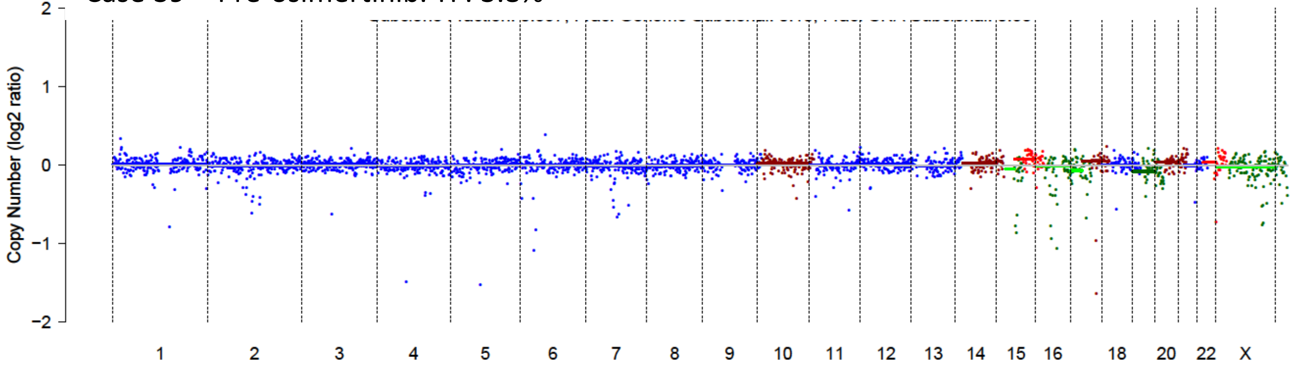

Case 39 - Resistance. TF: 2.9%

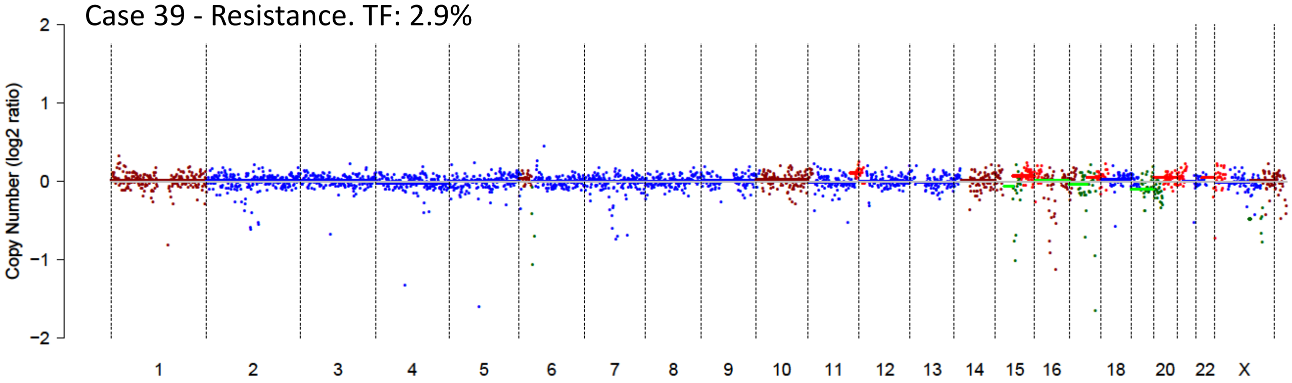

Case 40 - Pre-osimertinib. TF: 7.9%

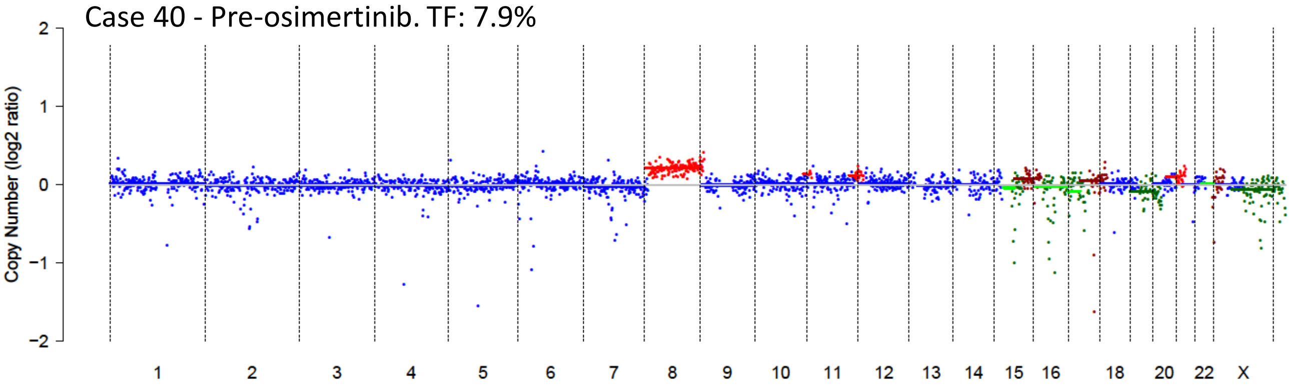

Case 40 - Resistance. TF: 4.5%

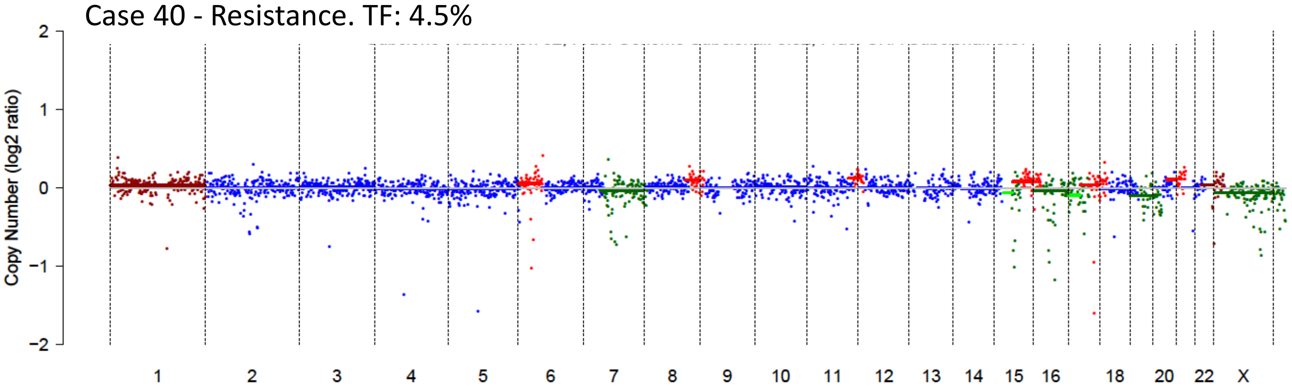

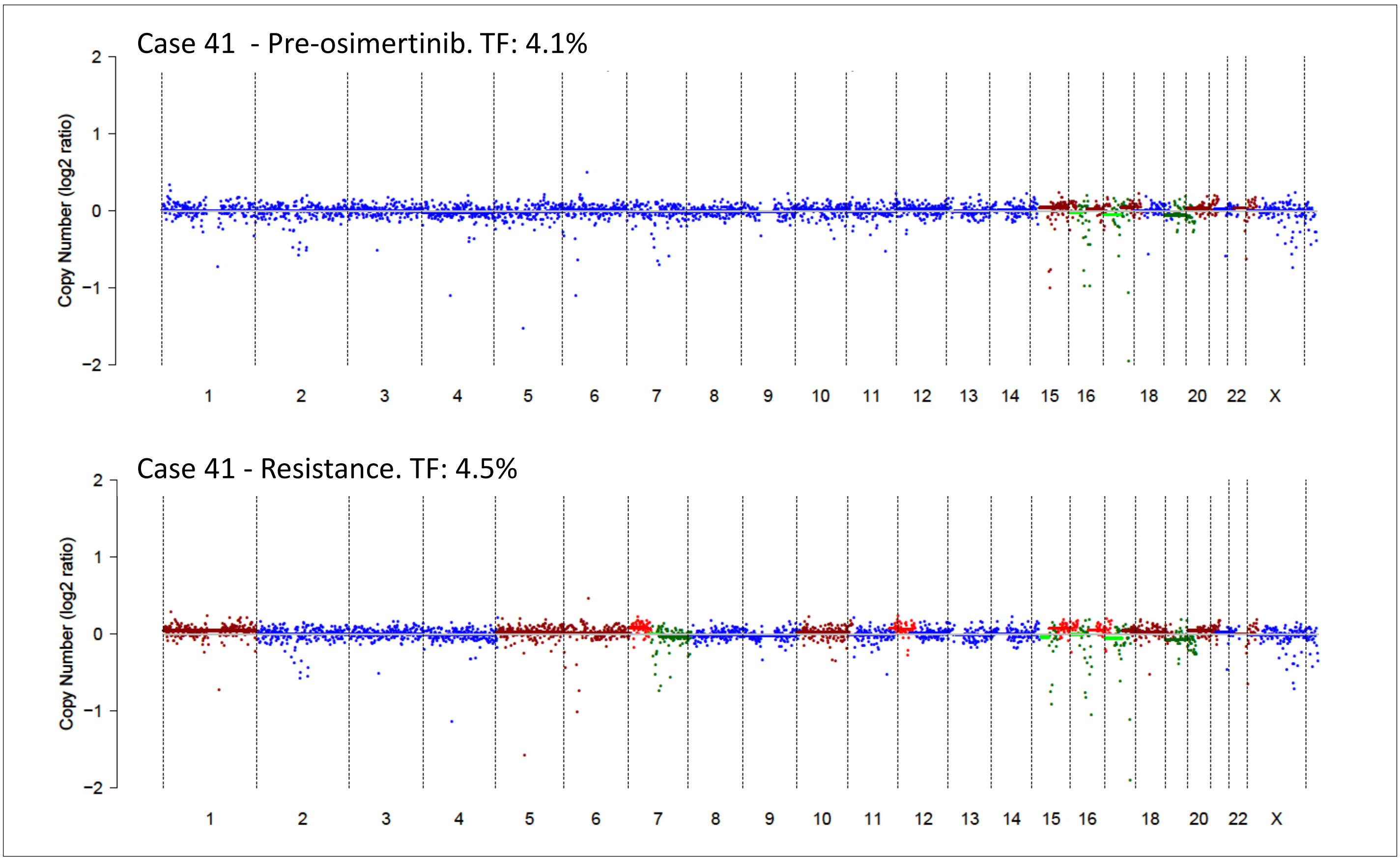
Case 41 - Pre-osimertinib. TF: 4.1%
Case 41 - Resistance. TF: 4.5%
Copy Number (log2 ratio)
2
1
0
−1
−2
1 2 3 4 5 6 7 8 9 10 11 12 13 14 15 16 18 20 22 X

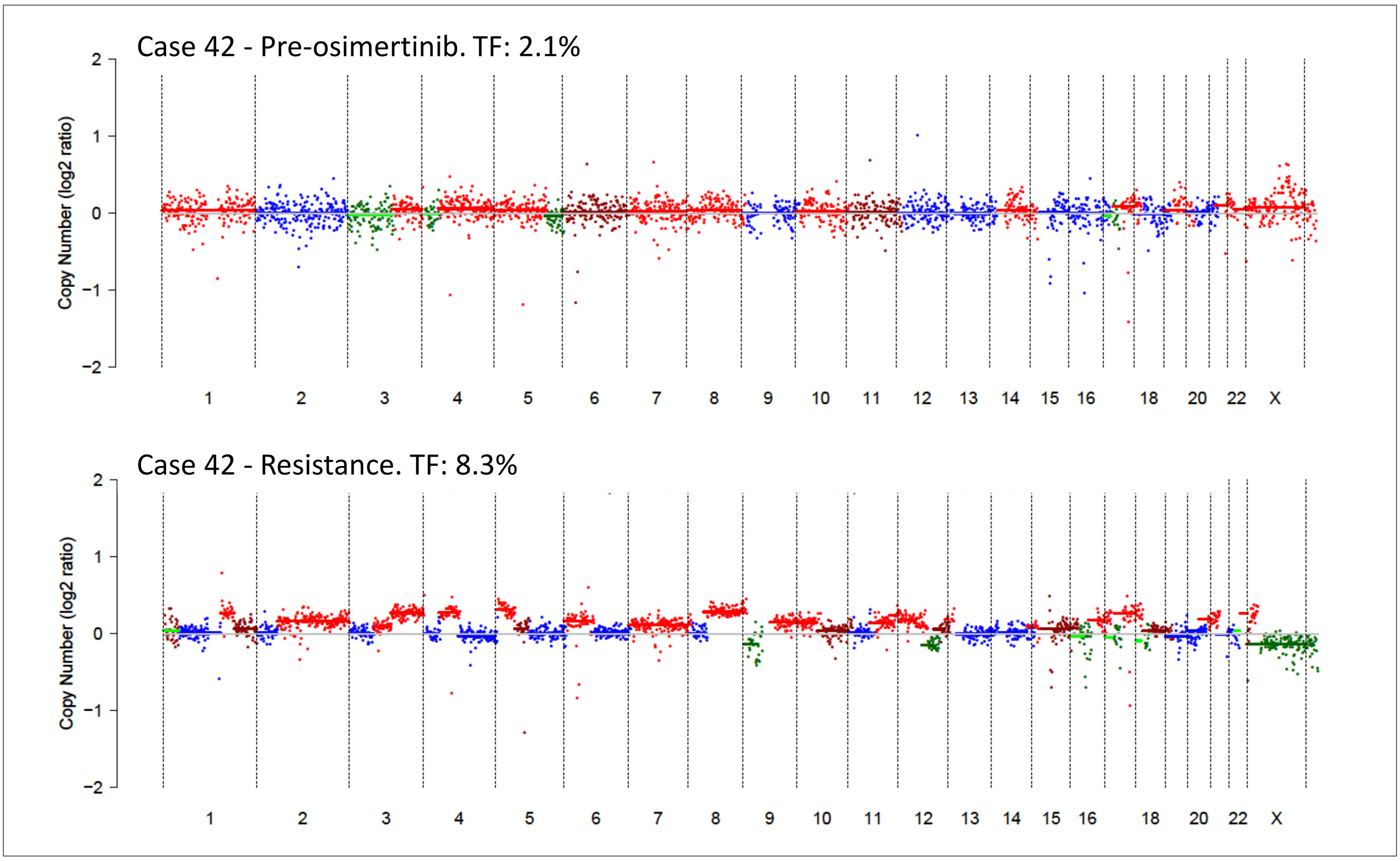
Case 42 - Pre-osimertinib. TF: 2.1%
Case 42 - Resistance. TF: 8.3%
Copy Number (log2 ratio)
2
1
0
−1
−2
1 2 3 4 5 6 7 8 9 10 11 12 13 14 15 16 18 20 22 X

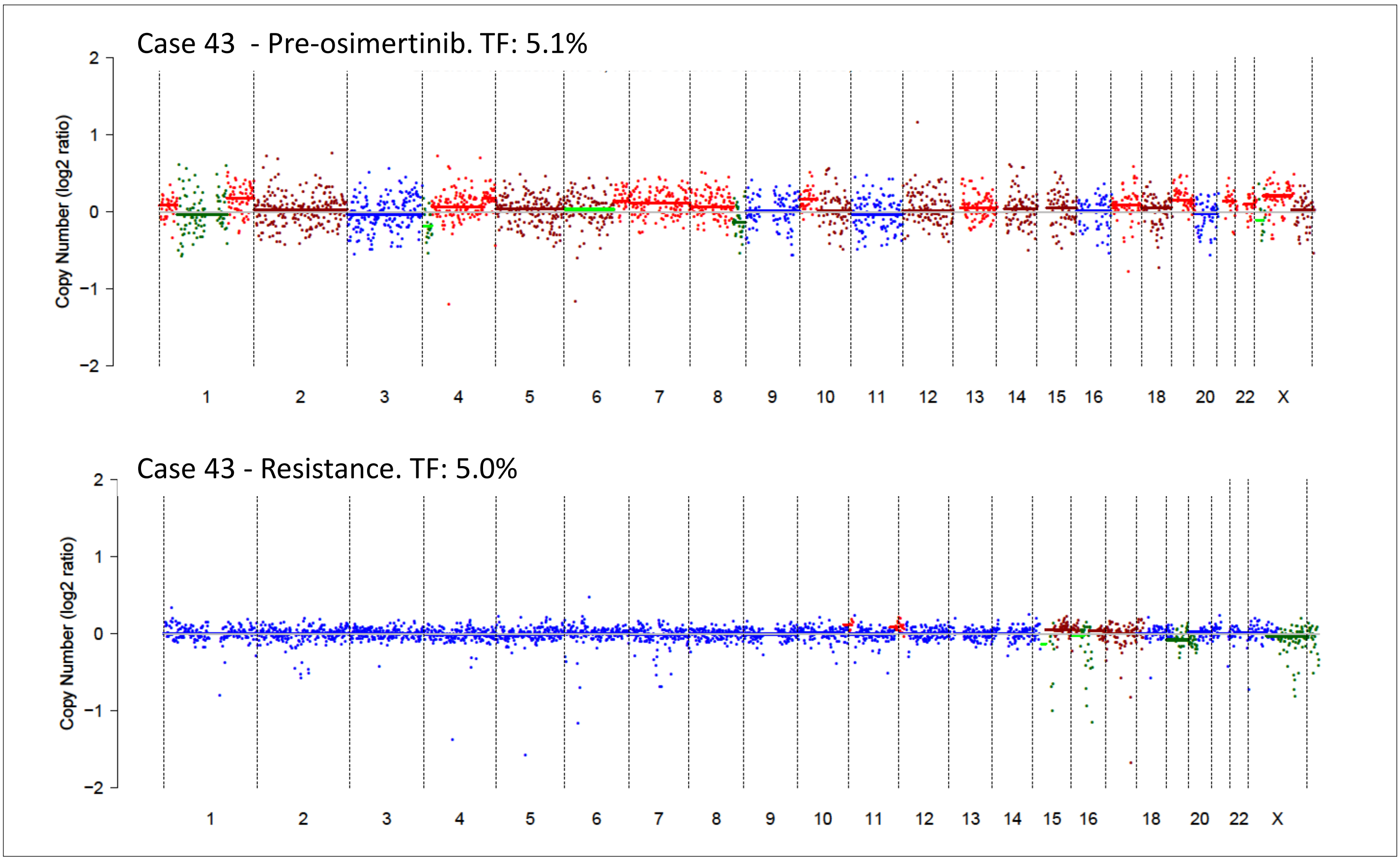

Case 43 - Pre-osimertinib. TF: 5.1%
Copy Number (log2 ratio)
2
1
0
−1
−2
1
2
3
4
5
6
7
8
9
10
11
12
13
14
15
16
18
20
22
X
Case 43 - Resistance. TF: 5.0%
Copy Number (log2 ratio)
2
1
0
−1
−2
1
2
3
4
5
6
7
8
9
10
11
12
13
14
15
16
18
20
22
X
