# Supplementary material for: Somatic Copy-Number Alterations in Plasma Circulating Tumor DNA from Advanced EGFR-Mutated Lung Adenocarcinoma Patients
Source: Biomolecules. 2021 Apr 21;11(5):618. doi: 10.3390/biom11050618 (PMC8143372; doi:10.3390/biom11050618)
Supplement: Supplementary file 1 [file biomolecules-11-00618-s001.zip › Biomolecules 2021_Table S1.pdf]

Patient	Sample	Chr	Start	End	Size [Mb]	Log2Ratio	Gene
**1**	1	chr17	45176973	45289779	0.11	0.68	CDC27
	1*	chr7	115580932	117674526	2.09	0.38	MET
**2**	2	No SCNAs					
	2*	No SCNAs					
**3**	3	No SCNAs					
	3*	No SCNAs					
**4**	4	chr12	69098642	70635098	1.54	0.74	MDM2,YEATS4
		chr12	70635099	70973668	0.34	0.86	CNOT2,KCNMB4
	4*	No SCNAs					
**5**	5	No SCNAs					
	5*	No SCNAs					
**6**	6	No SCNAs					
	6*	No SCNAs					
**7**	7	No SCNAs					
	7*	No SCNAs					
**8**	8	chr12	57547550	60210056	2.66	0.31	CDK4
			68816107	69211314	0.40	0.51	NA,MDM2(partial)
	8*	chr2	14591441	14875017	0.28	1.42	FAM84A,LOC653602
			188288530	190550757	2.26	1.39	TFPI,GULP1,MIR561,DIRC1,COL3A1,MIR1245A,MIR1245B,MIR3606,COL5A2, MIR3129,WDR75,SLC40A1,ASNSD1
		chr3	168179613	173717505	5.54	0.91	EIF5A2,PRKCI,MIR569
			178643104	179605366	0.96	0.85	PIK3CA,ZNF639
		chr8	128298805	129204835	0.91	0.63	AC084123.9,MYC
			46898287	48264252	1.37	0.77	LINC00293,LOC100287846
			113348715	113914681	0.57	0.88	MIR2053
		chr12	57491213	60210056	2.72	0.96	CDK4
			68816107	69267705	0.45	1.59	MDM2
			69550788	70233087	0.68	0.43	YEATS4
		chr17	37571480	37627836	0.06	0.65	NA,MED1(partial)

			37627837	38022528	0.39	0.83	ERBB2,GRB7,MIEN1,PGAP3,PNMT,PPP1R1B,STARD3,TCAP
**9**	9	No SCNAs					
	9*	No SCNAs					
**10**	10	chr5	0	239082	0.24	0.83	PLEKHG4B,LRRC14B,CCDC127
		chr7	54418576	56004247	1.59	1.43	EGFR
			56004248	56690412	0.69	1.34	MRPS17,GBAS,PSPH,CCT6A,SNORA15,SUMF2,PHKG1,CHCHD2, NUPR1L,LOC650226,LOC100240728,DKFZp434L192,LOC101928401
		chr12	57716559	58170037	0.45	2.32	CDK4
			69154979	69663554	0.51	2.30	MDM2
			26116981	26286020	0.17	2.09	RASSF8,BHLHE41
			31383503	31608970	0.23	2.10	FAM60A,FLJ13224
			39815506	40041620	0.23	2.00	ABCD2
			48598423	48827205	0.23	1.97	H1FNT,ZNF641
			52898910	53180883	0.28	2.40	KRT5,KRT71,KRT74,KRT72,KRT73,KRT2,KRT1,KRT77,KRT76
			55273282	55442847	0.17	1.92	TESPA1,NEUROD4
			55849053	56416552	0.57	2.00	OR6C70,OR6C68,OR6C4,OR2AP1,OR10P1,METTL7B,ITGA7, BLOC1S1,BLOC1S1-RDH5,RDH5,CD63,GDF11,SARNP,ORMDL2, DNAJC14,TMEM198B,MMP19,WIBG,DGKA,PMEL,CDK2,RAB5B, SUOX
			63377355	63546596	0.17	1.07	AVPR1A
			66159120	66328414	0.17	2.07	HMGA2
			67346377	67966852	0.62	2.16	CAND1,LOC100507175
		chr14	36123886	38953950	2.83	2.09	NKX2-1,NKX2-8,PAX9
			32333941	32672167	0.34	1.74	ARHGAP5-AS1,ARHGAP5
			33293656	34534132	1.24	2.13	NPAS3,EGLN3
			34534133	35446244	0.91	2.01	SPTSSA,EAPP,SNX6,CFL2,BAZ1A,IGBP1P1
			35446245	36123885	0.68	2.52	SRP54,FAM177A1,LOC101927178,PPP2R3C,KIAA0391,PSMA6, NFKBIA,INSM2
			41280740	42809511	1.53	1.96	LOC644919,LRFN5
		chr20	50983604	53586631	2.60	0.56	ZNF217
	10*	chr12	58113702	58170037	0.06	0.34	CDK4
			69154979	69663554	0.51	0.67	MDM2

			57716559	58113701	0.40	0.67	INHBC,INHBE,GLI1,ARHGAP9,MARS,MIR6758,DDIT3,MBD6,DCTN2,KIF5A,PIP4K2,DTX3,ARHGEF25,SLC26A10,B4GALNT1
		chr14	36236691	38953950	2.72	0.59	NKX2-1,NKX2-8,PAX9
			35502579	36236690	0.73	0.75	FAM177A1,LOC101927178,PPP2R3C,KIAA0391,PSMA6,NFKBIA, INSM2
**11**	11		150439458	150665017	0.23	3.38	TARS2,MIR6878,ECM1,LINC00568,ADAMTSL4,MIR4257,ADAMTSL4-AS1,MCL1,ENSA
			151060970	151511766	0.45	3.60	SEMA6C,TNFAIP8L2,TNFAIP8L2,SCNM1,LYSMD1,SCNM1,TMOD4,VPS72,PIP5K1A,PSMD4,ZNF687,PI4KB,RFX5,SELENBP1,PSMB4,POGZ,CGN
			152191656	152648934	0.46	3.20	FLG,FLG-AS1,FLG2,CRNN,LCE5A,CRCT1,LCE3E,LCE3D,LCE3C, LCE3B,LCE3A,LCE2D
			155475790	155590731	0.11	1.25	ASH1L-AS1,MSTO1
			160351906	161087036	0.74	3.48	VANGL2,SLAMF6,CD84,SLAMF1,CD48,SLAMF7,LY9,CD244,ITLN1,LOC101928372,ITLN2,F11R,TSTD1,USF1,ARHGAP30,PVRL4,KLHDC9
		chr3	9515053	10362289	0.85	0.23	IRAK2
		chr6	32426373	32596130	0.17	1.42	HLA-DRB5,HLA-DRB6,HLA-DRB1
		chr7	54644223	55378598	0.73	1.33	EGFR
			97500468	99649407	2.15	0.21	ARPC1A,LMTK2,SMURF1
			99649408	101701091	2.05	0.29	EPHB4
			155112576	159071691	3.96	0.48	SHH
		chr9	30946253	36098297	5.15	0.47	TESK1
		chr12	53462793	54026937	0.56	0.45	AMHR2,MAP3K12
		chr14	34252139	34931907	0.68	1.14	EGLN3,SPTSSA
			34931908	36010930	1.08	1.28	EAPP,SNX6,CFL2,BAZ1A,IGBP1P1,SRP54,FAM177A1,LOC10192717,PPP2R3C, KIAA0391,PSMA6,NFKBIA,INSM2
			36010931	36974377	0.96	1.15	BRMS1L,LINC00609,PTCSC3,MBIP
		chr19	30237393	30350059	0.11	1.20	CCNE1
			40069921	40705127	0.64	1.93	NA,MAP3K10(partial)
			18624424	18737265	0.11	1.53	FKBP8,KXD1,UBA52,C19orf60,CRLF1,TMEM59L
			18962682	19244349	0.28	1.67	GDF1,CERS1,COPE,DDX49,HOMER3,SUGP2,ARMC6,SLC25A42

			19526025	19807845	0.28	1.32	TSSK6,NDUFA13,YJEFN3,CILP2,PBX4,LPAR2,GMIP,ATP13A1, ZNF101
			20090334	20203618	0.11	1.52	ZNF682
			20487849	20890359	0.40	1.75	ZNF826P,MIR1270-1,MIR1270-2,ZNF737,ZNF626
			36167794	36336794	0.17	2.19	ZBTB32,KMT2B,IGFLR1,U2AF1L4,PSENEN,LIN37,HSPB6,C19orf55,ARHGAP33,LOC644050,PRODH2
			36336795	36449486	0.11	1.73	KIRREL2,APLP1,NFKBID,HCST,TYROBP,LRFN3
			38145028	38375420	0.23	1.76	ZNF781,ZNF607,ZNF573,LOC644554,LOC100631378
			39900919	39957252	0.06	1.23	PLEKHG2,RPS16
			39957253	40069920	0.11	2.36	TIMM50,DLL3,SELV,EID2B,EID2
	11*	chr1	150439458	150665017	0.23	1.88	TARS2,MIR6878,ECM1,LINC00568,ADAMTSL4,MIR4257,ADAMTSL4-AS1,MCL1,ENSA
			151060970	151511766	0.45	1.92	SEMA6C,TNFAIP8L2,TNFAIP8L2-SCNM1,LYSMD1,SCNM1, TMOD4,VPS72,PIP5K1A,PSMD4,ZNF687,PI4KB,RFX5,SELENBP1,PSMB4,POGZ,CGN
			152191656	152648934	0.46	1.77	FLG,FLG-AS1,FLG2,CRNN,LCE5A,CRCT1,LCE3E,LCE3D,LCE3C, LCE3B,LCE3A,LCE2D
			160351906	161087036	0.74	1.98	VANGL2,SLAMF6,CD84,SLAMF1,CD48,SLAMF7,LY9,CD244,ITLN1,LOC101928372,ITLN2,F11R,TSTD1,USF1,ARHGAP30,PVRL4,KLHDC9
		chr6	32426373	32596130	0.17	1.19	HLA-DRB5,HLA-DRB6,HLA-DRB1
		chr7	54757037	55378598	0.62	1.00	EGFR
		chr19	39788252	40705127	0.92	0.82	MED29,MAP3K10(partial)
**12**	12	No SCNAs					
	12*	chr7	53392415	55718015	2.33	0.75	EGFR
		chr12	57320242	58282710	0.96	0.78	CDK4
**13**	13	chr7	97953419	99137182	1.18	0.46	ARPC1A,SMURF1
			99593066	102366663	2.77	0.46	EPHB4
	13*	No SCNAs					
**14**	14	chr17	45176973	45289779	0.11	0.85	CDC27
	14*	No SCNAs					
**15**	15	chr17	45176973	45289779	0.11	1.02	CDC27
		chrX	153388870	153561789	0.17	0.92	OPN1LW,OPN1MW,OPN1MW2,TEX28,TKTL1

	15*	chr17	45176973	45289779	0.11	0.99	CDC27
		chrX	153388870	153561789	0.17	1.02	OPN1LW,OPN1MW,OPN1MW2,TEX28,TKTL1
**16**	16	chr9	21556676	25629785	4.07	-0.71	CDKN2A
		chr14	36519443	39010284	2.49	2.36	NKX2-1,NKX2-8,PAX9
			27740983	28251358	0.51	1.68	LINC00645
			31314198	32218310	0.90	2.14	COCH,LOC100506071,STRN3,MIR624,AP4S1,HECTD1,HEATR5A, [illegible]
			32390341	32786058	0.40	2.21	ARHGAP5-AS1,ARHGAP5
			33350210	34931907	1.58	2.30	NPAS3,EGLN3,SPTSSA
			34931908	35898083	0.97	2.36	EAPP,SNX6,CFL2,BAZ1A,IGBP1P1,SRP54,FAM177A1,LOC10192717,PPP2R3C, [illegible]
			35898084	36519442	0.62	2.44	INSM2,RALGAPA1,RALGAPA1P,BRMS1L
			45079244	45592622	0.51	2.23	C14orf28,LOC101927418,KLHL28,FAM179B,PRPF39,SNORD127
			49845212	50183975	0.34	2.38	RPS29,LRR1,RPL36AL,MGAT2,DNAAF2,POLE2
			50183976	50579202	0.40	2.33	KLHDC2,NEMF,ARF6,MIR6076,C14orf182,LOC100506499,C14orf183
			50579203	51142810	0.56	2.27	SOS2,L2HGDH,ATP5S,CDKL1,MAP4K5,ATL1,SAV1
			51142811	51481240	0.34	2.35	NIN,ABHD12B,PYGL
			51481241	52274595	0.79	2.25	TMX1,LINC00640,FRMD6-AS2,FRMD6-AS1,FRMD6
			52274596	52613002	0.34	2.34	GNG2,C14orf166,NID2
			52613003	53009821	0.40	2.49	PTGDR,PTGER2
			53009822	53348299	0.34	2.35	GPR137C,ERO1L,PSMC6,STYX,GNPNAT1
			53348300	54535933	1.19	1.86	DDHD1,LOC101927620,MIR5580,BMP4
			54593176	55044876	0.45	2.22	CDKN3,CNIH1,GMFB,CGRRF1
		chr17	37402243	38473909	1.07	3.51	CDC6,ERBB2,GRB7,STARD3
			37345718	37402242	0.06	1.81	RPL19,STAC2
	16*	chr14	36519443	39010284	2.49	1.98	LINC00609,PTCSC3,MBIP,SFTA3,NKX2-1,NKX2-1-AS1,NKX2-8,PAX9,SLC25A21,MIR4503,SLC25A21-AS1,MIPOL1,FOXA1, SSTR1,CLEC14A
			31314198	32446717	1.13	1.78	COCH,LOC100506071,STRN3,MIR624,AP4S1,HECTD1,HEATR5A, LOC101927124,DTD2,GPR33,NUBPL
			32446718	32559451	0.11	2.14	ARHGAP5-AS1
			33350210	34988448	1.64	1.92	NPAS3,EGLN3,SPTSSA
			34988449	36519442	1.53	2.05	SNX6,CFL2,BAZ1A,IGBP1P1,SRP54,FAM177A1,LOC101927178,PPP2R3C,KIAA0391,PSMA6,NFKBIA,INSM2,RALGAPA1,RALGAPA1P,BRMS1L

			45079244	45592622	0.51	1.83	C14orf28,LOC101927418,KLHL28,FAM179B,PRPF39,SNORD127
			50861046	52613002	1.75	1.90	MAP4K5,ATL1,SAV1,NIN,ABHD12B,PYGL,TRIM9,TMX1,LINC00640,FRMD6-AS2,FRMD6-AS1,FRMD6,GNG2,C14orf166,NID2
			52613003	53348299	0.74	2.09	PTGDR,PTGER2,TXNDC16,GPR137C,ERO1L,PSMC6,STYX, GNPNAT1
			54649510	55044876	0.40	1.89	CDKN3,CNIH1,GMFB,CGRRF1
		chr17	37684382	37966170	0.28	2.95	NEUROD2,PPP1R1B,STARD3,TCAP,PNMT,PGAP3,ERBB2,MIR4728,MIEN1,GRB7,IKZF3
			37966171	38473909	0.51	3.10	ZPBP2,GSDMB,ORMDL3,LRRC3C,GSDMA,PSMD3,CSF3,MED24, MIR6884,SNORD124,THRA,NR1D1,MSL1,CASC3,MIR6866, RAPGEFL1,MIR6867,WIPF2,CDC6
			37345718	37402242	0.06	1.66	RPL19,STAC2
			37402243	37684381	0.28	3.26	FBXL20,MED1
**17**	17	No SCNAs					
	17*	chr17	45176973	45289779	0.11	0.76	CDC27
**18**	18	chr17	45176973	45289779	0.11	0.90	CDC27
	18*	chr17	45176973	45289779	0.11	0.84	CDC27
**19**	19	No SCNAs					
	19*	No SCNAs					
**20**	20	No SCNAs					
	20*	chr17	45176973	45289779	0.11	0.70	CDC27
**21**	21	No SCNAs					
	21*	No SCNAs					
**22**	22	No SCNAs					
	22*	No SCNAs					
**23**	23	chr7	55038885	56060589	1.02	1.04	EGFR
			15353023	16655917	1.30	1.72	MEOX2,LOC101927524,ISPD,ISPD-AS1,SOSTDC1,LRRC72
			17334113	18744242	1.41	1.86	AHR,LOC102659288,LOC101927630,SNX13,PRPS1L1,HDAC9
			19027001	19765651	0.74	1.86	TWIST1,FERD3L,TWISTNB,MIR3146
			19765652	20217877	0.45	1.96	LOC101927668,MACC1-AS1
			20217878	20612973	0.40	1.85	LOC101927769,LOC101927811,ITGB8

		chr17	58312660	59277962	0.97	0.63	APPBP2,PPM1D,USP32(partial)
			59277963	60808896	1.53	0.79	INTS2
			61485397	61767576	0.28	0.53	TACO1
			61767577	62552523	0.78	0.64	CEP95
		chr20	51997923	52681106	0.68	0.62	ZNF217
	23*	chr7	13993380	15409364	1.42	1.28	NA,ETV1(partial)
			55095239	56060589	0.97	0.94	NA,EGFR(partial)
			15409365	16317271	0.91	1.52	MEOX2,LOC101927524,ISPD-AS1,
			17615909	20612973	3.00	1.58	SNX13,PRPS1L1,HDAC9,TWIST1,FERD3L,TWISTNB,MIR3146, TMEM196,LOC101927668,MACC1,MACC1-AS1,LOC101927769, LOC101927811,ITGB8
		chr17	58312660	59334320	1.02	0.51	APPBP2,PPM1D,USP32(partial)
			59334321	60865263	1.53	0.67	INTS2
			60865264	62552523	1.69	0.51	CEP95,TACO1
		chr20	50927261	52568416	1.64	0.44	ZNF217
**24**	24	No SCNAs					
	24*	No SCNAs					
**25**	25	chr8	39182096	39407967	0.23	0.77	ADAM3A
	25*	chr14	36519443	38841142	2.32	1.10	NKX2-1,NKX2-8,PAX9
			34704704	36123885	1.42	0.99	SPTSSA,EAPP,SNX6,CFL2,BAZ1A,IGBP1P1,SRP54,FAM177A1, LOC101927178,PPP2R3C,KIAA0391,PSMA6,NFKBIA,INSM2
			36123886	36519442	0.40	1.19	BRMS1L
**26**	26	chr9	26646711	28001712	1.36	0.49	TEK
			34174447	36605693	2.43	0.47	TESK1
		chr13	48929326	49103046	0.17	-0.58	NA,RB1(partial)
		chr19	39618249	40986795	1.37	1.28	AKT2,MAP3K10,MED29
			32609733	33682835	1.07	0.98	ZNF507,LOC400684,DPY19L3,PDCD5,ANKRD27,RGS9BP,NUDT19,TDRD12,S LC7A9,CEP89,C19orf40,RHPN2,GPATCH1,WDR88
			33682836	34415252	0.73	1.11	LRP3,SLC7A10,CEBPA,CEBPA-AS1,CEBPG,PEPD,CHST8,KCTD15
			35828655	35886068	0.06	1.20	MIR5196,FFAR1,FFAR3

			35886069	36393128	0.51	1.38	LOC100128682,FFAR2,KRTDAP,DMKN,SBSN,GAPDHS, LOC100506469,TMEM147,ATP4A,LOC102723617,HAUS5,RBM42, ETV2,COX6B1,UPK1A,UPK1A-AS1,ZBTB32,KMT2B,IGFLR1, U2AF1L4,PSENEN,LIN37,HSPB6,C19orf55,ARHGAP33, LOC644050,PRODH2,NPHS1,KIRREL2,APLP1,NFKBID
			36393129	36878079	0.48	1.51	HCST,TYROBP,LRFN3,SDHAF1,SYNE4,ALKBH6,LOC101927572, CLIP3,THAP8,WDR62,OVOL3,POLR2I,TBCB,CAPNS1,COX7A1, ZNF565,ZNF146,LOC100134317,LINC00665,ZFP14
			36878080	36934568	0.06	1.26	ZFP82,LOC644189
	26*	chr8	42969171	46898286	3.93	1.24	HGSNAT,POTEA
		chr9	26646711	28058060	0.14	0.58	TEK
			35194471	35815743	0.62	0.71	TESK1
			28742259	28967787	0.23	0.55	MIR876,MIR873
			34230794	35194470	0.96	0.55	KIF24,NUDT2,KIAA1161,C9orf24,FAM219A,DNAI1,ENHO, CNTFR,CNTFR-AS1,RPP25L,DCTN3,ARID3C,SIGMAR1,GALT, IL11RA,CCL27,CCL19,CCL21,FAM205A,FAM205B,KIAA1045,DNAJB5-AS1,DNAJB5,C9orf131,VCP,FANCG,PIGO,STOML2, FAM214B
			35815744	35928596	0.11	0.43	FAM221B,TMEM8B,LINC00950,OR13J1,HRCT1,LINC00961
			35928597	36605693	0.68	0.63	OR2S2,RECK,GLIPR2,CCIN,CLTA,GNE,RNF38
		chr13	48929326	49103046	0.17	-0.87	NA,RB1(partial)
		chr19	39674582	41043181	1.37	1.63	AKT2,MAP3K10,MED29
			32666067	34415252	1.75	1.49	ZNF507,LOC400684,DPY19L3,PDCD5,ANKRD27,RGS9BP,NUDT19,TDRD12,SLC7A9,CEP89,C19orf40,RHPN2,GPATCH1,WDR88,LRP3,SLC7A10,CEBPA,CEBPA-AS1,CEBPG,PEPD,CHST8,KCTD15
			34415253	35659506	1.24	1.39	LSM14A,KIAA0355,GPI,PDCD2L,UBA2,WTIP,SCGB1B2P,SCGB2B2,SCGB2B3P,ZNF302,ZNF181,ZNF599,LOC400685,LINC00904, LOC102723513,ZNF30,ZNF792,GRAMD1A,SCN1B,HPN,HPN-AS1, FXYD3,MIR6887,LGI4,FXYD1,FXYD7
			35659507	35998736	0.34	1.53	FAM187B,LSR,USF2,HAMP,MAG,CD22,MIR5196,FFAR1,FFAR3, LOC100128682,FFAR2,KRTDAP,DMKN
			35998737	36111459	0.11	1.82	SBSN,GAPDHS,LOC100506469,TMEM147,ATP4A,LOC102723617

			36111460	36934568	0.82	2.04	RBM42,ETV2,COX6B1,UPK1A,UPK1A_AS1,ZBTB32,KMT2B,IGFLR1,U2AF1L4,PSENEN,LIN37,HSPB6,C19orf55,ARHGAP33,LOC644050,PRODH2,NPHS1,KIRREL2,APLP1,NFKBID,HCST,TYROBP,LRFN3,SDHAF1,SYNE4,ALKBH6,LOC101927572,CLIP3,THAP8,WDR62,OVOL3,POLR2I,TBCB,CAPNS1,COX7A1,ZNF565,ZNF146,LOC100134317,LINC00665,ZFP14,ZFP82,LOC644189
			36934569	36991061	0.06	1.79	ZNF566,LOC728752
			36991062	39674581	2.68	1.45	ZNF260,ZNF529,LOC101927599,ZNF382,ZNF461,LOC101927621,ZNF567,ZNF850,ZNF790-AS1,ZNF790,ZNF345,ZNF829,ZNF568, ZNF420,ZNF585A,ZNF585B,ZNF383,LOC101927667,LOC284412, HKR1,ZNF527,ZNF569,ZNF570,LOC101927720,ZNF793,ZNF571_AS1, ZNF571,ZNF540,ZFP30,ZNF781,ZNF607,ZNF573,LOC644554, LOC100631378,WDR87,SIPA1L3,DPF1,PPP1R14A,SPINT2,YIF1B, C19orf33,KCNK6,CATSPERG,PSMD8,GGN,SPRED3,FAM98C,RASGRP4,RYR1, MAP4K1,EIF3K,ACTN4,CAPN12,LGALS7,LGALS7B,LGALS4,ECH1,HNRNPL, RINL,SIRT2,NFKBIB,CCER2,SARS2,MRPS12,FBXO17,FBXO27,PAPL,PAK4
**27**	27	No SCNAs					
	27*	chr17	45176973	45289779	0.11	0.81	CDC27
**28**	28	No SCNAs					
	28*	No SCNAs					
**29**	29	chr2	85119767	86024003	0.90	0.85	TMSB10,KCMF1,TCF7L1,TGOLN2,RETSAT,ELMOD3,CAPG,SH2D6, LOC100630918,MAT2A,GGCX,VAMP8,VAMP5,RNF181,TMEM150A, C2orf68,USP39,SFTPB,GNLY,ATOH8,MIR6071
		chr7	54474918	56116956	1.64	1.36	EGFR
			92231257	92907958	0.68	1.03	CDK6
			91381609	91553008	0.17	1.03	MTERF
			91553009	92231256	0.68	1.12	AKAP9,CYP51A1,LRRD1,KRIT1,ANKIB1,GATAD1,PEX1,RBM48, MGC16142,FAM133B,FAM133DP
			92907959	94099873	1.19	0.92	CALCR,MIR653,MIR489,MIR4652,TFPI2,GNGT1,GNG11,BET1, COL1A2
		chr12	41854653	42876859	1.02	0.84	GXYLT1,YAF2,ZCRB1,PPHLN1
		chr18	18590631	20116145	1.53	0.50	GATA6
			20116146	21753616	1.64	0.67	LOC101927571,MIR4741,RBBP8,CABLES1,TMEM241,RIOK3,C18orf8,NPC1,ANKRD29,LAMA3,LOC102724246,TTC39C,CABYR
		chr20	50419843	52342767	1.92	0.82	ZNF217
	29*	chr17	45176973	45289779	0.11	1.00	CDC27

**30**	30	chr7	52825739	56862069	4.04	0.49	EGFR
		chr12	69607124	71482606	1.88	0.32	YEATS4
	30*	chr1	40087630	40655933	0.57	4.14	MYCL
			18566360	19299174	0.73	1.51	KLHDC7A,PAX7,TAS1R2,ALDH4A1,MIR4695,IFFO2
			19299175	20088700	0.79	2.22	UBR4,EMC1,MRTO4,AKR7L,AKR7A3,LOC100506730,AKR7A2,PQLC,CAPZB, MINOS1,MINOS1-NBL1,RPS14P3,NBL1,HTR6
			31047207	31499264	0.45	2.99	MATN1,MATN1-AS1,LAPTM5,MIR4420,SDC3,SNORD103A, [illegible]
			36303741	36585623	0.28	3.91	AGO1,AGO3,TEKT2,ADPRHL2,COL8A2
			36585624	36754743	0.17	2.36	TRAPPC3,MAP7D1
			38447433	38729217	0.28	4.10	FHL3,UTP11L,POU3F1,MIR3659,LOC339442
			39971759	40087629	0.12	3.44	PPIEL,PABPC4,SNORA55
			57290346	57628492	0.34	4.01	C8A,C8B
			79039778	79437608	0.40	4.22	IFI44L,IFI44
			84162012	85296735	1.13	1.69	MIR548AP,TTLL7,PRKACB,SAMD13,UOX,DNASE2B,RPF1,GNG5, CTBS,C1orf180,SSX2IP
		chr11	75651697	77289526	1.64	2.36	ACER3,C11orf30,PAK1,PRKRIR
			77289527	77686213	0.40	3.48	AAMDC,AQP11,CLNS1A,RSF1,INTS4(partial)
			77686214	78250401	0.56	4.65	ALG8,KCTD21,NDUFC2,USP35,INTS4(partial),NARS2(partial)
			11458557	11683959	0.23	2.92	MIR4299
			35857446	36139214	0.28	4.20	MIR3973
		chr14	23766448	23822881	0.06	1.20	BCL2L2
			23822882	25077933	1.26	0.90	RIPK3
			21450951	21677061	0.23	1.43	METTL17,LOC101929718,SLC39A2,NDRG2,MIR6717,TPPP2, RNASE13, RNASE7,RNASE8,ARHGEF40,ZNF219,TMEM253,OR5AU1,LINC00641
			21677062	22184192	0.51	1.54	HNRNPC,RPGRIP1,SUPT16H,CHD8,SNORD9,SNORD8,RAB2B, TOX4,METTL3,SALL2,OR10G3,OR10G2,OR4E2
			22184193	23086916	0.90	1.14	DAD1,ABHD4
			23145482	23766447	0.62	1.50	OXA1L,SLC7A7,MRPL52,MMP14,LRP10,REM2,RBM23,PRMT5, LOC101926933,HAUS4,MIR4707,AJUBA,C14orf93,PSMB5,PSMB11, CDH24,ACIN1,C14orf119,CEBPE,SLC7A8,C14orf164,HOMEZ
			25077934	25813277	0.74	0.82	GZMB,STXBP6
			29046662	29780282	0.73	1.17	FOXG1,C14orf23
			50014810	50240309	0.23	2.22	RPS29,LRR1,RPL36AL,MGAT2,DNAAF2,POLE2,KLHDC1

			50240310	50353085	0.11	2.48	NEMF
			50353086	50917420	0.56	2.18	ARF6,MIR6076,C14orf182,LOC100506499,C14orf183,VCPKMT,SOS2,L2HGDH, ATP5S,CDKL1
			50917421	51199158	0.28	2.09	ATL1,SAV1
			51424907	52274595	0.85	1.67	TRIM9,TMX1,LINC00640,FRMD6-AS2,FRMD6-AS1,FRMD6
			52274596	52727437	0.45	1.43	GNG2,C14orf166,NID2
			52727438	53009821	0.28	1.58	PTGDR,PTGER2
			69529176	69923596	0.39	1.55	EXD2,GALNT16,ERH
			69923597	70881788	0.96	1.80	PLEKHD1,CCDC177,KIAA0247,LOC100289511,SRSF5,SLC10A1, SMOC1,SLC8A3,ADAM21P1,COX16
			72699400	73151070	0.45	2.13	MIR7843
		chr16	49178359	51491575	2.31	-0.67	CYLD
		chr17	56984378	58877401	1.89	0.77	APPBP2,CLTC,DHX40,HEATR6,PPM1D,PTRH2,RNFT1,RPS6KB1, TRIM37,TUBD1,USP32,VMP1
			21983967	25271509	3.29	1.27	MTRNR2L1
		chr22	0	16474074	16.47	2.21	BMS1P17,BMS1P18,POTEH,POTEH-AS1,OR11H1
**31**	31	chr7	99705750	101701091		0.25	EPHB4
	31*	chr17	45176973	45289779	0.11	0.89	CDC27
**32**	32	chr7	54926062	56116956	1.19	0.60	EGFR
	32*	chr7	54869720	56173298	1.30	0.65	EGFR
		chr17	37063527	39150309	2.09	0.29	CASC3,CDC6,ERBB2,FBXL20,GRB7,GSDMB,MED1,MED24,MIEN1, ORMDL3,PGAP3,PNMT,PPP1R1B,PSMD3,RPL19,STARD3,TCAP, LASP1(partial)
**33**	33	chr4	10187134	10412620	0.23	0.39	RAF1P1
		chr17	45176973	45289779	0,11	0,88	CDC27
		chr19	54014315	54409280	0,39	0,83	ZNF331,LOC284379,DPRX,MIR512-2,MIR512-1,MIR1323,MIR498, MIR520E,MIR519E,MIR520F,MIR515-1,MIR515-2,MIR519C, MIR1283-1,MIR520A,MIR526B,MIR519B,MIR525,MIR523,MIR518F, MIR520B,MIR518B,MIR526A1,MIR520C,MIR518C,MIR524,MIR517A, MIR519D,MIR5212,MIR520D,MIR517B,MIR520G,MIR516B2,MIR526A, MIR518E,MIR518A1,MIR518D,MIR516B1,MIR518A2,MIR517C, MIR520H,MIR5211,MIR522,MIR519A1,MIR527,MIR516A1,MIR12832, MIR516A2,MIR519A2,MIR371A,MIR371B,MIR372,MIR373,NLRP12, MYADM
	33*	chr16	33945134	34210300	0,27	1,26	LINC00273

		chr19	54014315	54352946	0,34	0,72	ZNF331,LOC284379,DPRX,MIR512-2,MIR5121,MIR1323,MIR498, MIR520E,MIR519E,MIR520F,MIR515-1,MIR515-2,MIR519C, MIR1283-1,MIR520A,MIR526B,MIR519B,MIR525,MIR523,MIR518F, MIR520B,MIR518B,MIR526A1,MIR520C,MIR518C,MIR524,MIR517A,MIR519D, MIR521-2,MIR520D,MIR517B,MIR520G,MIR516B2,MIR526A2, MIR518E,MIR518A1,MIR518D,MIR516B1,MIR518A2,MIR517C, MIR520H,MIR521-1,MIR522,MIR519A1,MIR527,MIR516A1, MIR1283-2,MIR516A2,MIR519A2,MIR371A,MIR371B,MIR372,MIR373, NLRP12
		chr20	26156311	29429049	3,27	0,77	LOC284801,MIR663A
**34**	34	No SCNAs					
	34*	No SCNAs					
**35**	35	No SCNAs					
	35*	chrX	153388870	153561789		0,76	OPN1LW,OPN1MW,OPN1MW2,TEX28,TKTL1
**36**	36	No SCNAs					
	36*	No SCNAs					
**37**	37	chr17	45176973	45289779	0,11	0,67	CDC27
	37*	No SCNAs					
**38**	38	No SCNAs					
	38*	No SCNAs					
**39**	39	chr6	168332123	168557886		0,74	HGC6.3,KIF25-AS1,KIF25,FRMD1
	39*	No SCNAs					
**40**	40	No SCNAs					
	40*	chr7	54.982.404	55.605.006		0,66	EGFR
**41**	41	chr8	98.693.332	98.862.818		0,50	NA,MTDH(partial)
	41*	No SCNAs					
**42**	42	Not evaluable					
	42	chr1	155936746	156275706	0,34	0,80	RAB25
			155824000	155936745	0,11	2,56	SYT11,RIT1,KIAA0907,SNORA42,SC, ARNA4,RXFP4,MIR6738
		chr9	21158356	25911789	4,75	-0,41	CDKN2A
**43**	43	Not evaluable					
	43	chr17	45176973	45289779	0,11	0,75	CDC27

chr – chromosome

*at osimertinib resistance
