# Supplementary material for: Somatic Copy-Number Alterations in Plasma Circulating Tumor DNA from Advanced EGFR-Mutated Lung Adenocarcinoma Patients
Source: Biomolecules. 2021 Apr 21;11(5):618. doi: 10.3390/biom11050618 (PMC8143372; doi:10.3390/biom11050618)
Supplement: Supplementary file 1 [file biomolecules-11-00618-s001.zip › Biomolecules 2021_Table S2.pdf]

	Patient	Chromosome	Start	End	Log2-ratio**
AKT2	Case 26	chr17	39618249	40986795	1.28
	Case 26*	chr17	39674582	41043181	1.63
CDK4	Case 8	chr12	57547550	60210056	0.31
	Case 8*	chr12	57491213	60210056	0.96
	Case 10	chr12	57716559	58170037	2.32
	Case 10*	chr12	58113702	58170037	0.34
	Case 12*	chr12	57320242	58282710	0.78
CDK6	Case 29	chr7	92231257	92907958	1.03
CDKN2A	Case 16	chr9	21556676	25629785	-0.71
EGFR	Case 10	chr7	54418576	56004247	1.43
	Case 11	chr7	54644223	55378598	1.33
	Case 11*	chr7	54757037	55378598	1.0
	Case 12*	chr7	53392415	55718015	0.75
	Case 23	chr7	55038885	56060589	1.04
	Case 23*	chr7	55095239	56060589	0.94
	Case 29	chr7	54474918	56116956	1.36
	Case 30	chr7	52825739	56862069	0.49
	Case 32	chr7	54926062	56116956	0.6
	Case 32*	chr7	54869720	56173298	0.65
	Case 40*	chr7	54982404	55605006	0.66
ERBB2	Case 8*	chr17	37627837	38022528	0.83
	Case 16	chr17	37402243	38473909	3.51
	Case 16*	chr17	37684382	37966170	2.95
	Case 32*	chr17	37063527	39150309	0.29
MDM2	Case 4	chr12	69098642	70635098	0.74
	Case 8	chr12	68816107	69211314	0.51
	Case 8*	chr12	68816107	69267705	1.59
	Case 10	chr12	69154979	69663554	2.3
	Case 10*	chr12	69154979	69663554	0.67
MET	Case 1*	chr7	115580932	117674526	0.38
PIK3CA	Case 8*	chr3	178643104	179605366	0.85
RB1	Case 26	chr13	48929326	49103046	-0.58
	Case 26*	chr13	48929326	49103046	-0.87

*Progression under osimertinib

**Mean log2-ratios for each identified segment
