# Supplementary figures and images for: Somatic Copy-Number Alterations in Plasma Circulating Tumor DNA from Advanced EGFR-Mutated Lung Adenocarcinoma Patients
Source: Biomolecules. 2021 Apr 21;11(5):618. doi: 10.3390/biom11050618 (PMC8143372; doi:10.3390/biom11050618)

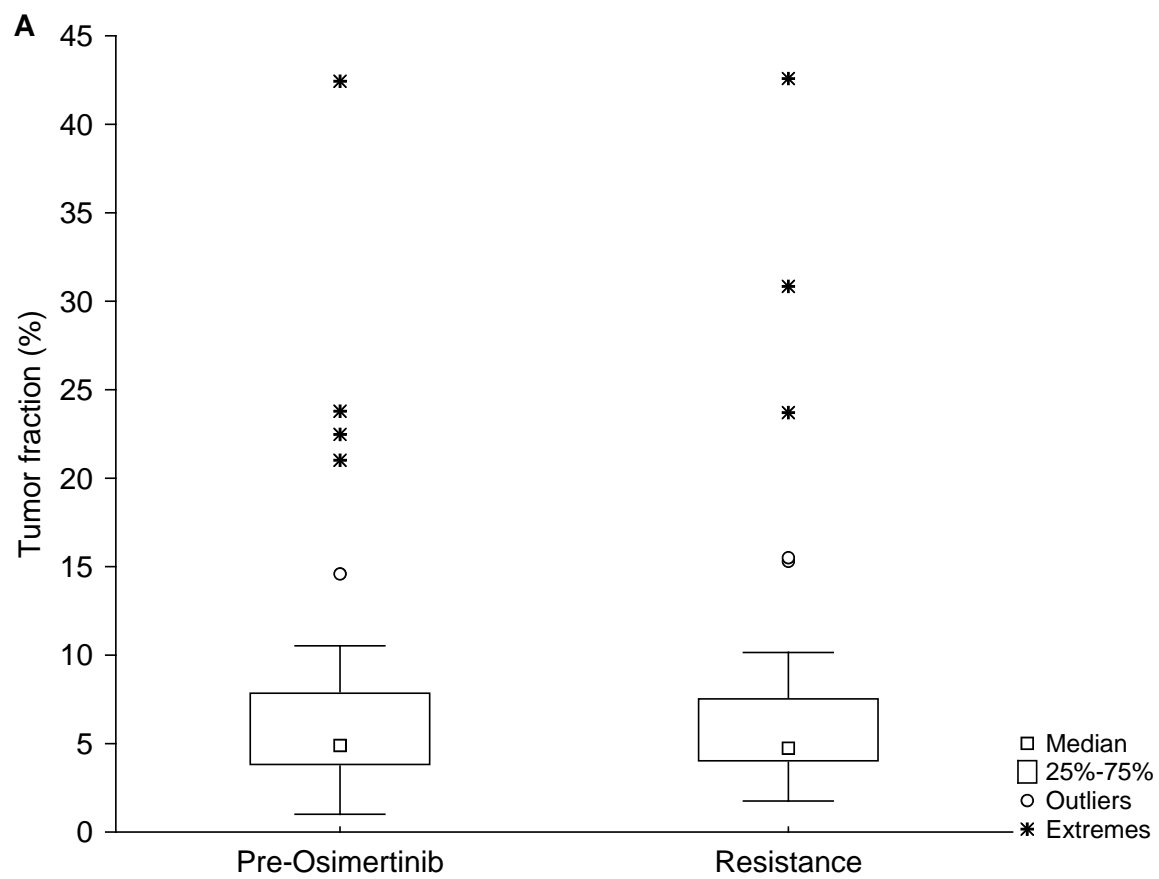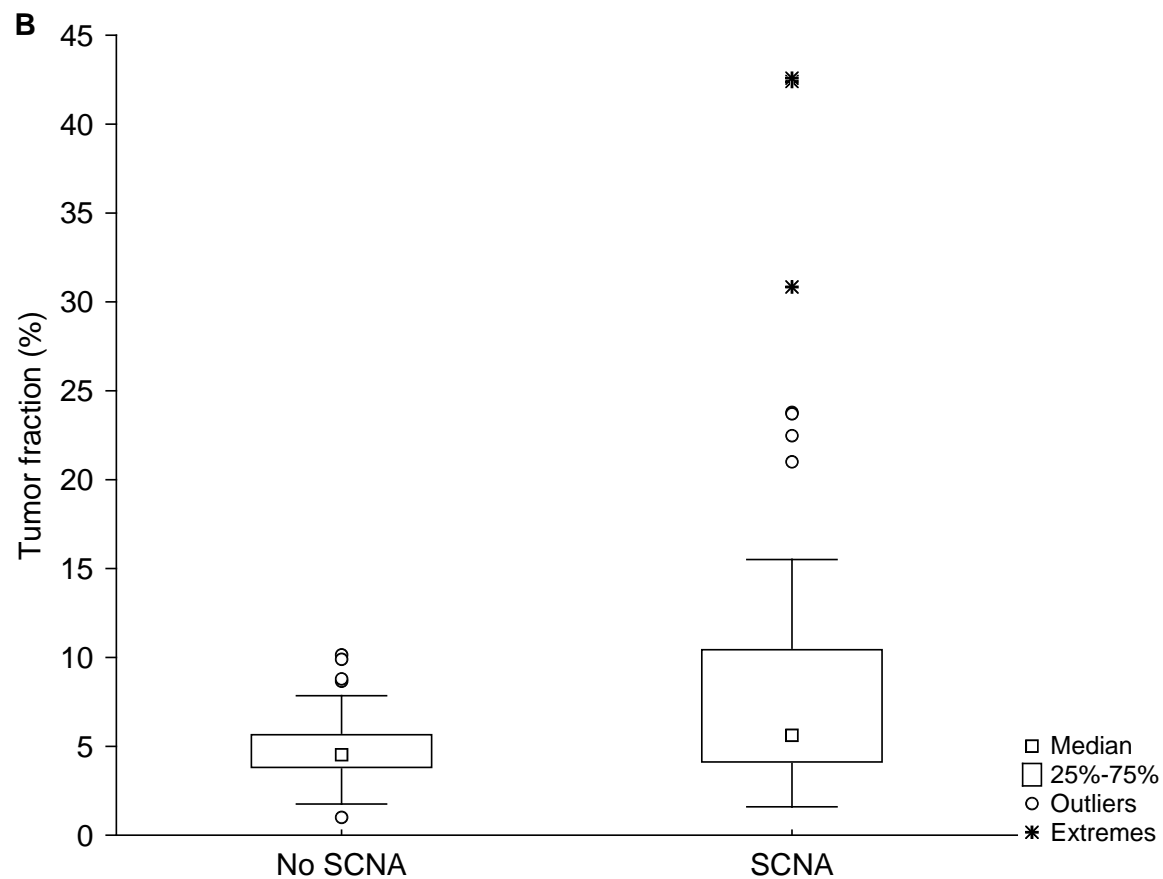

Supplement: Supplementary file 1 [file biomolecules-11-00618-s001.zip › Biomolecules 2021_Figure S2.pdf]

**A**

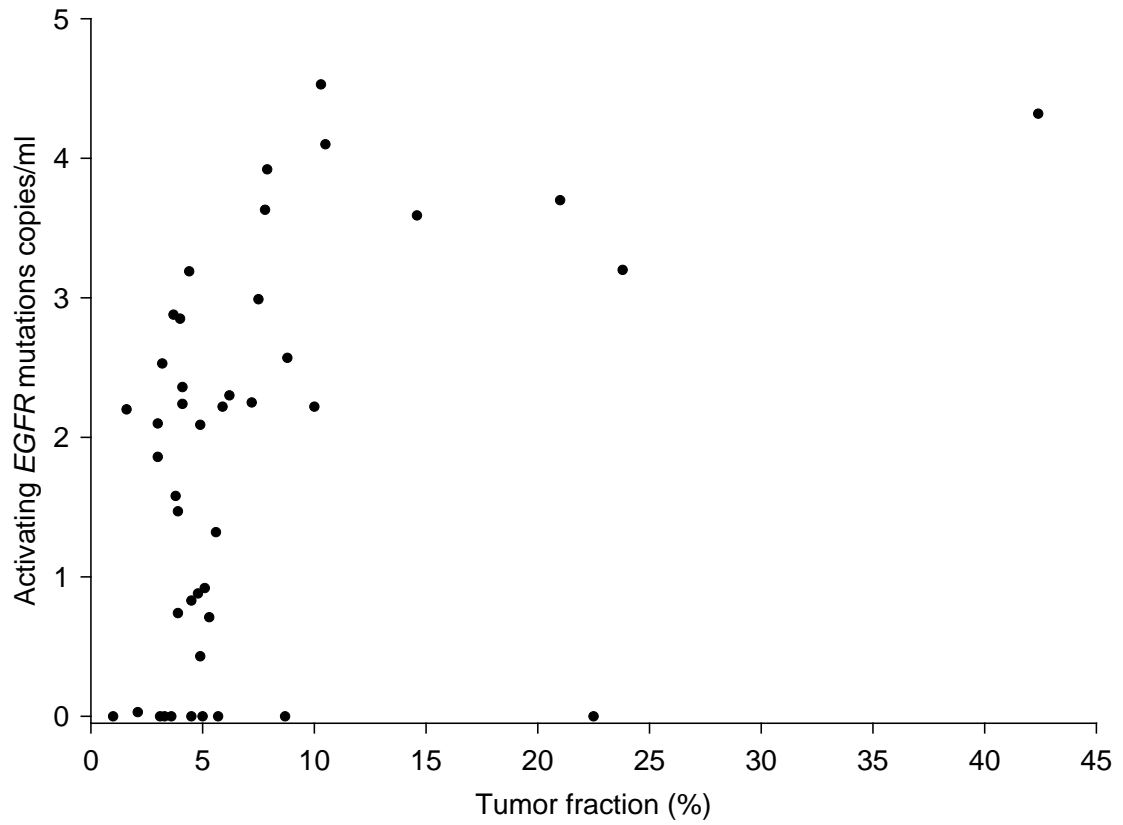

B

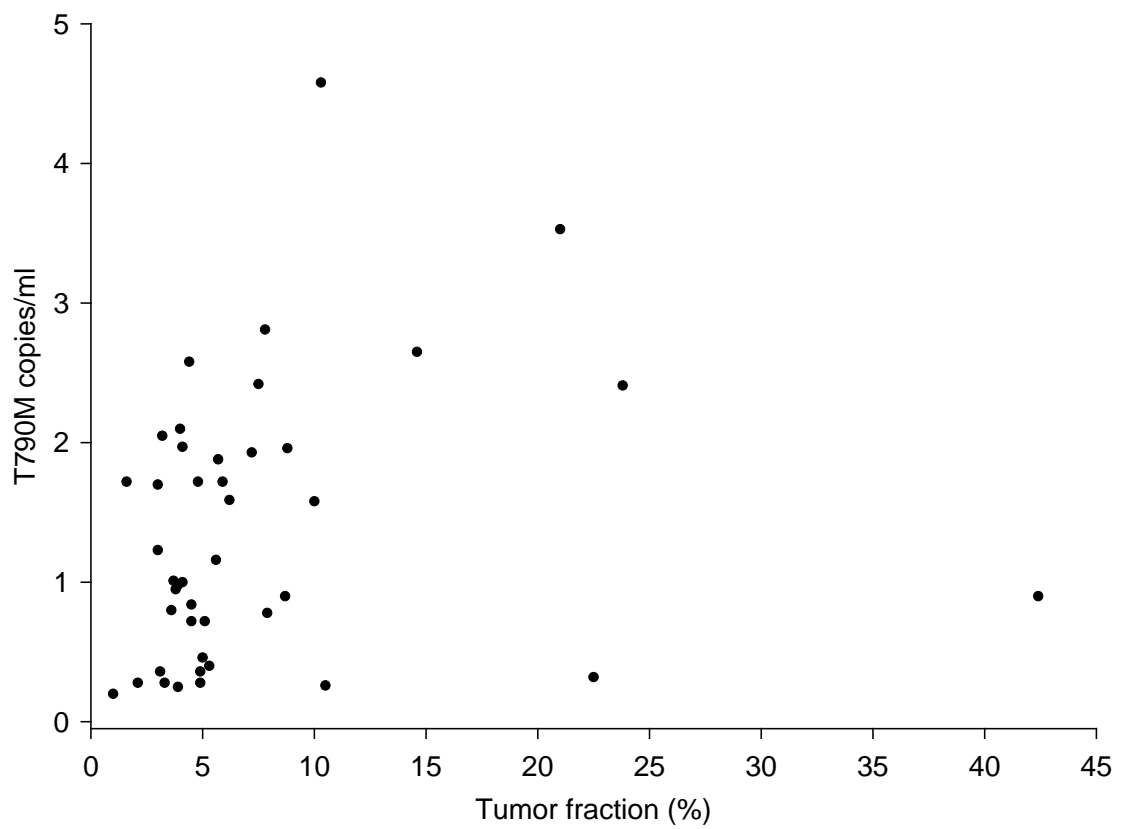

Supplement: Supplementary file 1 [file biomolecules-11-00618-s001.zip › Biomolecules 2021_Figure S3.pdf]

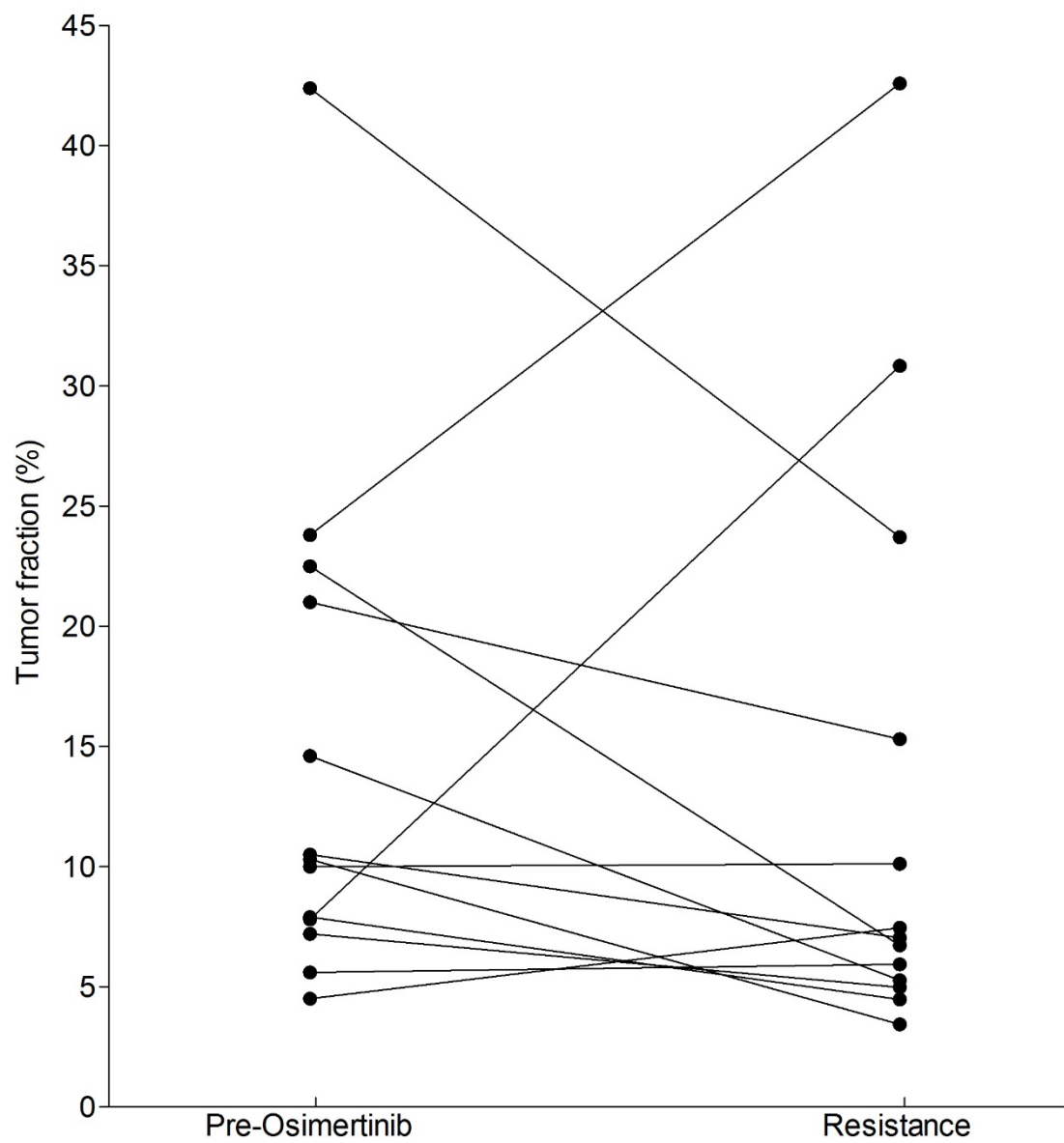

Supplement: Supplementary file 1 [file biomolecules-11-00618-s001.zip › Biomolecules 2021_Figure S4.pdf]

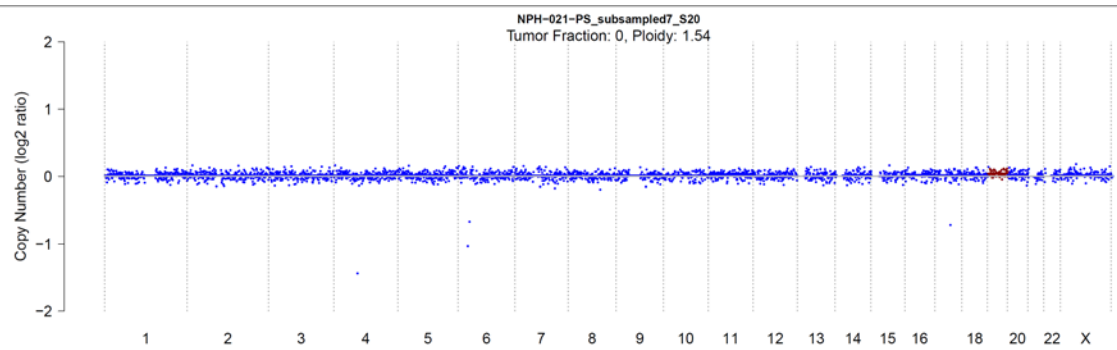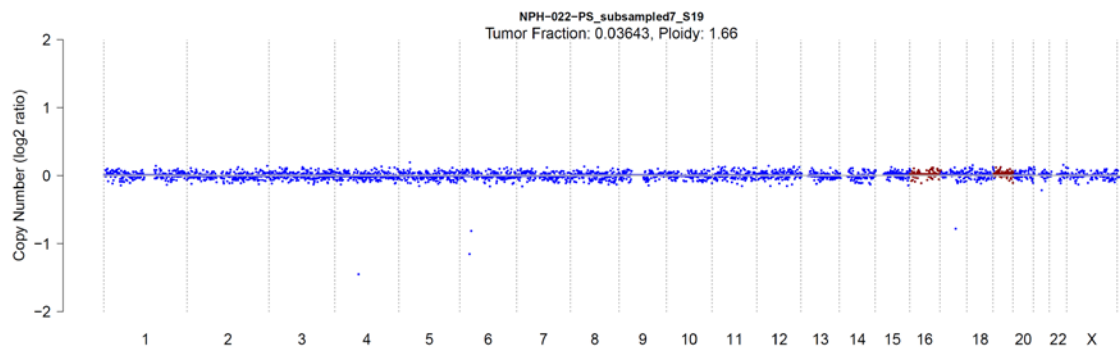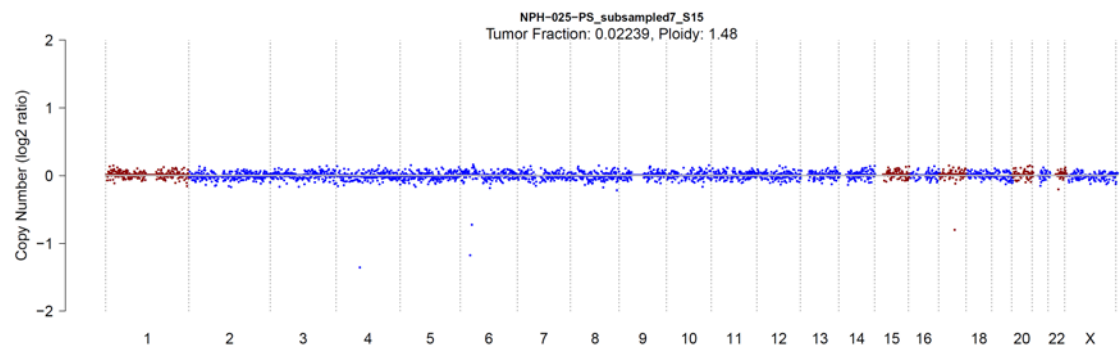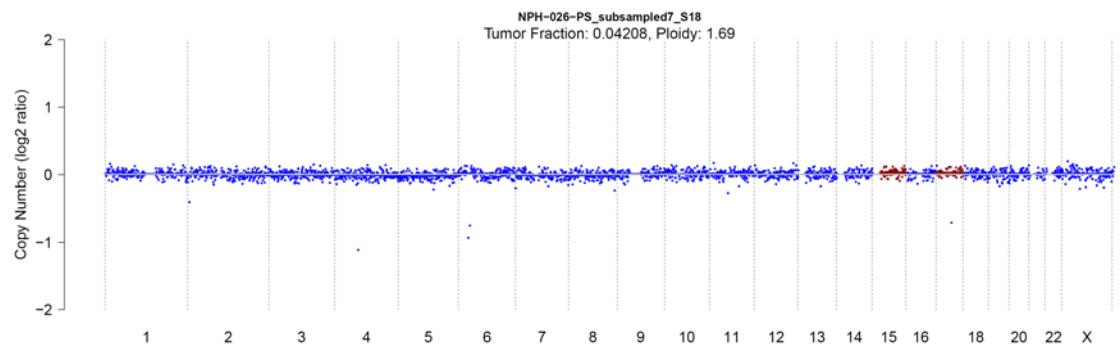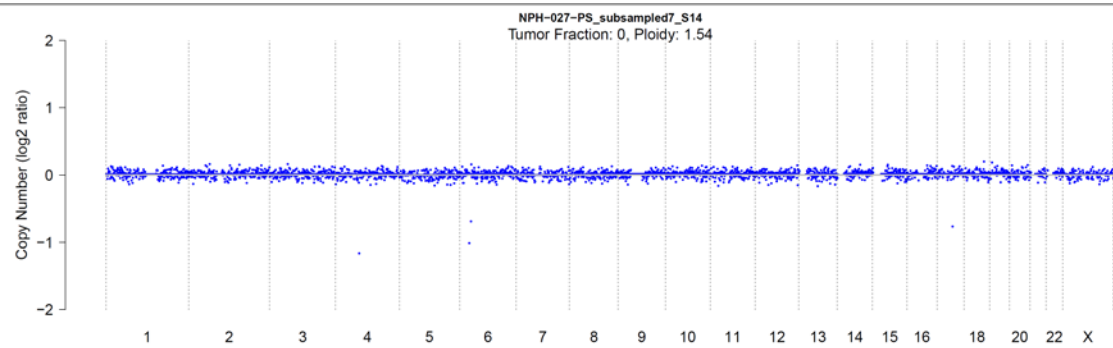

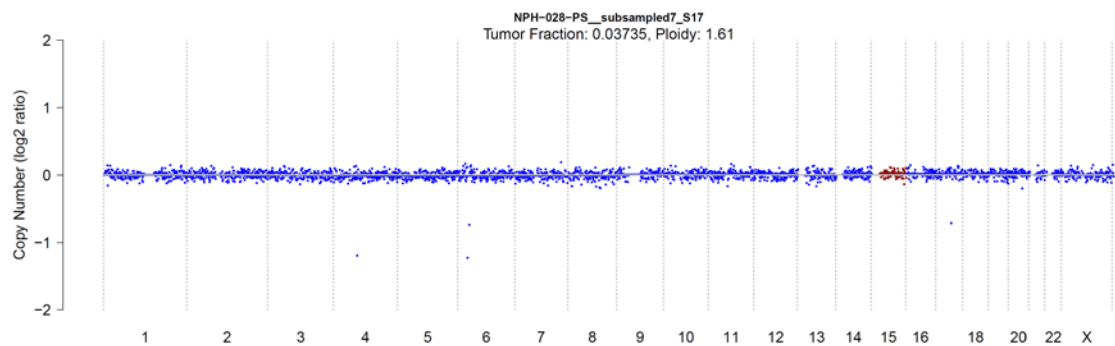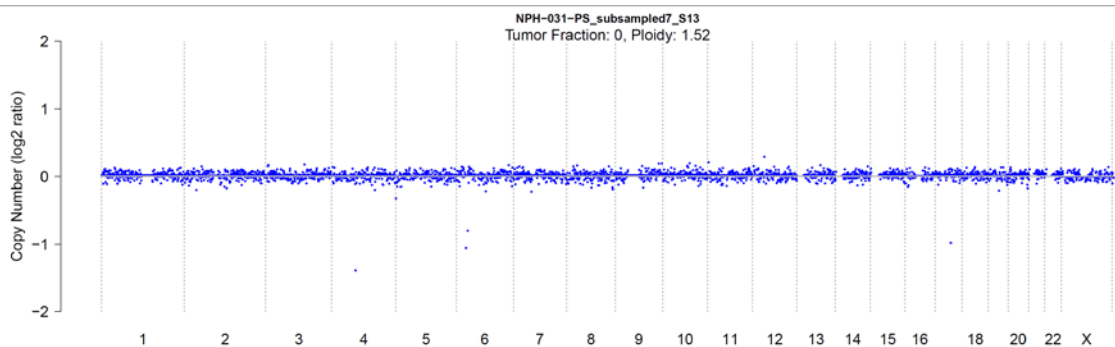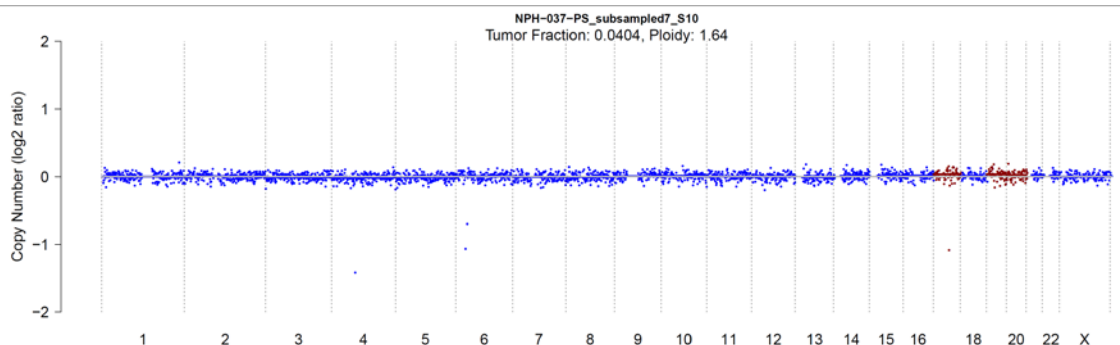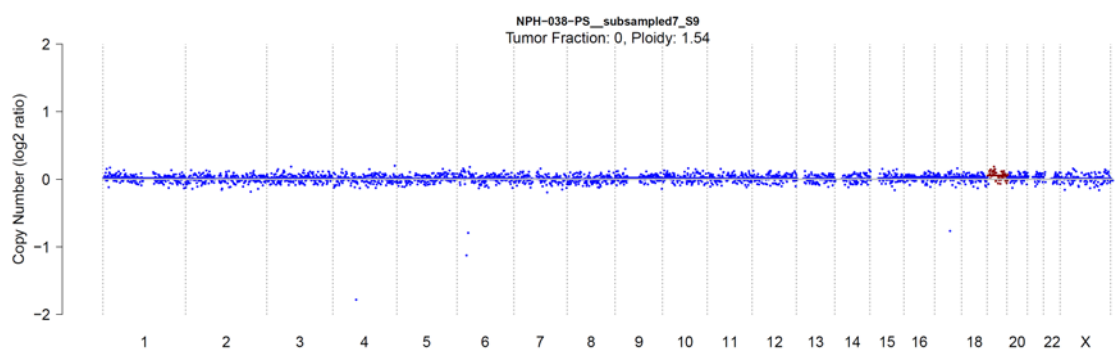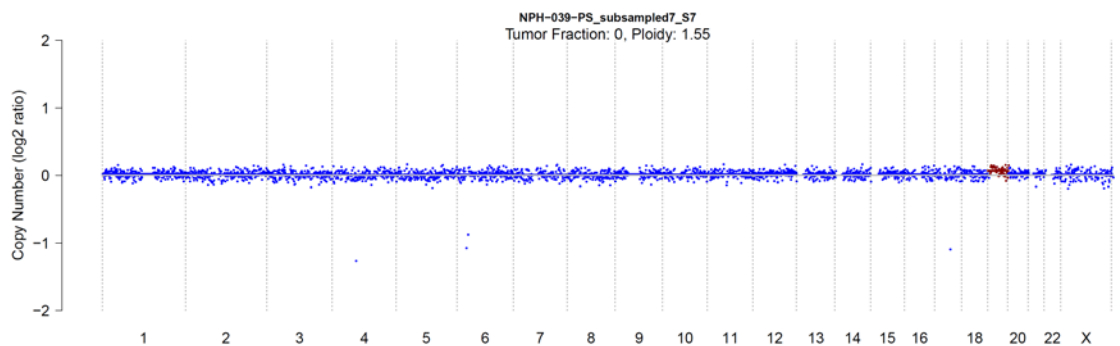

Supplement: Supplementary file 1 [file biomolecules-11-00618-s001.zip › Biomolecules 2021_Figure S5.pdf]
